# Supplementary material for: The malonyl/acetyl-transferase from murine fatty acid synthase is a promiscuous engineering tool for editing polyketide scaffolds
Source: Commun Chem. 2024 Aug 24;7:187. doi: 10.1038/s42004-024-01269-1 (PMC11344766; doi:10.1038/s42004-024-01269-1)
Supplement: Supplementary file 2 — Supplementary Information [file 42004_2024_1269_MOESM2_ESM.pdf]

Supplementary Information for

The malonyl/acetyl-transferase from murine fatty acid synthase is a promiscuous engineering tool for editing polyketide scaffolds

Lynn Buyachuihan [a+], Simon Reiners [a+], Yue Zhao [a], and Martin Grininger[a]*

[a] Institute of Organic Chemistry and Chemical Biology, Buchmann Institute for Molecular Life Sciences, Goethe University Frankfurt, 60438 Frankfurt am Main, Germany
[+]These authors contributed equally to this work
** Corresponding author: grininger@chemie.uni-frankfurt.de*

Content

Supplementary Figures:

Figure S1. The architecture of deoxyerythronolide B synthase (DEBS) along with the catalytic cycle of module 1.
Figure S2. The architecture of pikromycin (PIKS) PKS.
Figure S3. Protein-protein interactions of the hybrid loading modules H1M0 and H2M0.
Figure S4. Sodium dodecyl-sulfate polyacrylamide gel electrophoresis (SDS-PAGE) analysis of purification steps of hybrid loading modules H1M0 and H2M0.
Figure S5. Boundaries of MAT-swapped VEMS constructs M2*-TE.
Figure S6. Boundaries of MAT-swapped VEMS constructs M0-M1*.
Figure S7. SDS-PAGE analysis of the test expression of MAT-swapped VEMS M2*-TE constructs via nickel affinity chromatography (part 1).
Figure S8. SDS-PAGE analysis of the test expression of MAT-swapped VEMS M2*-TE constructs via nickel affinity chromatography (part 2).
Figure S9. SDS-PAGE analysis of the test expression of MAT-swapped VEMS M0-M1* constructs via nickel affinity chromatography.
Figure S10. M1* in VEMS and split VEMS.
Figure S11. SDS-PAGE analysis of the test expression of MAT-swapped M1*(YZ046) via ion exchange and size exclusion chromatography.
Figure S12. Products and possible side products for the reactions of M1* split VEMS shown in Figure 6 with the starter substrates DHBA and 3-hydroxybenzoic acid (HBA).
Figure S13. Products and possible side products for the reactions of M1* split VEMS shown in Figure 6 with the starter substrates acetyl-, propionyl-, and butyryl-CoA.
Figure S14. Design of mono-modular PikAIII- and bimodular DEBS3-based PKS/FAS hybrids.
Figure S15. Purification and quality control of the mono-modular PIKS M5-TE (black) and hybrid PIKS M5 H1-TE (red).
Figure S16. Mass spectra of 10-dml and its derivatives detected by LC-HRMS.
Figure S17. Purification and quality control of bimodular M5-M6-TE (black) and hybrids M5*-M6-TE (red) and M5-M6*-TE (blue).
Figure S18. Proposed reaction pathway to produce 10-dml byproducts from MMalCoA/MalCoA mixtures.
Figure S19. Proposed reaction pathway to produce 10-dml byproduct from MMalCoA/Fluoromethylmalonyl-CoA (FMMalCoA) mixtures.
Figure S20. Mass spectra of 3-hydroxy-narbonolide and its derivatives detected by LC-HRMS.

Figure S21. Quality of the VemG KS1-AT1 didomain structure predicted with ColabFold.
Figure S22. Quality of the VemH KS2-AT2 didomain structure predicted with ColabFold.

Supplementary Tables:
Table S1. Cloning strategy of plasmids generated in this study. Individual fragments were generated by PCR and assembled via In-Fusion cloning.
Table S2. DNA and amino acid sequences of plasmids which are not published elsewhere and were used as a template to generate the plasmids of the constructs used in this study.
Table S3. Plasmids used in this study and their origin.
Table S4. List of all constructs that were cloned and tested for expression in *E. coli*.
Table S5. Amino acid sequences of MAT-swapped VEMS loading modules H1M0 and H2M0.
Table S6. Amino acid sequences of MAT-swapped VEMS M2*-TE constructs.
Table S7. Amino acid sequences of MAT-swapped VEMS M0-M1* and split VEMS M1* constructs.
Table S8. Amino acid sequences of PIKS-based constructs.
Table S9. Amino acid sequences of DEBS-based constructs.
Table S10. Amino acid sequences used for the prediction of the KS-AT didomain structure of VemG KS1-AT1 and KS2-AT2 with ColabFold.

Supplementary Figures

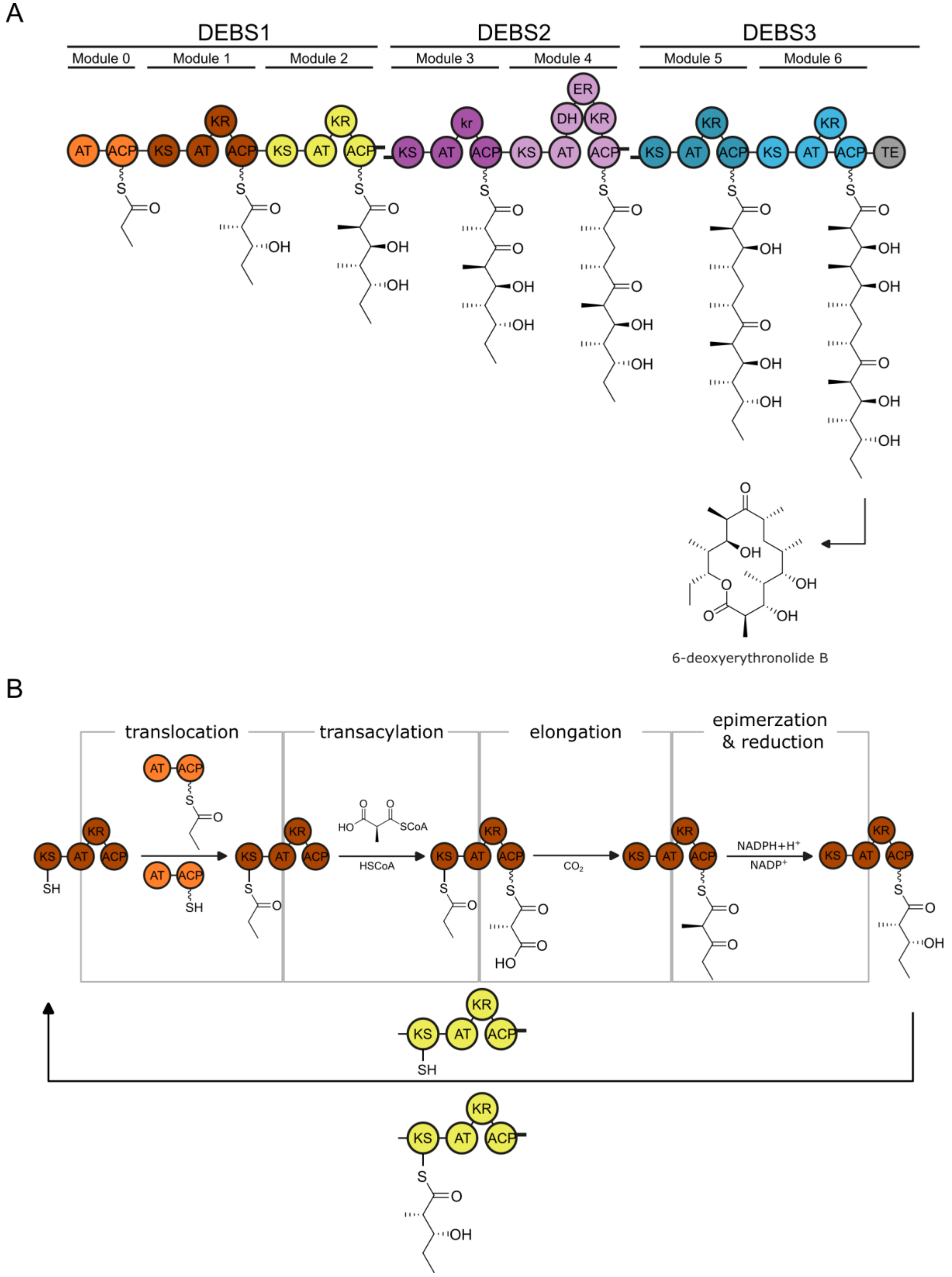

Figure S1. The architecture of deoxyerythronolide B synthase (DEBS) along with the catalytic cycle of module 1. (A) The DEBS polyketide synthase (PKS) comprises three polypeptides, namely DEBS1, DEBS2, and DEBS3. Each polypeptide harbors two elongation modules, while DEBS1 possesses an N-terminal additional loading module (module 0) and DEBS3 possesses a C-terminal thioesterase (TE) domain which facilitates the release of the product 6-deoxyerythronolide B. The polyketide intermediates are illustrated attached to the respective acyl carrier protein (ACP). The docking domains, represented by black tabs, allow for non-covalent interactions between the polypeptides. Domain annotation: KS – ketosynthase, AT – acyltransferase, KR – ketoreductase, kr – non-reductive ketoreductase-like domain, DH – dehydratase, ER – enoylreductase, ACP- acyl carrier protein, and TE – thioesterase. (B) Catalytic cycle demonstrated for module 1. Translocation: Chain translocation from the ACP0 of module 0 to the KS1 of module 1. Transacylation: AT-catalyzed transacylation of ACP1 using (2S)-methylmalonyl-CoA. Elongation: KS-catalyzed decarboxylative Claisen condensation resulting in the elongation of the acyl chain by two carbon units. Epimerization & reduction: KR1 catalyzes epimerization of the C2 methyl group followed by diastereospecific reduction. The reduced intermediate is handed over to KS2 of module 2 and module 1 is ready for the next catalytic cycle.

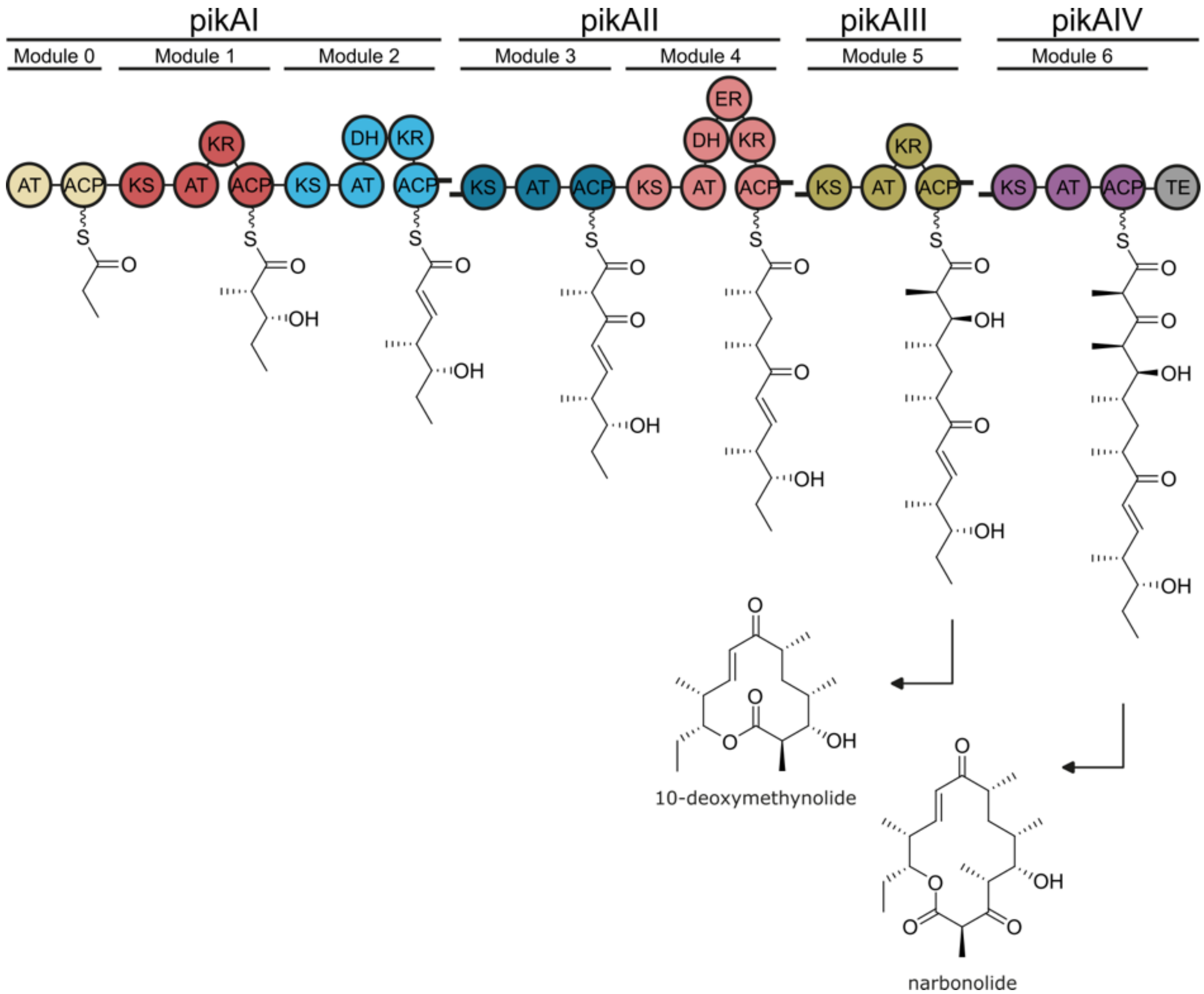

Figure S2. The architecture of pikromycin (PIKS) PKS. The PIKS PKS comprises four polypeptides, namely pikAI, pikAII, pikAIII, and pikAIV. The polyketide intermediates are illustrated attached to the respective ACP. The docking domains, represented by black tabs, allow for non-covalent interactions between the polypeptides. Domain annotation: KS – ketosynthase, AT - acyltransferase, KR - ketoreductase, kr – non-reductive ketoreductase-like domain, DH - dehydratase, ER - enoylreductase, ACP- acyl carrier protein, and TE - thioesterase. PIKS is responsible for the production of narbonolide, the precursor of pikromycin. TE-catalyzed release of the ACP5-bound pentaketide leads to the formation of 10-deoxymethynolide (10-dml), the precursor of methymycin.

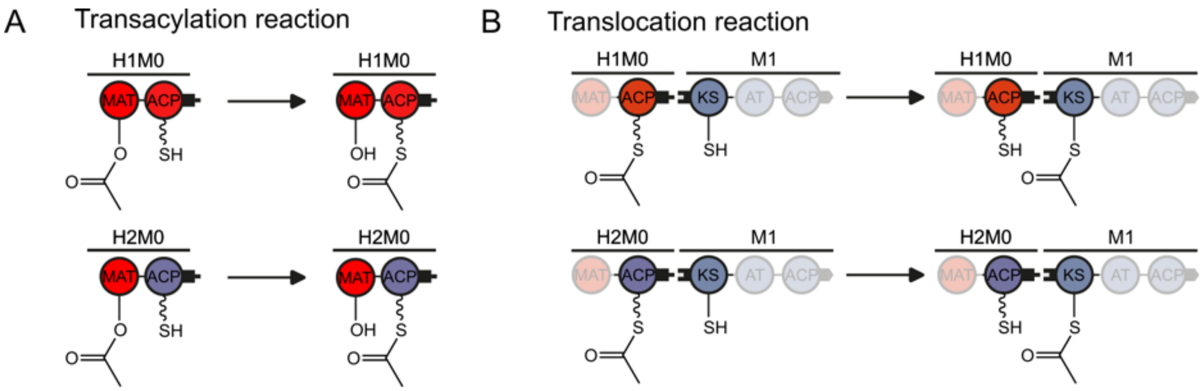

Figure S3. Protein-protein interactions of the hybrid loading modules H1M0 and H2M0. Murine fatty acid synthase (mFAS-derived domains are colored in red. (A) During the transacylation reaction, the malonyl/acetyl-transferase (MAT) domain transfers the starter substrate (here acetyl-CoA) onto its C-terminal ACP. In the case of H1M0 the mFAS MAT transfers the substrate onto the native mFAS ACP, while in the case of H2M0 non-native domain-domain interactions (DDIs) occur, as the mFAS MAT has to interact with the venemycin PKS (VEMS) ACP0. (B) During the translocation reaction, the ACP-bound intermediate is handed over to the KS of the downstream module. In the case of H1M0, non-native DDIs occur, as the mFAS ACP has to interact with the VEMS KS1. For H2M0 the intermediate is translocated from VEMS ACP0 to VEMS KS1, which preserves the native translocation interface.

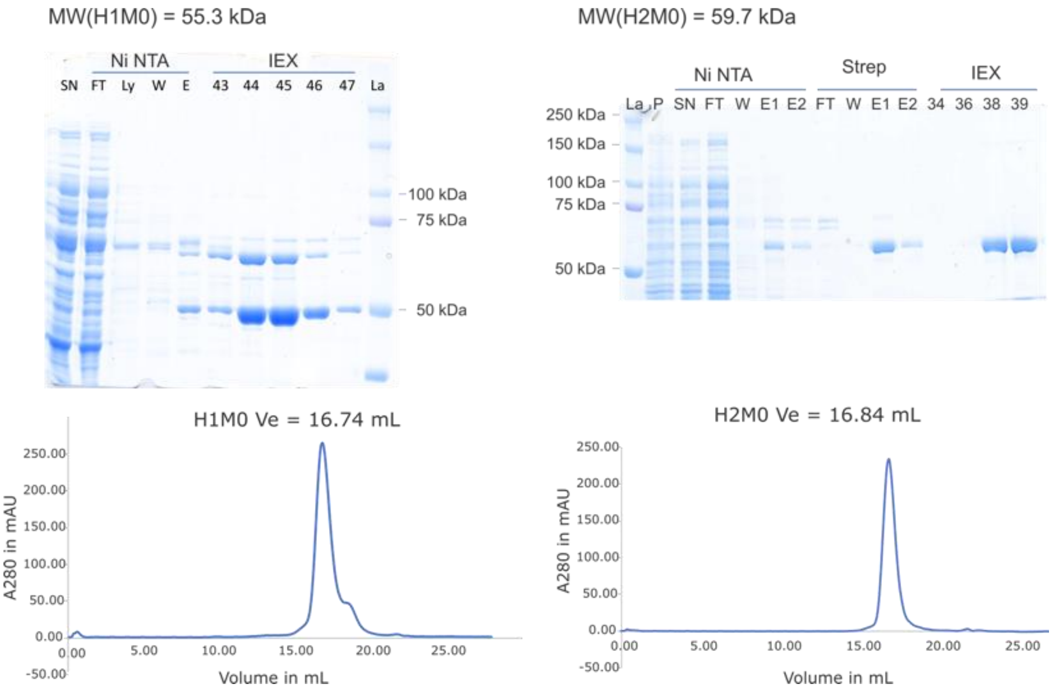

Figure S4. Sodium dodecyl-sulfate polyacrylamide gel electrophoresis (SDS-PAGE) analysis of purification steps of hybrid loading modules H1M0 and H2M0. The H1M0 construct possesses an N-terminal His-tag and was purified via Ni affinity chromatography and subsequent ion exchange chromatography (IEX). The impurities around 70 kDa could not be separated from the H1M0 via IEX. Fractions 44-46 were pooled for the activity assays. The H2M0 construct possesses an N-terminal Twinstrep-tag and a C-terminal His-tag and was purified via Ni affinity, strep, and subsequent IEX. The impurities around 70 kDa could be removed via affinity chromatography using the strep-tag technology. Strep elution was applied to IEX and fractions 38 and 39 were pooled for activity assays and quality control via size exclusion chromatography (SEC). SEC analysis reveals that H2M0 adopts a single oligomeric state eluting at 16.84 mL. Quality control of split VEMS M0 (not MAT swap) can be found in our previous study under construct name LB047[1]. Abbreviations are as follows: La – ladder, P – pellet, SN – supernatant, FT – flowthrough, E – Elution.

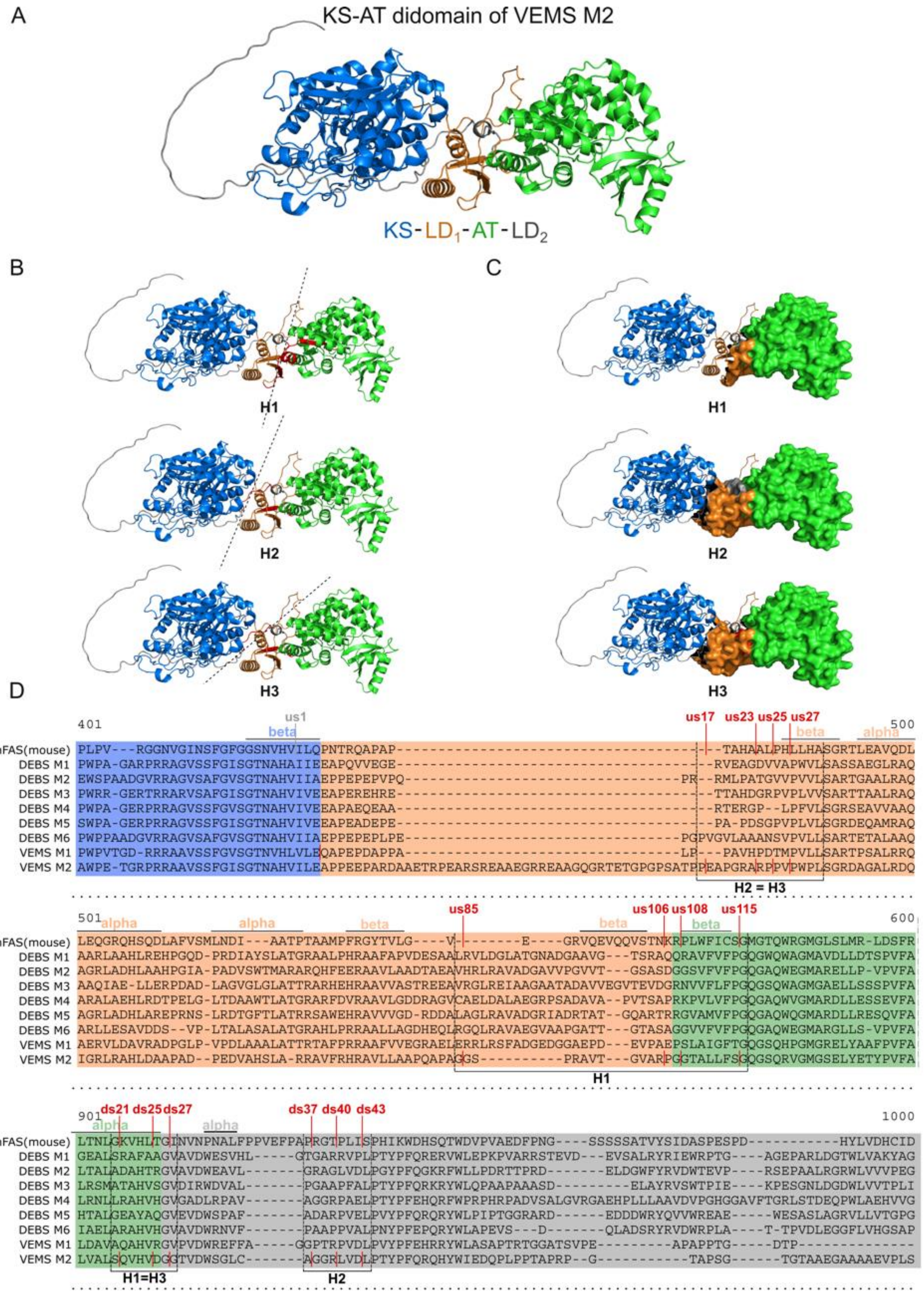
A
KS-AT didomain of VEMS M2
KS-LD1-AT-LD2
B
C
H1
H2
H3
H1
H2
H3
D
401
us1
beta
us17 us23 us25 us27
beta
alpha
500
mFAS(mouse) PLPV---RGGNVGINSFGFGGSNVHVILQPNTRQAPAP---------------------------------------TAHAALPHLLHASGRTLEAVQDL
DEBS M1 PWPA-GARPRRAGVSSFGISGTNAHAIIEEAPQVVEGE--------------------------------------RVEAGDVVAPWVLSASSAEGLRAQ
DEBS M2 EWSPAADGVRRAGVSAFGVSGTNAHVIIAEPPEPEPVPQ---------------------------------PR--RMLPATGVVPVVLSARTGAALRAQ
DEBS M3 PWRR-GERTRRARVSAFGISGTNAHVIVEEAPEREHRE--------------------------------------TTAHDGRPVPLVVSARTTAALRAQ
DEBS M4 PWPA-GERPRRAGVSSFGVSGTNAHVIVEEAPAEQEAA--------------------------------------RTERGP--LPFVLSGRSEAVVAAQ
DEBS M5 SWPA-GERPRRAGVSSFGISGTNAHVIVEEAPEADEPE--------------------------------------PA-PDSGPVPLVLSGRDEQAMRAQ
DEBS M6 PWPPAADGVRRAGVSAFGVSGTNAHVIIAEPPEPEPLPE---------------------------------PGPVGVLAAANSVPVLLSARTETALAAQ
VEMS M1 PWPVTGD-RRRAAVSSFGVSGTNVHLVLEQAPEPDAPPA---------------------------------LP---PAVHPDTMPVLLSARTPSALRRQ
VEMS M2 AWPE-TGRPRRAAVSSFGISGTNAHVILEAPPEEPARDAAETRPEARSREAAEGRREAAGQGRTETGPGPSATPPEAPGRARPPVPWPLSGRDAGALRDQ
H2 = H3
501
alpha
alpha
beta
us85
beta
us106 us108 us115
beta
600
mFAS(mouse) LEQGRQHSQDLAFVSMLNDI---AATPTAAMPFRGYTVLG----V--------E----GRVQEVQQVSTNKRPLWFICSGMGTQWRGMGLSLMR-LDSFR
DEBS M1 AARLAAHLREHPGQD-PRDIAYSLATGRAALPHRAAFAPVDESAALRVLDGLATGNADGAAVG---TSRAQQRAVFVFPGQGWQWAGMAVDLLDTSPVFA
DEBS M2 AGRLADHLAAHPGIA-PADVSWTMARARQHFEERAAVLAADTAEAVHRLRAVADGAVVPGVVT---GSASDGGSVFVFPGQGAQWEGMARELLP-VPVFA
DEBS M3 AAQIAE-LLERPDAD-LAGVGLGLATTRARHEHRAAVVASTREEAVRGLREIAAGAATADAVVEGVTEVDGRNVVFLFPGQGSQWAGMGAELLSSSPVFA
DEBS M4 ARALAEHLRDTPELG-LTDAAWTLATGRARFDVRAAVLGDDRAGVCAELDALAEGRPSADAVA--PVTSAPRKPVLVFPGQGAQWVGMARDLLESSEVFA
DEBS M5 AGRLADHLAREPRNS-LRDTGFTLATRRSAWEHRAVVVGD-RDDALAGLRAVADGRIADRTAT--GQARTRRGVAMVFPGQGAQWQGMARDLLRESQVFA
DEBS M6 ARLLESAVDDS--VP-LTALASALATGRAHLPRRAALLAGDHEQLRGQLRAVAEGVAAPGATT---GTASAGGVVFVFPGQGAQWEGMARGLLS-VPVFA
VEMS M1 AERVLDAVRADPGLP-VPDLAAALATTRTAFPRRAAFVVEGRAELERRLRSFADGEDGGAEPD---EVPAEPSLAIGFTGQGSQHPGMGRELYAAFPVFA
VEMS M2 IGRLRAHLDAAPAD--PEDVAHSLA-RRAVFRHRAVLLAAPQAPAGGS----------PRAVT---GVARPGGTALLFSGQGSQRVGMGSELYETYPVFA
H1
901
ds21 ds25 ds27
alpha
alpha
ds37 ds40 ds43
1000
mFAS(mouse) LTNLGKVHLTGINVNPNALFPPVEFPAPRGTPLISPHIKWDHSQTWDVPVAEDFPNG----SSSSSATVYSIDASPESPD-----------HYLVDHCID
DEBS M1 GEALSRAFAAGVAVDWESVHL-----GTGARRVPLPTYPFQRERVWLEPKPVARRSTEVD---EVSALRYRIEWRPTG----AGEPARLDGTWLVAKYAG
DEBS M2 LTALADAHTRGVAVDWEAVL-------GRAGLVDLPGYPFQGKRFWLLPDRTTPRD-------ELDGWFYRVDWTEVP----RSEPAALRGRWLVVVPEG
DEBS M3 LRSMATAHVSGVDIRWDVAL-------PGAAPFALPTYPFQRKRYWLQPAAPAAASD---------ELAYRVSWTPIE----KPESGNLDGDWLVVTPLI
DEBS M4 LRNLLRAHVHGVGADLRPAV-------AGGRPAELPTYPFEHQRFWPRPHRPADVSALGVRGAEHPLLLAAVDVPGHGGAVFTGRLSTDEQPWLAEHVVG
DEBS M5 HTALGEAYAQGVEVDWSPAF-------ADARPVELPVYPFQRQRYWLPIPTGGRARD------EDDDWRYQVVWREAE-----WESASLAGRVLLVTGPG
DEBS M6 IAELARAHVHGVAVDWRNVF-------PAAPPVALPNYPFEPQRYWLAPEVS---D-------QLADSRYRVDWRPLA----T-TPVDLEGGFLVHGSAP
VEMS M1 LDAVAQAHVRGVPVDWREFFA-----GGPTRPVDLPVYPFEHRRYWLASAPTRTGGATSVPE---------APAPPTG-----DTP--------------
VEMS M2 LVALSQVHVDGGTVDWSGLC-------AGGRLVDLPTYPFQRQHYWIEDQPLPPTAPRP--G-----------TAPSG------TGTAAEGAAAAEVPLS
H1=H3
H2

Figure S5. Boundaries of MAT-swapped VEMS constructs M2*-TE. (A) Structural model of the VEMS M2 KS-AT didomain fold (monomeric) predicted with ColabFold[2]. Quality parameters of the structural prediction are shown in Figure S22. KS, LD_1, AT, and LD_2 domain are depicted in blue, orange, green, and grey cartoon, respectively. (B) MAT-swaps in H1, H2, and H3 group are visualized with dashed lines. The residue range in which the junction sites were chosen are highlighted in red. (C) For each hybrid group (H1, H2, and H3) the swapped part of the didomain is depicted as surface. (D) Multiple Sequence alignment (MSA) of the KS-AT didomain sequences of mFAS and all elongation modules from DEBS and VEMS. Swap junctions upstream of the AT domain are termed usXX (XX is the residue position according to first junction site us1, indicated in the MSA) and downstream of the AT domain dsXX. Secondary structure elements of the predicted structure of the VEMS M2 KS-AT didomain are indicated. The range in which the respective swap junctions of the hybrid groups were chosen (indicated in red) is framed with dashed lines. For module 2, a total of 36 constructs were designed, 12 constructs for each hybrid group (Table S4). Out of these, 31 constructs were cloned, and finally, 24 constructs were subjected to test expression in *Escherichia coli*.

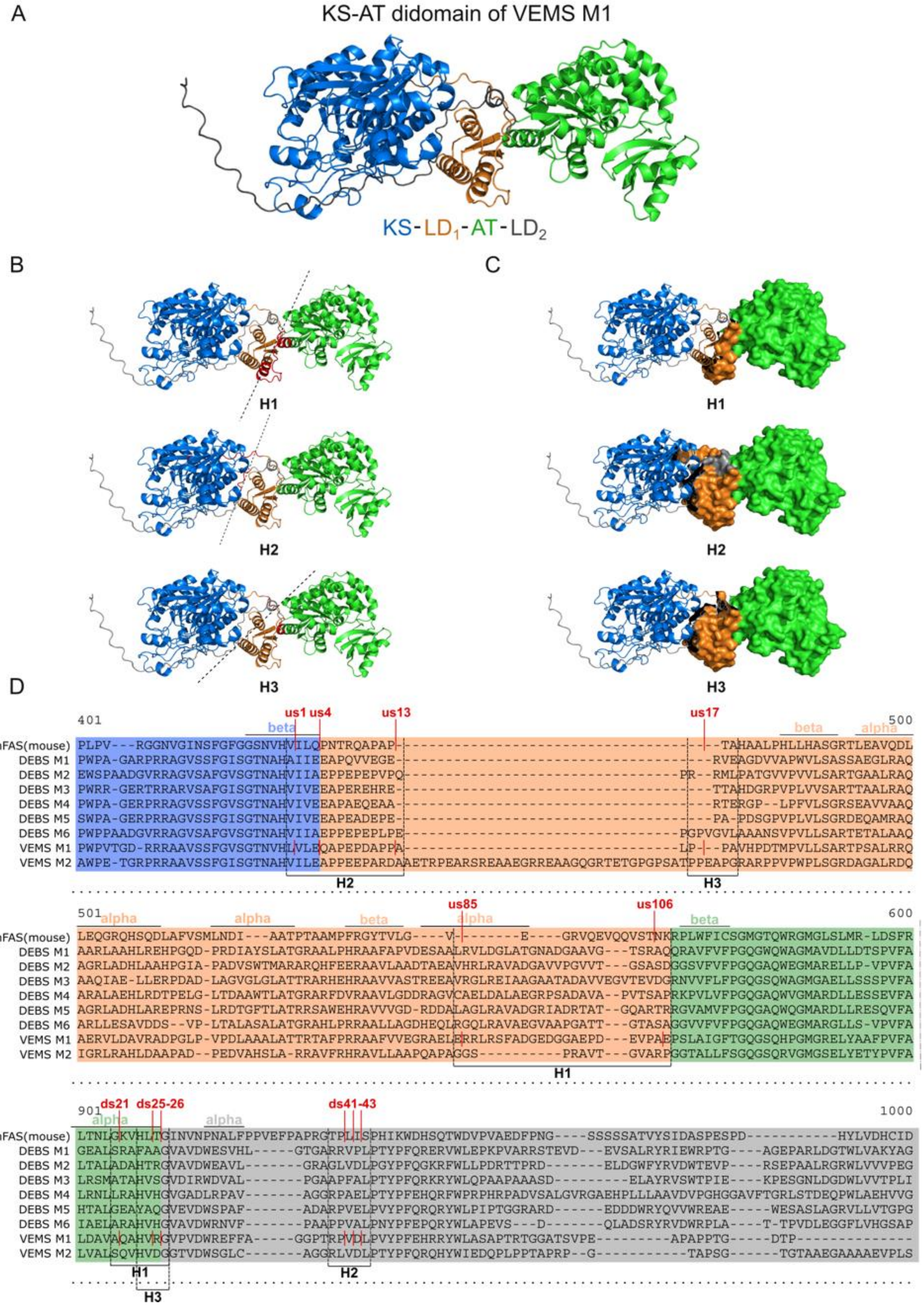
A
KS-AT didomain of VEMS M1
KS-LD1-AT-LD2
B
C
H1
H2
H3
D
us1 us4
us13
us17
us85
us106
ds21 ds25-26
ds41-43
mFAS(mouse)
DEBS M1
DEBS M2
DEBS M3
DEBS M4
DEBS M5
DEBS M6
VEMS M1
VEMS M2

Figure S6. Boundaries of MAT-swapped VEMS constructs M0-M1*. (A) Structural model of the VEMS M1 KS-AT didomain fold (monomeric) predicted with ColabFold[2]. Quality parameters of the structural prediction are shown in Figure S21. KS, LD_1, AT, and LD_2 domain are depicted in blue, orange, green, and grey cartoon, respectively. (B) MAT-swaps in H1, H2, and H3 group are visualized with dashed lines. The residue range in which the junction sites were chosen is highlighted in red. (C) For each hybrid group (H1, H2, and H3) the swapped part of the didomain is depicted as surface. (D) Multiple Sequence alignment (MSA) of the KS-AT didomain sequences of mFAS and all elongation modules from DEBS and VEMS. Swap junctions upstream of the AT domain are termed usXX (XX is the residue position according to first junction site us1, indicated in the MSA) and downstream of the AT domain dsXX. Secondary structure elements of the predicted structure of the VEMS M1 KS-AT didomain are indicated. The range in which the respective swap junctions of the hybrid groups were chosen (indicated in red) is framed with dashed lines. For VemG, a total of eight constructs were designed (four of H1, three of H2, and one of H3 group, Table S4). Out of these, seven constructs were successfully cloned, and four constructs were tested for expression in *E. coli*. However, only one construct (H1 group, YZ038) yielded enough protein for further analysis.

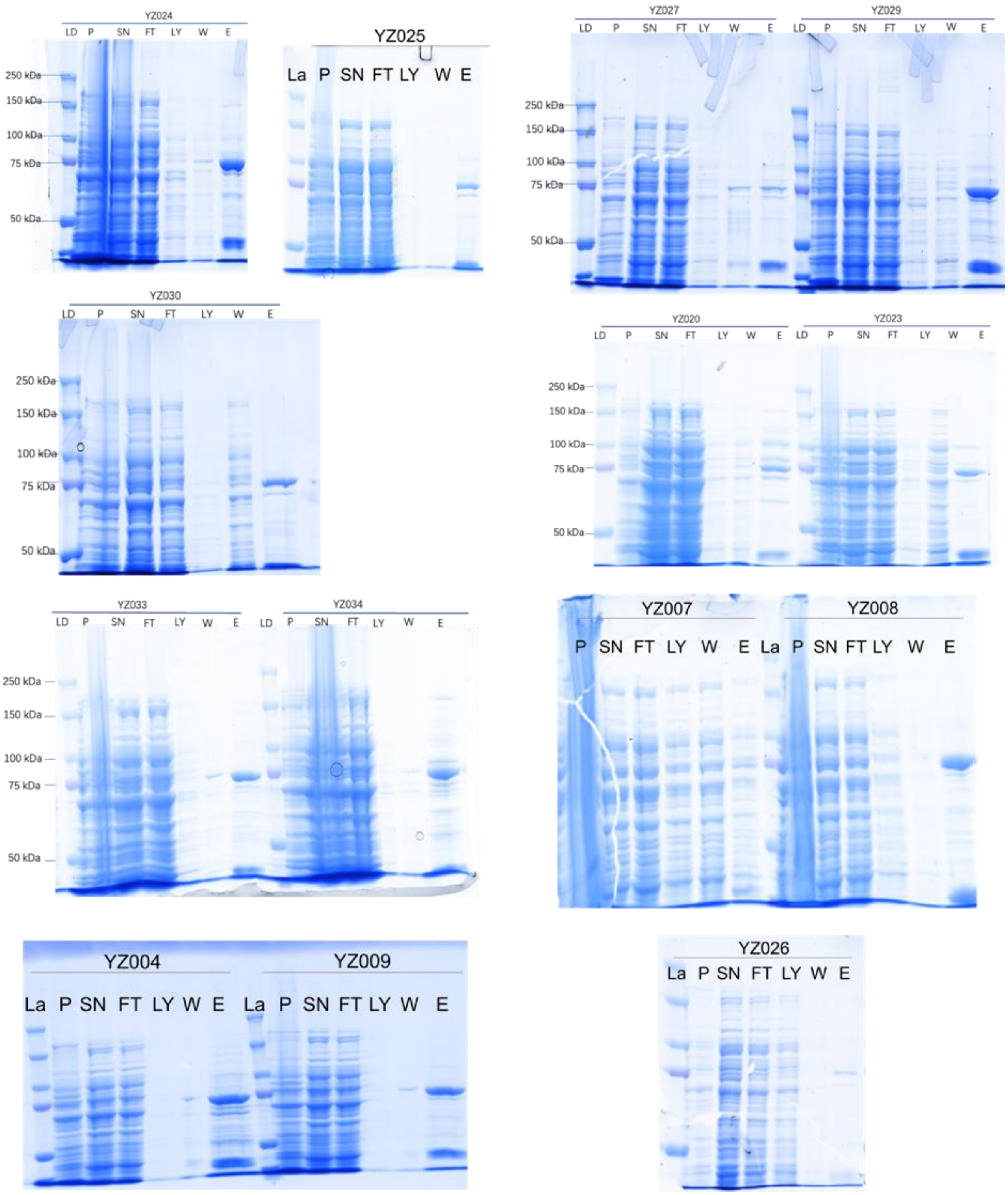

Figure S7. SDS-PAGE analysis of the test expression of MAT-swapped VEMS M2*-TE constructs via nickel affinity chromatography (part 1). The molecular weight of M2-TE constructs is around 145 kDa. Abbreviations are as follows: LD or La – ladder, P – Pellet, FT – Flowthrough, Ly – Lysis. W – Wash, E – Elution. The Precision Plus Protein Dual Color Standard from Bio-Rad was used. Only few constructs show a slight band at the correct molecular weight in the elution fraction. Residual analysis is shown in Figure S8.

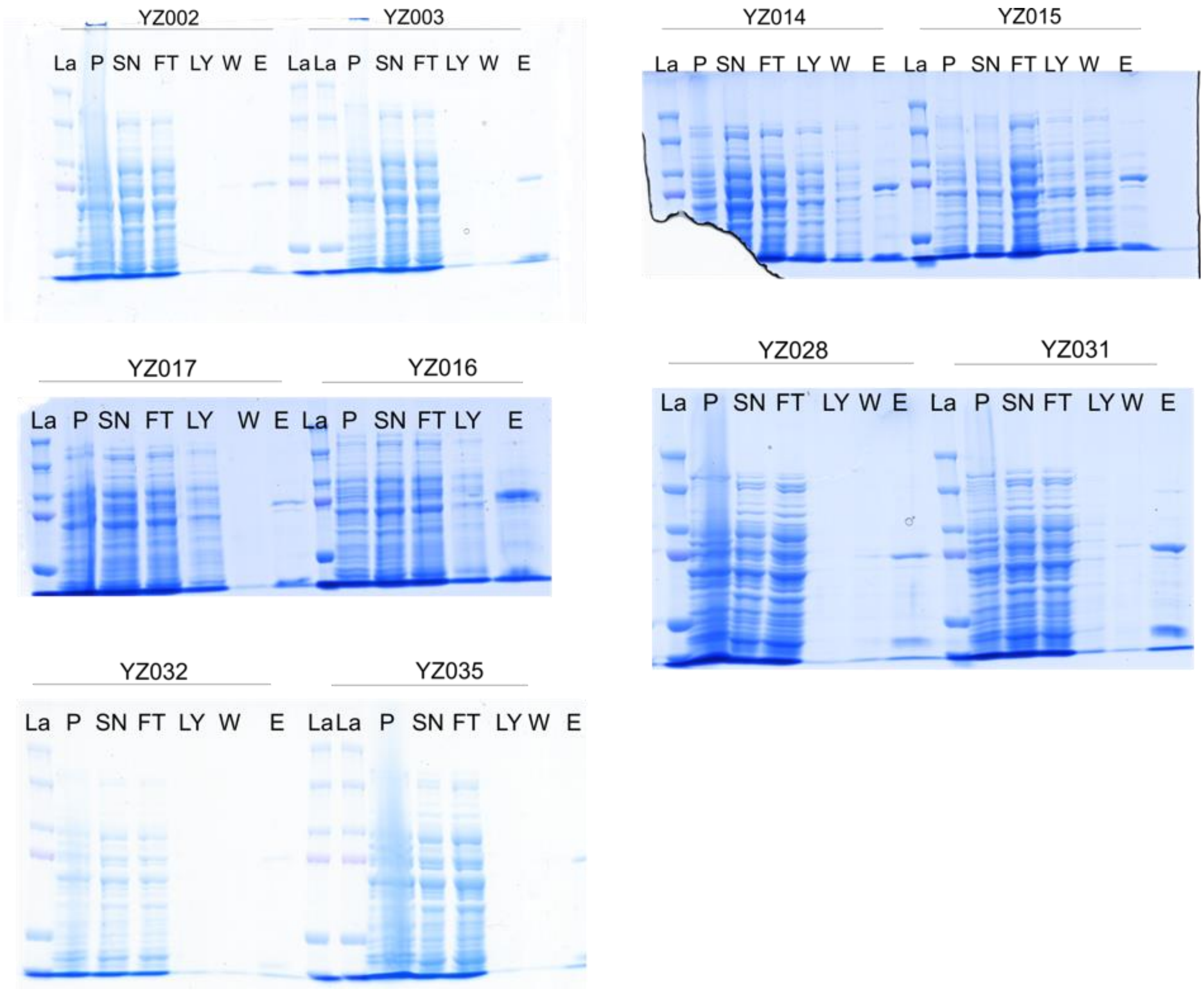

Figure S8. SDS-PAGE analysis of the test expression of MAT-swapped VEMS M2*-TE constructs via nickel affinity chromatography (part 2). The molecular weight of M2-TE constructs is around 145 kDa. Abbreviations are as follows: La – ladder, P – Pellet, FT – Flowthrough, Ly – Lysis. W – Wash, E – Elution. The Precision Plus Protein Dual Color Standard from Bio-Rad was used. Only few constructs show a slight band at the correct molecular weight in the elution fraction.

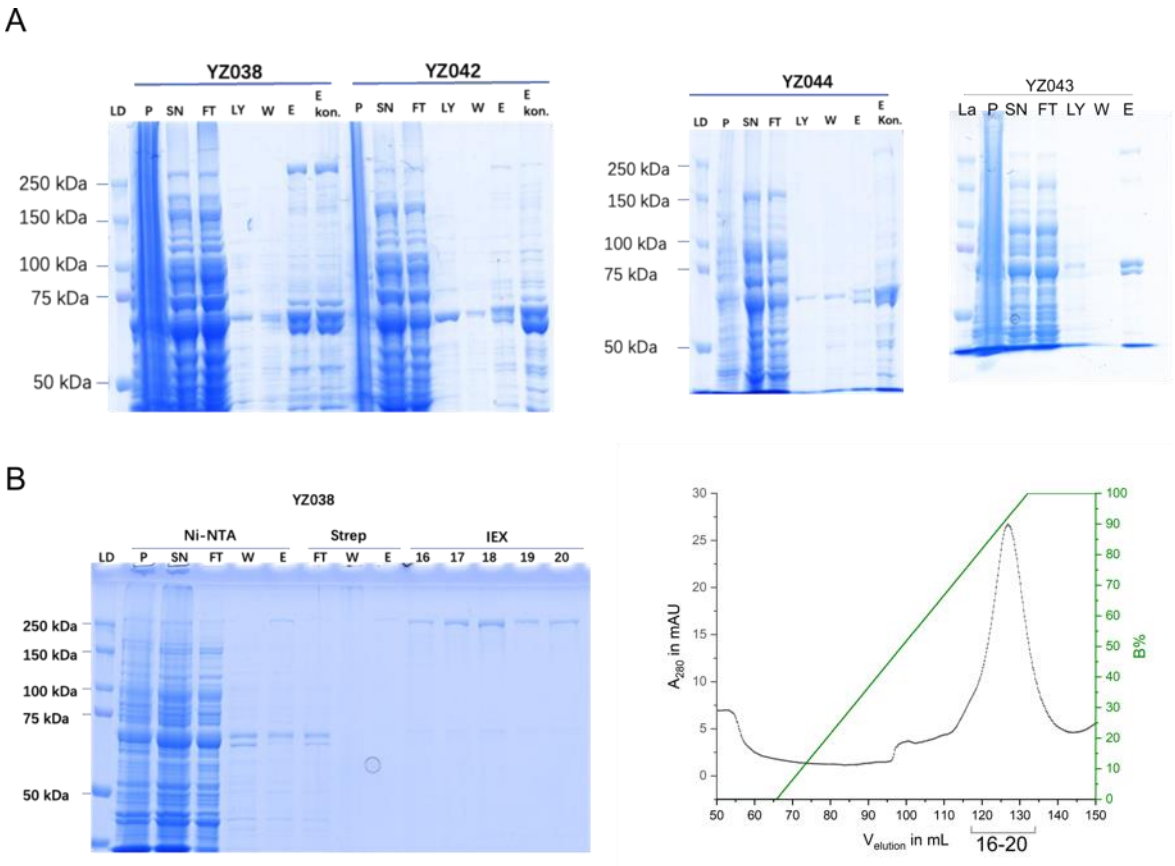

Figure S9. SDS-PAGE analysis of the test expression of MAT-swapped VEMS M0-M1* constructs via nickel affinity chromatography. (A) The molecular weights of M0-M1* constructs are around 240 kDa. Abbreviations are as follows: La or LD – ladder, P – Pellet, FT – Flowthrough, Ly – Lysis. W – Wash, E – Elution. The Precision Plus Protein Dual Color Standard from Bio-Rad was used. One construct of the H1 group (YZ038) shows a strong band at the correct molecular weight in the elution fraction. (B) YZ038 were then expressed in larger scale and purified via tandem affinity chromatography using the N-terminal Strep- and C-terminal His-tag and subsequently subjected to IEX.

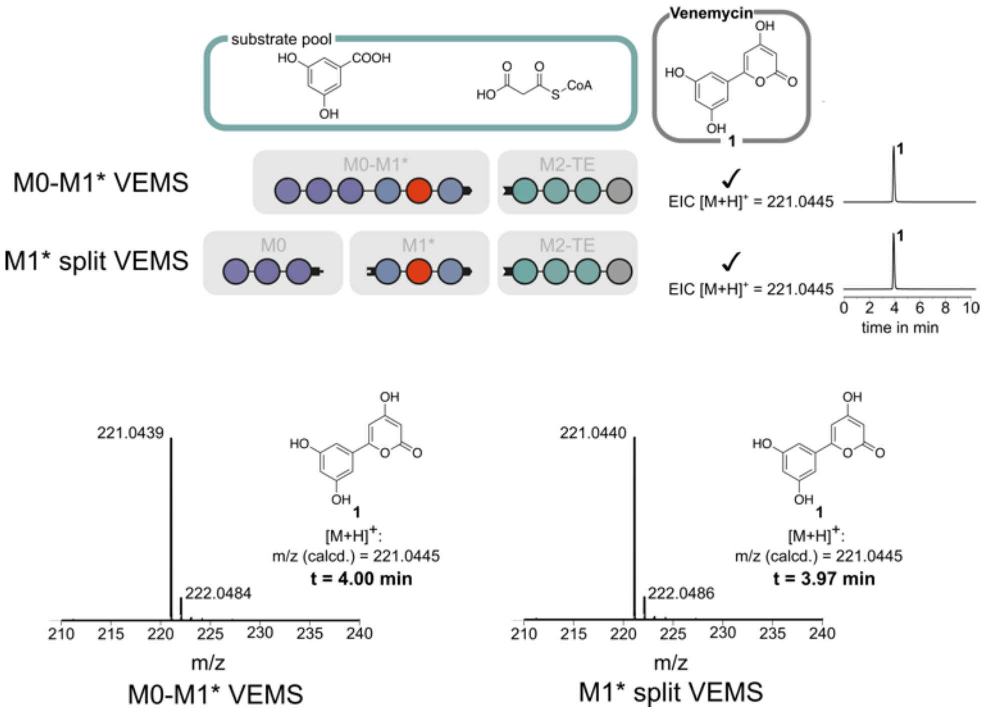

Figure S10. M1* in VEMS and split VEMS. M1* can be operated in the context of VEMS and split VEMS. The resulting assembly lines are able to produce the native VEMS product venemycin **1** when providing the appropriate substrates 3,5-dihydroxybenzoic acid (DHBA) and malonyl-CoA (MalCoA). Extracted ion chromatograms (EICs) of venemycin **1** $[M+H]^+$: m/z (calcd.) = 221.0445 produced by MAT-swapped VEMS and split VEMS. Mass spectra of **1** produced by M0-M1* VEMS and M1 split VEMS assembly lines. Liquid chromatography-high resolution mass spectrometry-electron spray ionization (HRMS-ESI): **1** $[M+H]^+$ m/z (calcd.): 221.0445; (found): 221.0440; deviation: -2.3 ppm.

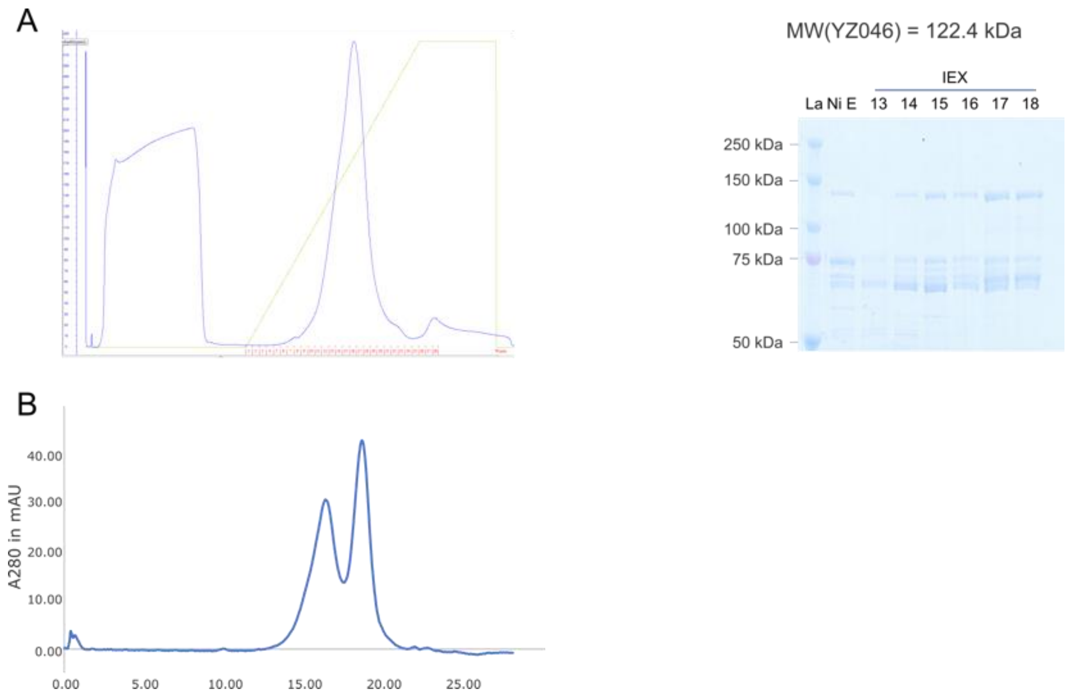

Figure S11. SDS-PAGE analysis of the test expression of MAT-swapped M1*(YZ046) via ion exchange and size exclusion chromatography. (A) The molecular weight of M1* is around 122 kDa. Abbreviations are as follows: La – ladder, Ni E – Elution from nickel column, IEX - ion exchange chromatography. The Precision Plus Protein Dual Color Standard from Bio-Rad was used. SDS PAGE reveals, that the impurities around 70 kDa cannot be removed via IEX. Fractions 13-18 were pooled and subjected to SEC. (B) SEC of YZ046 revealed two peaks. Fractions of the first peak were pooled and used in the product formation assays. Further analysis was avoided due to the low protein yield.

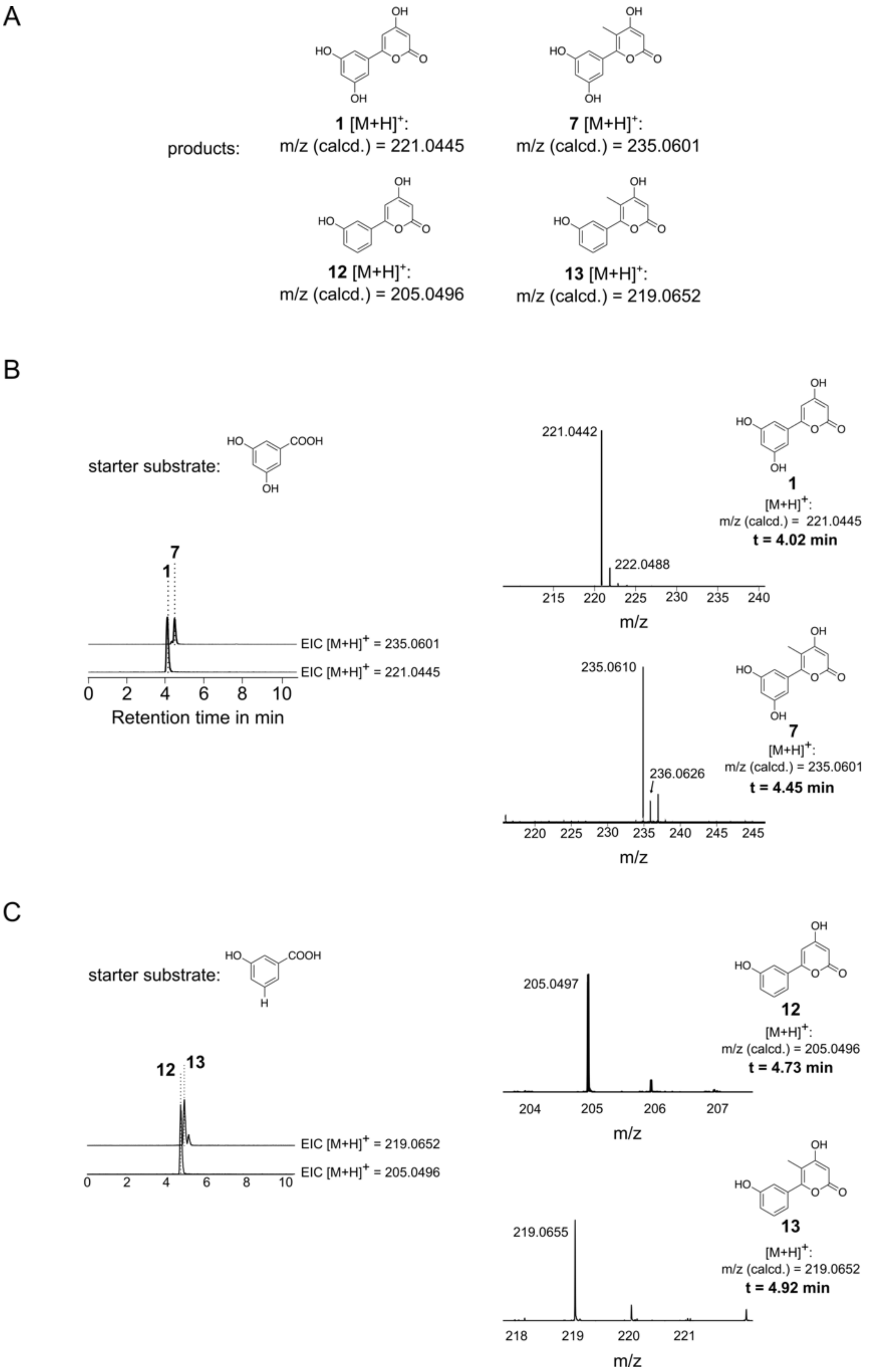
A
products:
1 [M+H]+:
m/z (calcd.) = 221.0445
7 [M+H]+:
m/z (calcd.) = 235.0601
12 [M+H]+:
m/z (calcd.) = 205.0496
13 [M+H]+:
m/z (calcd.) = 219.0652
B
starter substrate:
EIC [M+H]+ = 235.0601
EIC [M+H]+ = 221.0445
Retention time in min
221.0442
222.0488
m/z
1
[M+H]+:
m/z (calcd.) = 221.0445
t = 4.02 min
235.0610
236.0626
7
[M+H]+:
m/z (calcd.) = 235.0601
t = 4.45 min
C
starter substrate:
EIC [M+H]+ = 219.0652
EIC [M+H]+ = 205.0496
205.0497
12
[M+H]+:
m/z (calcd.) = 205.0496
t = 4.73 min
219.0655
13
[M+H]+:
m/z (calcd.) = 219.0652
t = 4.92 min

Figure S12. Products and possible side products for the reactions of M1* split VEMS shown in Figure 6 with the starter substrates DHBA and 3-hydroxybenzoic acid (HBA). (A) Possible products and their calculated m/z when subjecting different starter substrates and a MalCoA/methylmalonyl-CoA (MMalCoA) extender substrate mixture to M1* split VEMS. (B) EICs and mass spectra of the reaction mixture using DHBA. Both, compound **1** and the methylated derivative **7** could be found via LC-HRMS: **1** $[M+H]^+$ m/z (calcd.): 221.0445; (found): 221.0442; deviation: -1.4 ppm, **7** $[M+H]^+$ m/z (calcd.): 235.0601; (found): 235.0610; deviation: 3.8 ppm. (C) EICs and mass spectra of the reaction mixture using DHA. Both, compound **12** and the methylated derivative **13** could be found via LC-HRMS: **12** $[M+H]^+$ m/z (calcd.): 205.0496; (found): 205.497; deviation: 0.5 ppm, **13** $[M+H]^+$ m/z (calcd.): 219.0652; (found): 219.0655; deviation: 1.4 ppm.

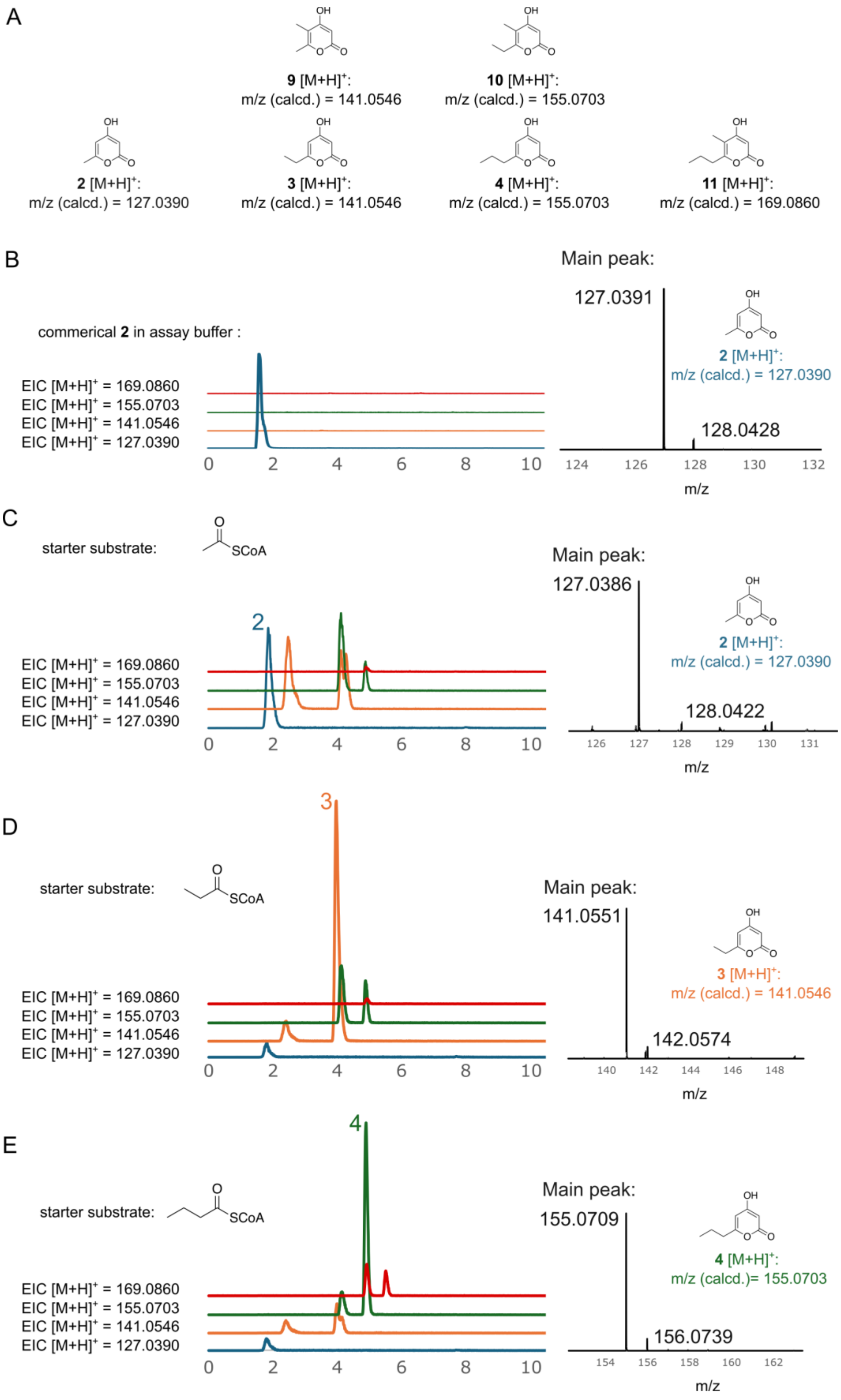
A
9 [M+H]+:
m/z (calcd.) = 141.0546
10 [M+H]+:
m/z (calcd.) = 155.0703
2 [M+H]+:
m/z (calcd.) = 127.0390
3 [M+H]+:
m/z (calcd.) = 141.0546
4 [M+H]+:
m/z (calcd.) = 155.0703
11 [M+H]+:
m/z (calcd.) = 169.0860
B
commerical 2 in assay buffer :
EIC [M+H]+ = 169.0860
EIC [M+H]+ = 155.0703
EIC [M+H]+ = 141.0546
EIC [M+H]+ = 127.0390
Main peak:
127.0391
128.0428
2 [M+H]+:
m/z (calcd.) = 127.0390
m/z
C
starter substrate:
SCoA
2
EIC [M+H]+ = 169.0860
EIC [M+H]+ = 155.0703
EIC [M+H]+ = 141.0546
EIC [M+H]+ = 127.0390
Main peak:
127.0386
128.0422
2 [M+H]+:
m/z (calcd.) = 127.0390
m/z
D
starter substrate:
SCoA
3
EIC [M+H]+ = 169.0860
EIC [M+H]+ = 155.0703
EIC [M+H]+ = 141.0546
EIC [M+H]+ = 127.0390
Main peak:
141.0551
142.0574
3 [M+H]+:
m/z (calcd.) = 141.0546
m/z
E
starter substrate:
SCoA
4
EIC [M+H]+ = 169.0860
EIC [M+H]+ = 155.0703
EIC [M+H]+ = 141.0546
EIC [M+H]+ = 127.0390
Main peak:
155.0709
156.0739
4 [M+H]+:
m/z (calcd.)= 155.0703
m/z

Figure S13. Products and possible side products for the reactions of M1* split VEMS shown in Figure 6 with the starter substrates acetyl-, propionyl-, and butyryl-CoA. (A) Possible products and their calculated m/z when subjecting different starter substrates and a MalCoA/MMalCoA extender substrate mixture to M1* split VEMS. The main products differ between the reaction mixtures and correlate to the used starter substrates. All reaction mixtures show side product formation. As reactions were run overnight and extender substrates were used in excess, side product formation is likely due to in situ decarboxylation of MalCoA and MMalCoA. By this mechanism, the compounds **2**, **3**, **9**, and **10** can be expected in each reaction mixture in similar quantities. A peak is increased if, in addition to this background production, the compound is also directly generated by usage of the provided starter substrate. (B) Analysis of commercial TAL (**2**) in assay buffer (EIC and mass spectra). LC-HRMS: **2** $[M+H]^+$ m/z (calcd.): 127.0390; (found): 127.0391; deviation: 0.8 ppm. (C) EICs of the reaction mixture using acetyl-CoA. Mass spectra of main product **2**. LC-HRMS: **2** $[M+H]^+$ m/z (calcd.): 127.0390; (found): 127.0386; deviation: -3.1 ppm. (D) EICs of the reaction mixture using propionyl-CoA. Mass spectra of main product **3**. LC-HRMS: **3** $[M+H]^+$ m/z (calcd.): 141.0546; (found): 141.0551; deviation: 3.5 ppm. (E) EICs of the reaction mixture using butyryl-CoA. Mass spectra of main product **4**. LC-HRMS: **4** $[M+H]^+$ m/z (calcd.): 155.0703; (found): 155.0709; deviation: 3.9 ppm. Of note, only single peaks occurred when running the assay without MMalCoA, which further supports side product formation by decarboxylation (s. Figure 3).

```
DEBS3 M6     VELAEAVSPWPPAAD-GVRRAGVSAFGVSGTNAHVIIAEPPEPEPLPEPGPVGVLAAANS
DEBS3 M6*    VELAEAVSPWPPAAD-GVRRAGVSAFGVSGTNAHVIIAEPPEPEPLPEPGPVGVLAAANS
PIKS M5      VELLTEAVDWPEKQDGGLRRAAVSSFGISGTNAHVVLEEAPAVEDSPAVEP---PAGGGV
PIKS M5*     VELLTEAVDWPEKQDGGLRRAAVSSFGISGTNAHVVLEEAPAVEDSPAVEP---PAGGGV
DEBS3 M5     VSVVSQARSWPAGE--RPRRAGVSSFGISGTNAHVIVEEAPEADEP---EP---APDSGP
DEBS3 M5*    VSVVSQARSWPAGE--RPRRAGVSSFGISGTNAHVIVEEAPEADEP---EP---APDSGP

DEBS3 M6     VPVLLSARTETALAAQARLLESAVDDS--VPLTALASALATGRAHLPRRAALLAGDHEQL
DEBS3 M6*    VPVLLSARTETALAAQARLLESAVDDS--VPLTALASALATGRAHLPRRAALLAGDHEQL
PIKS M5      VPWPVSAKTPAALDAQIGQLAAYADGRTDVDPAVAARALVDSRTAMEHRAVAVGDSREAL
PIKS M5*     VPWPVSAKTPAALDAQIGQLAAYADGRTDVDPAVAARALVDSRTAMEHRAVAVGDSREAL
DEBS3 M5     VPLVLSGRDEQAMRAQAGRLADHLAREPRNSLRDTGFTLATRRSAWEHRAVVVGDRDDAL
DEBS3 M5*    VPLVLSGRDEQAMRAQAGRLADHLAREPRNSLRDTGFTLATRRSAWEHRAVVVGDRDDAL

                                    Start MAT domain
DEBS3 M6     RGQLRAVAEGVAAPGATTGTASAGG-VVFVFPGQGAQWEGMARGLLSVPV-FAESIAECD
DEBS3 M6*    RGQLRAVAEGVAAPGATTGTASNKRPLWFICSGMGTQWRGMGLSLMRLDS-FRESILRSD
PIKS M5      RDALRMPEGLVR------GTSSDVGRVAFVFPGQGTQWAGMGAELLDSSPEFAASMAECE
PIKS M5*     RDALRMPEGLVR------GTSSNKRPLWFICSGMGTQWRGMGLSLMRLDS-FRESILRSD
DEBS3 M5     AGLRAVADGRIADRTATGQARTRR-GVAMVFPGQGAQWQGMARDLLRESQVFADSIRDCE
DEBS3 M5*    AGLRAVADGRIADRTATGQARTNKRPLWFICSGMGTQWRGMGLSLMRLDSFR-ESILRSD

DEBS3 M6     AVLSEVAGFSASEVLEQRPDAPSLERVDVVQPVLFSVMVSLARLWGACGVSPSAVIGHSQ
DEBS3 M6*    EAVKPLG-VKVSDLLLST-DERTFDDIVHAFVSLTAIQIALIDLLTSVGLKPDGIIGHSL
PIKS M5      TALSRYVDWSLEAVVRQEPGAPTLDRVDVVQPVTFAVMVSLAKVWQHHGITPQAVVGHSQ
PIKS M5*     EAVKPLG-VKVSDLLL-STDERTFDDIVHAFVSLTAIQIALIDLLTSVGLKPDGIIGHSL
DEBS3 M5     RALAPHVDWSLTDLL---SGARPLDRVDVVQPALFAVMVSLAALWRSHGVEPAAVVGHSQ
DEBS3 M5*    EAVKPL-GVKVSDLLL-STDERTFDDIVHAFVSLTAIQIALIDLLTSVGLKPDGIIGHSL

DEBS3 M6     GEIAAAVVAGVLSLEDGVRVVALRAKALRALAGKGGMVSLAAPGERARALIAPWEDRI--
DEBS3 M6*    GEVACGYADGCLSQREAVLAAYWRGQCIKDAHL---------PPGSMAAVGLSWEECKQR
PIKS M5      GEIAAAYVAGALTLDDAARVVTLRSKSIAAHLA---------GKGGMISLALDEAAVLKR
PIKS M5*     GEVACGYADGCLSQREAVLAAYWRGQCIKDAHL---------PPGSMAAVGLSWEECKQR
DEBS3 M5     GEIAAAHVAGALTLEDAAKLVAVRSRVLRR-LG---------GQGGMASFGLGTEQAAER
DEBS3 M5*    GEVACGYADGCLSQREAVLAAYWRGQCIKDAHL---------PPGSMAAVGLSWEECKQR

DEBS3 M6     --------SVAAVNSPSSVVVSGDPEALAELVARCEDEGVRAKTLPVD-YASHSRHVEEI
DEBS3 M6*    C---PAGVVPACHNSEDTVTISGPQAAVNEFVEQLKQEGVFAKEVRTGGLAFHSYFMEGI
PIKS M5      LSDFD-GLSVAAVNGPTATVVSGDPTQIEELARTCEADGVRARIIPV-DYASHSRQVEII
PIKS M5*     C--PA-GVVPACHNSEDTVTISGPQAAVNEFVEQLKQEGVFAKEVRTGGLAFHSYFMEGI
DEBS3 M5     IGRFAGALSIASVNGPRSVVVAGESGPLDELIAECEAEGITARRIPVD-YASHSPQVESL
DEBS3 M5*    C---PAGVVPACHNSEDTVTISGPQAAVNEFVEQLKQEGVFAKEVRTGGLAFHSYFMEGI
```

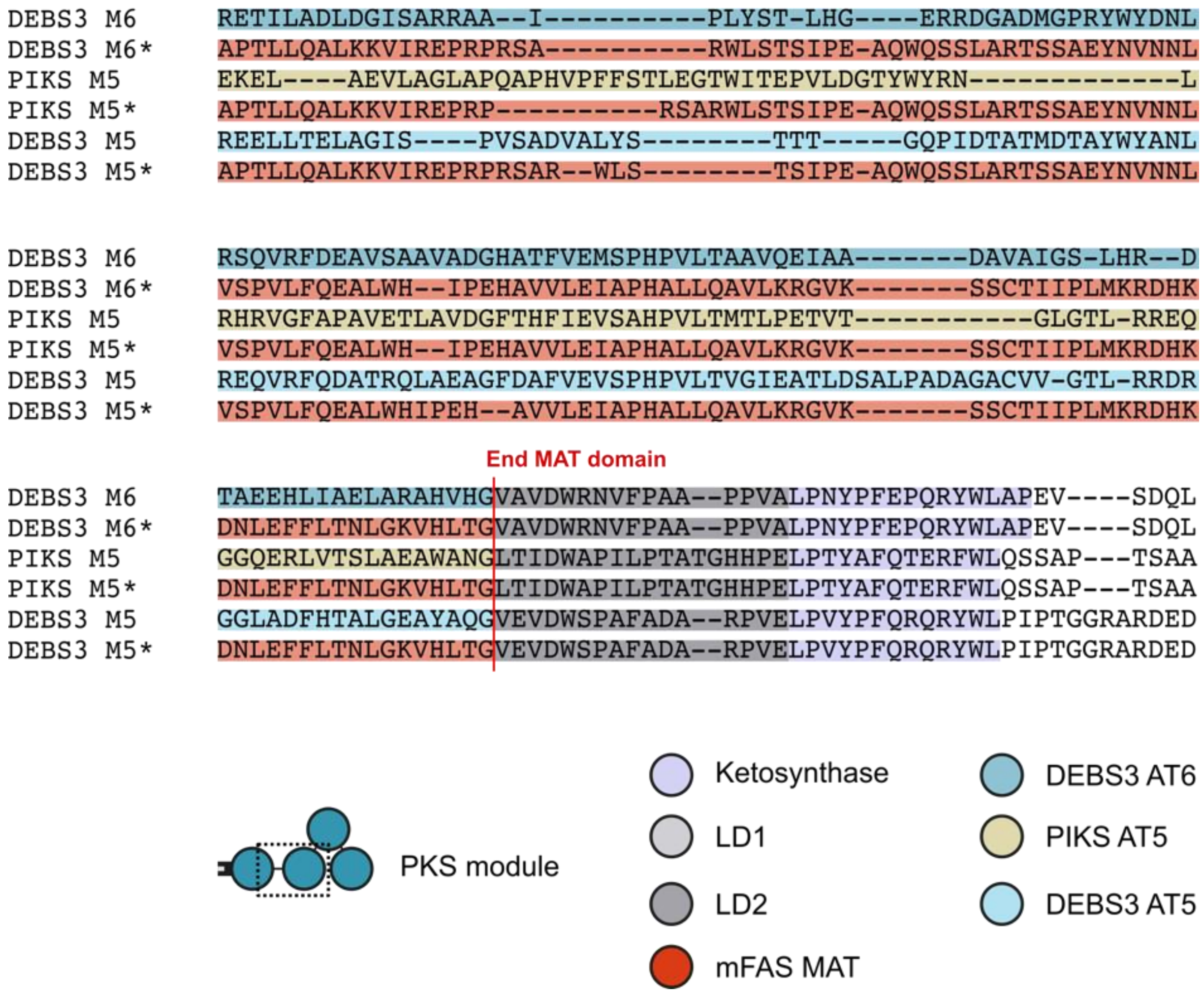

Figure S14. Design of mono-modular PikAIII- and bimodular DEBS3-based PKS/FAS hybrids. Sequence alignment of PKS modules: DEBS3 M5, DEBS3 M6 and PikAIII (M5). The respective LD_1-AT-LD_2 sequence is shown and colored according to the color code. The new PKS/FAS hybrids were designed based on the boundaries for DEBS3 M6 established by Rittner et al.[3] and verified by our hybrid design screening of VEMS M1 and VEMS M2-TE (Figures S5 and S6). The native AT domain of DEBS3 M5, DEBS3 M6 and PikAIII was swapped for the MAT domain while preserving the PKS-derived LD_1 and LD_2 sequences.

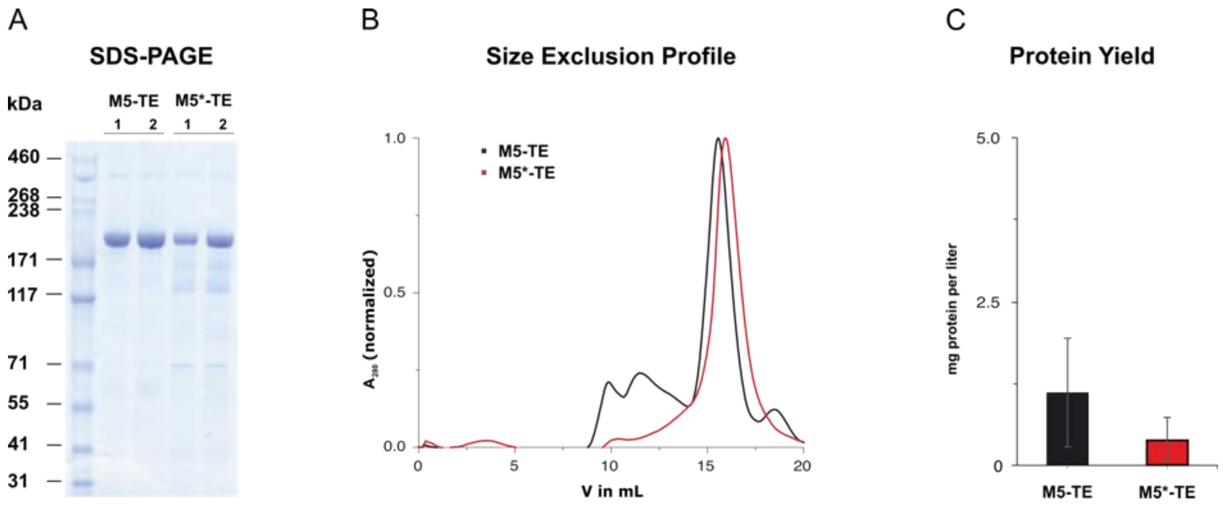

Figure S15. Purification and quality control of the mono-modular PIKS M5-TE (black) and hybrid PIKS M5 H1-TE (red). (A) SDS-PAGE of purified protein samples after SEC (NuPAGE 3-8% Tris-Acetate, Thermo Fisher). (B) SEC profiles of mono-modular constructs with absorbance normalized to highest peak. Chromatograms indicate that oligomeric state of the hybrid M5*-TE is similar to the WT. (C) Comparison of protein yield after purification of PIKS M5-TE and hybrid PIKS M5*-TE (n = 3). While high quality PIKS M5-TE could be obtained from Ni-NTA & SEC polishing while PIKS M5*-TE required an additional purification step via StrepTactinXT resin to remove impurities.

A

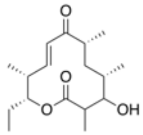

14
t = 7.46 min

	m/z (calcd.)	m/z (found)
$[M-2H_2O+H]^+$	261.1849	261.1849
$[M-H_2O+H]^+$	279.1955	279.1953
$[M+H]^+$	297.2060	297.2060
$[M+Na]^+$	319.1880	319.1880

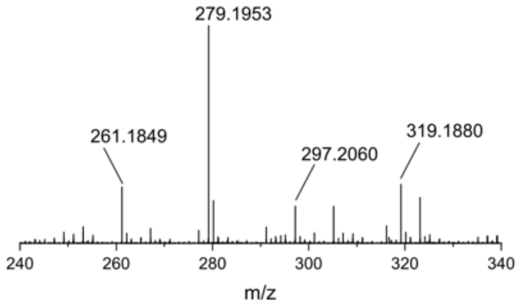

B

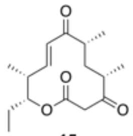

15
t = 6.70 min

	m/z (calcd.)	m/z (found)
$[M-2H_2O+H]^+$	245.1536	245.1537
$[M-H_2O+H]^+$	263.1642	263.1642
$[M+H]^+$	281.1747	281.1748
$[M+Na]^+$	303.1567	303.1567

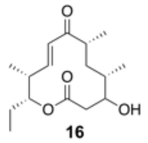

16
t = 6.80 min

	m/z (calcd.)	m/z (found)
$[M-2H_2O+H]^+$	247.1693	247.1697
$[M-H_2O+H]^+$	265.1798	265.1788
$[M+H]^+$	283.1904	283.1928
$[M+Na]^+$	305.1723	305.1732

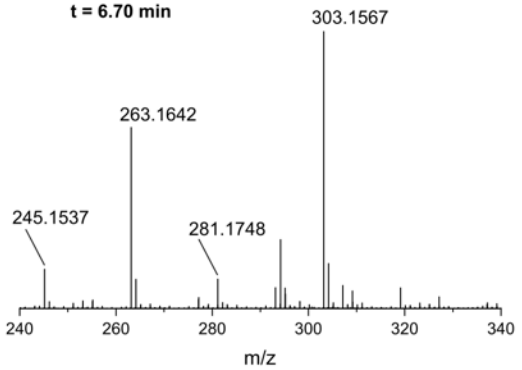

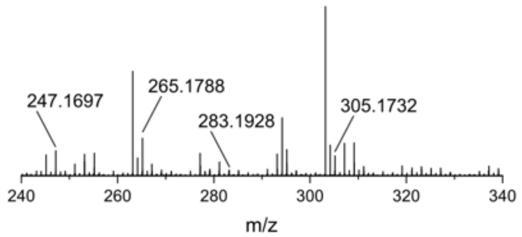

C

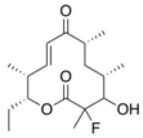

17
t = 7.44 min

	m/z (calcd.)	m/z (found)
$[M-2H_2O+H]^+$	279.1755	-
$[M-H_2O+H]^+$	297.1860	297.1858
$[M+H]^+$	315.1966	315.1964
$[M+Na]^+$	337.1786	337.1786

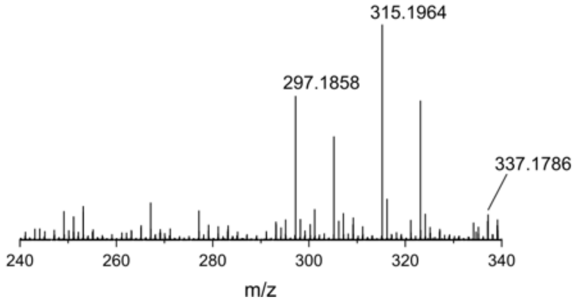

Figure S16. Mass spectra of 10-dml and its derivatives detected by LC-HRMS. C12-membered macrolactone scaffolds were produced by mono-modular PIKS M5-TE and FAS/PKS hybrid PIKS M5*-TE using PIKS pentaketide and various extender substrates (MMalCoA, MalCoA and FMMalCoA). Normalized EICs of the reaction mixtures are shown in Figure 7B and corresponding mass spectra are depicted above. Found mass corresponds to the calculated mass (m/z tolerance: ±0.003) listed for each compound. (A) Mass spectra of 10-dml **14**. LC-HRMS: **14** $[M-2H_2O+H]^+$ m/z (calcd.): 261.1849; (found): 261.1849; deviation: 0.0 ppm, $[M-H_2O+H]^+$ m/z (calcd.): 279.1955; (found): 279.1953; deviation: 0.8 ppm, $[M+H]^+$ m/z (calcd.): 297.2060; (found): 297.2060; deviation: 0.0 ppm, $[M+Na]^+$ m/z (calcd.): 319.1880; (found): 319.1880; deviation: 0.0 ppm. (B) Mass spectra of 2-demethyl-3-oxo-10-dml **15** and 2-demethyl-10-dml **16**. LC-HRMS: **15** $[M-2H_2O+H]^+$ m/z (calcd.): 245.1536; (found): 245.1537; deviation: 0.4 ppm, $[M-H_2O+H]^+$ m/z (calcd.): 263.1642; (found): 263.1642; deviation: 0.0 ppm, $[M+H]^+$ m/z (calcd.): 281.1747; (found): 281.1748; deviation: 0.4 ppm, $[M+Na]^+$ m/z (calcd.): 303.1567; (found): 303.1567; deviation: 0.0 ppm. LC-HRMS: **16** $[M-2H_2O+H]^+$ m/z (calcd.): 247.1693; (found): 247.1697; deviation: 1.6 ppm, $[M-H_2O+H]^+$ m/z (calcd.): 265,1798; (found): 265,1788; deviation:-3.8 ppm, $[M+H]^+$ m/z (calcd.): 283,1904; (found): 283.1924; deviation: 7.1 ppm, $[M+Na]^+$ m/z (calcd.): 305.1723; (found): 305.1732; deviation: 2.9 ppm. (C) Mass spectra of 2-fluoro-10-dml **17**. LC-HRMS: **17** $[M-H_2O+H]^+$ m/z (calcd.): 297.1860; (found): 297.1858; deviation: -0.7 ppm, $[M+H]^+$ m/z (calcd.): 315.1966; (found): 315.1964; deviation: -0.6 ppm, $[M+Na]^+$ m/z (calcd.): 337.1786; (found): 337.1786; deviation: 0.0 ppm. Additionally, formation of C12-membered macrolactone byproducts were observed during formation of C14-membered macrolactones from PIKS pentaketide and extender substrates mixtures by bi-modular M5-M6-TE hybrid system. Corresponding normalized EICs are shown in Figures S18D and S19B.

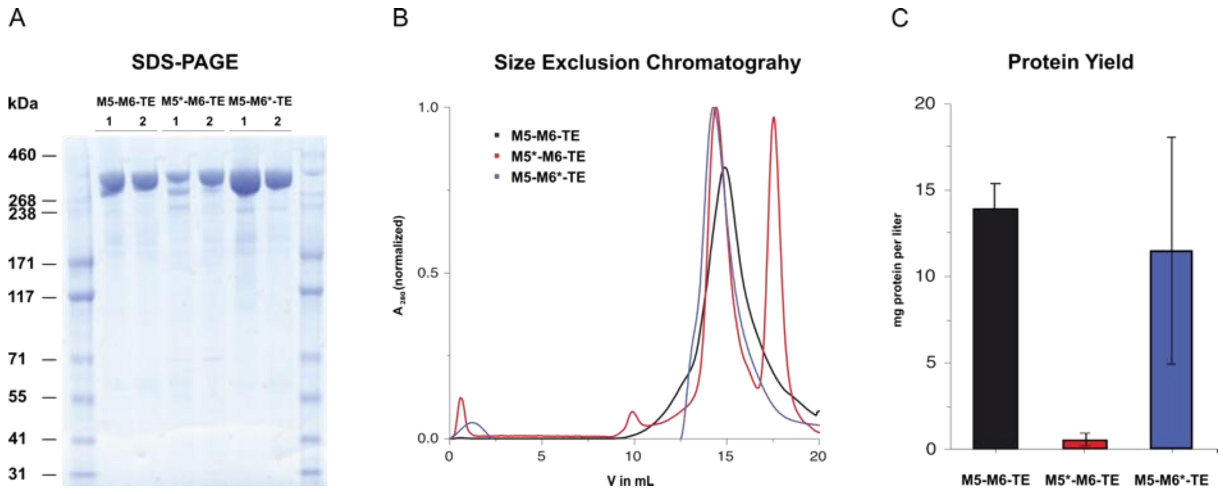

Figure S17. Purification and quality control of bimodular M5-M6-TE (black) and hybrids M5*-M6-TE (red) and M5-M6*-TE (blue). (A) SDS-PAGE of purified protein samples after SEC (NuPAGE 3-8% Tris-Acetate, Thermo Fisher) (n = 2). (B) SEC profiles of bimodular constructs with absorbance normalized to highest peak. Chromatograms indicate that oligomeric state of both hybrids is similar to the wild type (WT). All proteins elute at approx. 15 mL and smaller protein fractions are efficiently removed. (C) Comparison of protein yield after purification of M5-M6-TE and hybrids M5*-M6-TE and M5-M6*-TE (n = 3). M5-M6*-TE was obtained in very good yield and high protein quality from Ni-NTA and subsequent SEC polishing whereas the M5*-M6-TE hybrid suffered from very poor protein yield and required an additional purification step via StrepTactinXT resin.

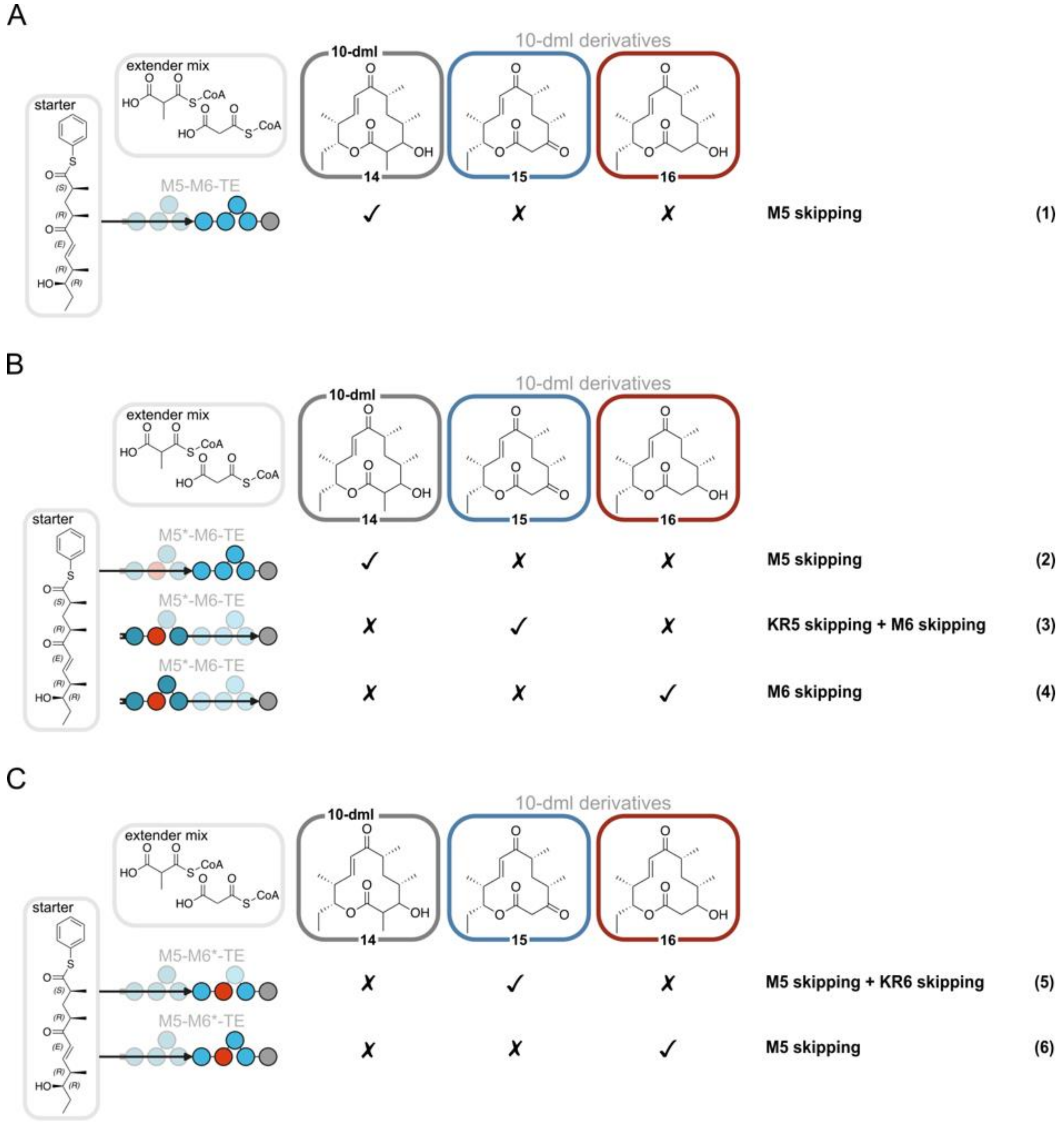
A
B
C
starter
extender mix
CoA
HO
10-dml
10-dml derivatives
14
15
16
M5-M6-TE
M5*-M6-TE
M5-M6*-TE
M5 skipping (1)
M5 skipping (2)
KR5 skipping + M6 skipping (3)
M6 skipping (4)
M5 skipping + KR6 skipping (5)
M5 skipping (6)

D

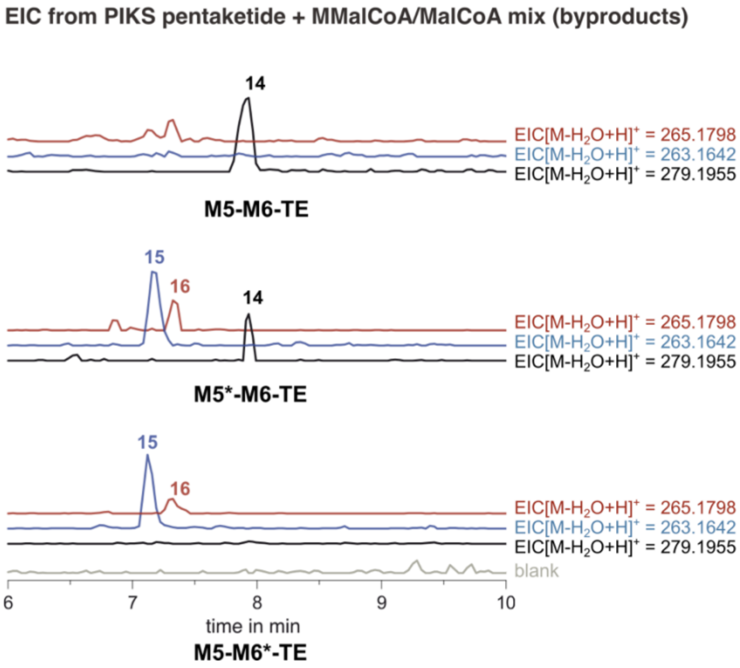

Figure S18. Proposed reaction pathway to produce 10-dml byproducts from MMalCoA/MalCoA mixtures. The complex product mixtures obtained from DEBS3-mediated macrolactone preparation include the 3-oxo-narbonolide derivatives **18**, **19** and **21** as well as several 10-dml-based byproducts **14** – **16**. We conclude that several alternative reaction pathways account for the formation of compounds **14** – **16**. (A) For M5-M6-TE, formation of native 10-dml **14** is attributed to "module skipping" (of M5) and direct loading of the KS domain in M6. It was previously shown that PIKS pentaketide can be directly loaded to M6 for one step of chain elongation followed by TE-mediated off-loading via DEBS TE[3]. (No other 10-dml-based byproducts were identified demonstrating the substrate specificity of PKS-derived AT domains. (B) M5*-M6-TE produces the most 10-dml-based byproducts. Formation of native 10-dml **14** was again assigned to "module skipping" of M5. De-methylated byproducts **15** and **16** are a result from MAT-mediated incorporation in hybrid module M5*. We propose that the de-methylated intermediate is then directly transferred to DEBS TE while skipping M6. Presumably, translocation of the non-native, de-methylated intermediate from ACP5 onto KS6 is impaired resulting in increased transfer to the TE domain. The presence of non-reduced de-methylated 10-dml **15** is most likely a result of "KR skipping" due to inefficient active site binding to the KR and direct transfer to the TE domain. (C) For hybrid M5-M6*-TE, de-methylated byproducts **15** and **16** are a result of M5 module skipping and direct loading of PIKS pentaketide to KS6. Hybrid module M6* is then highly efficient in installing MalCoA only. As before, "KR skipping" due to impaired intermediate binding results in formation of non-reduced product **15**. (D) EICs of 10-dml-based byproducts (**14** $[M+H-H_2O]^+$: m/z (calcd.): 279.1955, **15** $[M+H-H_2O]^+$: m/z (calcd.): 263.1642, **16** $[M+H-H_2O]^+$: m/z (calcd.): 265.1798).

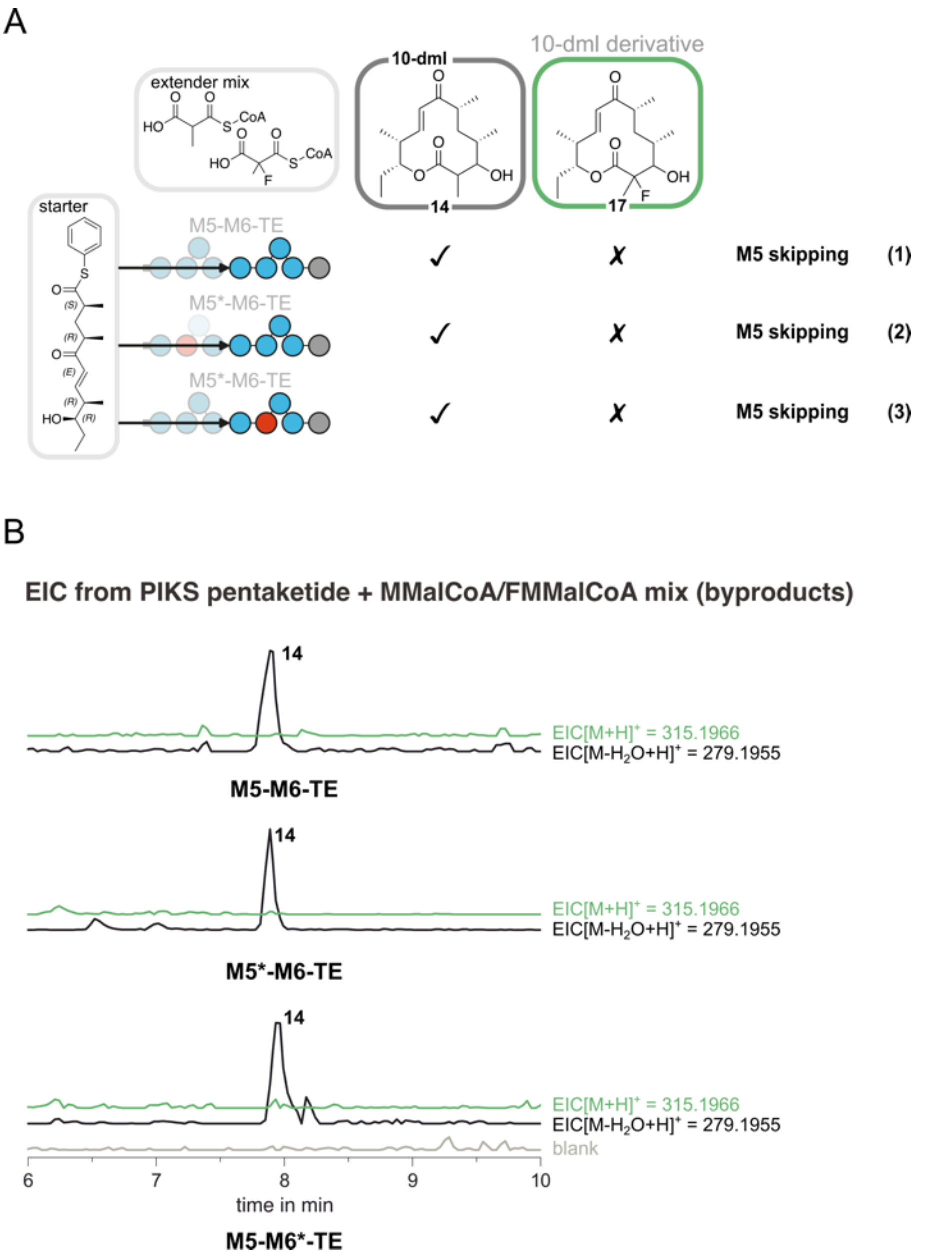

Figure S19. Proposed reaction pathway to produce 10-dml byproduct from MMalCoA/Fluoromethylmalonyl-CoA (FMMalCoA) mixtures. The complex product mixtures obtained from DEBS3-mediated macrolactone preparation include the 3-oxo-narbonolide derivatives **18**, **20** and **21** as well as 10-dml **14** as byproduct. (A) The formation of fluorinated 10-dml byproduct **17** was not observed for the bimodular systems confirming the preference of the MAT domain for MMalCoA over FMMalCoA extender substrate. For each protein, we assumed a similar alternative reaction pathway responsible for the formation of compound **14** that is module skipping of M5 and direct loading of KS6 as already suggested for assays containing mixtures of MMalCoA/MalCoA. (B) EICs of 10-dml-based byproducts (**14** $[M+H-H_2O]^+$: m/z (calcd.): 279.1955, **17** $[M+H]^+$: m/z (calcd.): 315.1966).

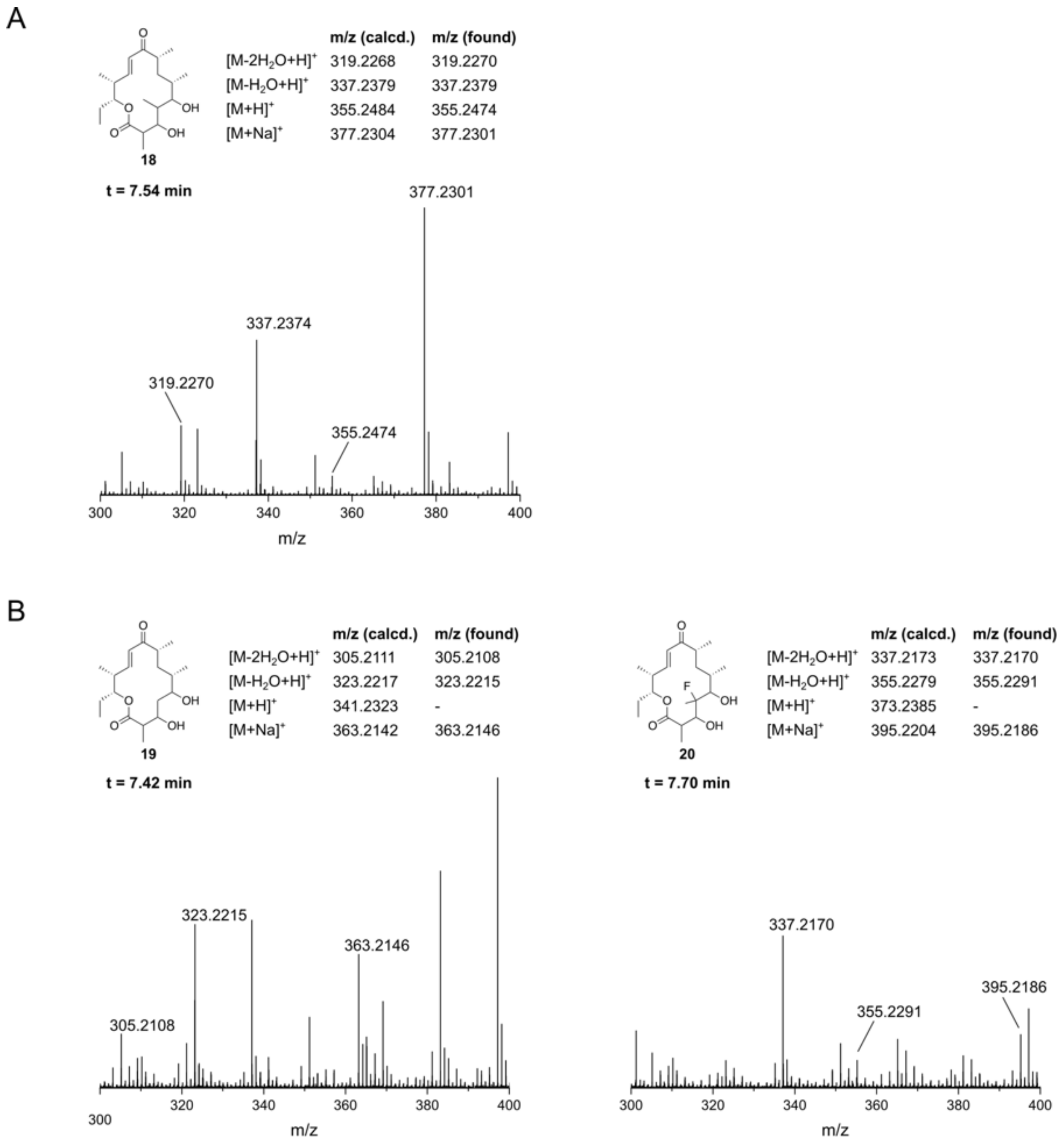

Figure S20. Mass spectra of 3-hydroxy-narbonolide and its derivatives detected by LC-HRMS. C14-membered macrolactone scaffolds were produced by bi-modular DEBS M5-M6-TE and FAS/PKS hybrids M5*-M6-TE and M5-M6*-TE using PIKS pentaketide and mixtures of extender substrates (MMalCoA/MalCoA and MMalCoA/FMMalCoA, respectively). Normalized EICs of the reaction mixtures are shown in Figure 9C (MMalCoA/MalCoA) and Figure 9D (MMalCoA/FMMalCoA) and corresponding mass spectra are summarized above. Found mass corresponds to the calculated mass listed for each compound. (A) Mass spectra of 3-hydroxy-narbonolide **18**. LC-HRMS: **18** $[M-2H_2O+H]^+$ m/z (calcd.): 319.2268; (found): 319.2270; deviation: 0.6 ppm, $[M-H_2O+H]^+$ m/z (calcd.): 337.2379; (found): 337.2379; deviation: 0.0 ppm, $[M+H]^+$ m/z (calcd.): 355.2484; (found): 355.2474; deviation: -2.8 ppm, $[M+Na]^+$ m/z (calcd.): 377.2304; (found): 377.2301; deviation: -0.8 ppm. (B) Mass spectra of 4-demethyl-3-hydroxy-narbonolide **19** and 4-fluoro-3-hydroxy-narbonolide **20**. LC-HRMS: **19** $[M-2H_2O+H]^+$ m/z (calcd.): 305.2111; (found): 305.2108; deviation: -2.0 ppm, $[M-H_2O+H]^+$ m/z (calcd.): 323.2217; (found): 323.2215; deviation: -0.6 ppm, $[M+Na]^+$ m/z (calcd.): 363.2142; (found): 363.2146; deviation: 1.1 ppm. LC-HRMS: **20** $[M-2H_2O+H]^+$ m/z (calcd.): 337.2173; (found): 337.2170; deviation: -0.9 ppm, $[M-H_2O+H]^+$ m/z (calcd.): 355.2279; (found): 355.2291; deviation: 3.4 ppm, $[M+Na]^+$ m/z (calcd.): 395.2204; (found): 395.2186; deviation: -4.6 ppm.

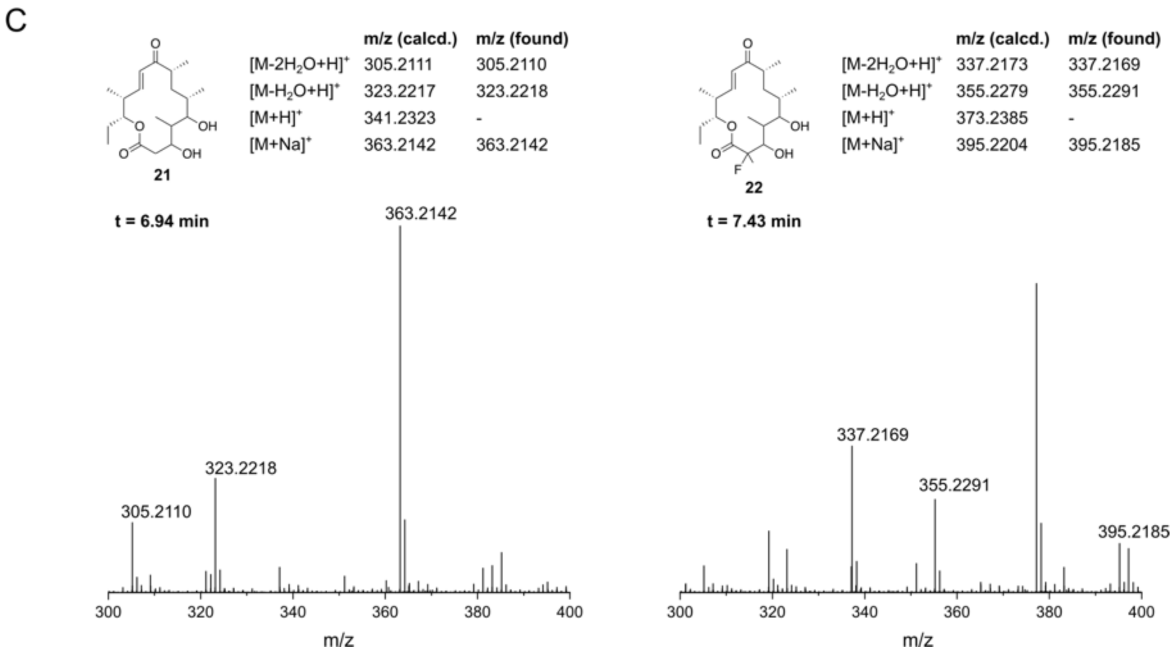

Figure S20 (continued). Mass spectra of 3-hydroxy-narbonolide and its derivatives detected by LC-HRMS. (C) Mass spectra of 2-demethyl-3-hydroxy-narbonolide **21** and 2-fluoro-3-hydroxy-narbonolide **22**. LC-HRMS: **21** $[M-2H_2O+H]^+$ m/z (calcd.): 305.2111; (found): 305.2110; deviation: -0.3 ppm, $[M-H_2O+H]^+$ m/z (calcd.): 323.2217; (found): 323.2218; deviation: 0.3 ppm, $[M+Na]^+$ m/z (calcd.): 363.2142; (found): 363.2142; deviation: 0.0 ppm. LC-HRMS: **22** $[M-2H_2O+H]^+$ m/z (calcd.): 337.2173; (found): 337.2169; deviation: -1.2 ppm, $[M-H_2O+H]^+$ m/z (calcd.): 355.2279; (found): 355.2291; deviation: 3.4 ppm, $[M+Na]^+$ m/z (calcd.): 395.2204; (found): 395.2185; deviation: -4.8 ppm.

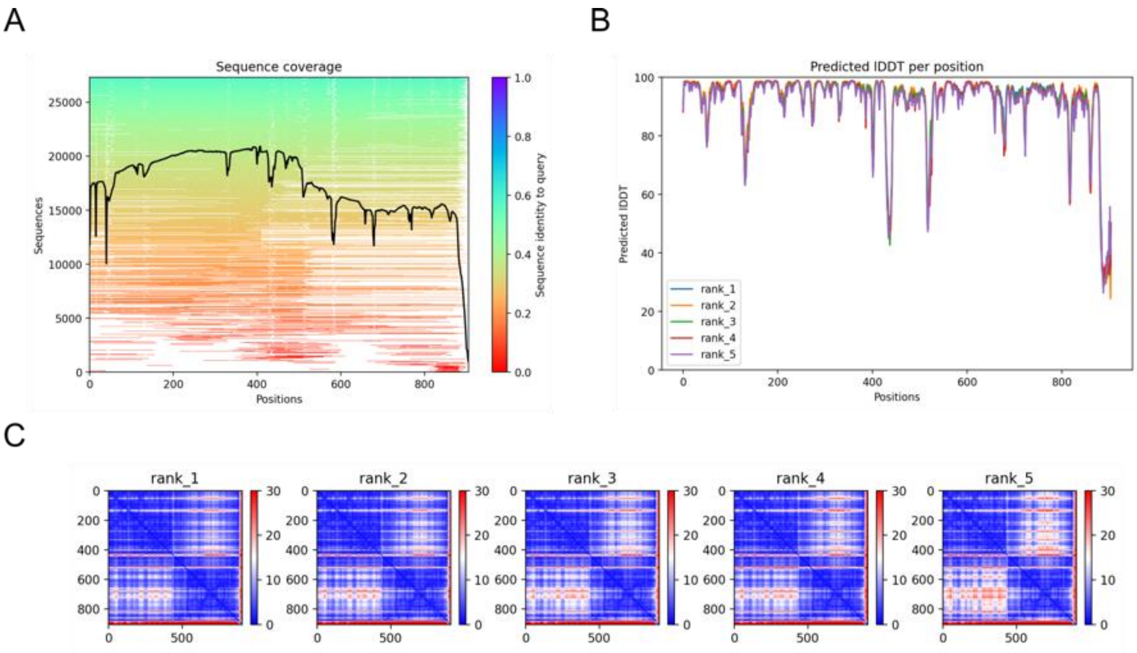

Figure S21. Quality of the VemG KS1-AT1 didomain structure predicted with ColabFold[2]. AlphaFold error estimates of the VemG KS1-AT1 didomain shown in Figures 4 and S6. The amino acid sequence used for the structural prediction is provided in Table S10. (A) Sequence Coverage. (B) Per-residue confidence estimate of predictions (pLDDT) on a scal from 0 – 100 (higher pLDDT is better). (C) Predicted aligned error (PAE) for the five predicted didomains. Only the best ranked structure (rank 1) was used for selecting the swap boundaries shown in Figure S6.

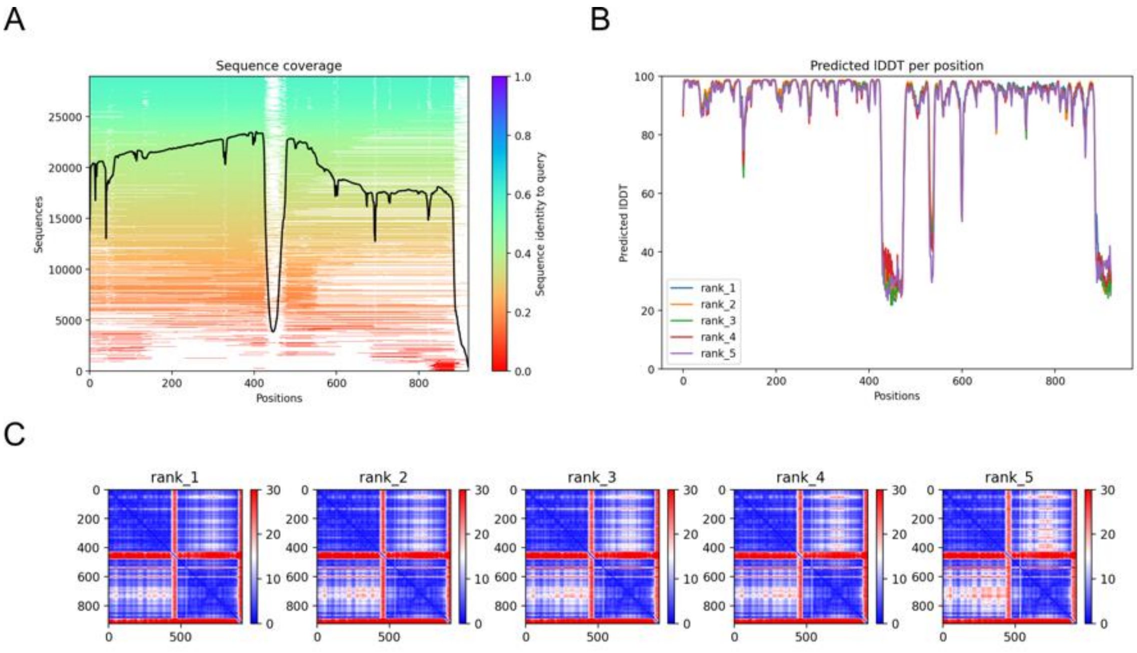

Figure S22. Quality of the VemH KS2-AT2 didomain structure predicted with ColabFold[2]. AlphaFold error estimates of the VemH KS2-AT2 didomain shown in Figure S5. The amino acid sequence used for the structural prediction is provided in Table S10. (A) Sequence Coverage. (B) Per-residue confidence estimate of predictions (pLDDT) on a scal from 0 – 100 (higher pLDDT is better). (C) Predicted aligned error (PAE) for the five predicted didomains. Only the best ranked structure (rank 1) was used for selecting the swap boundaries shown in Figure S5.

Supplementary Tables

Table S1. Cloning strategy of plasmids generated in this study. Individual fragments were generated by PCR and assembled via In-Fusion cloning. See Table S2 for template sequences.

Plasmid	Cloning Method	Fragments	Primer Name	Primer Sequence 5'-3'	Template
pLB062	In-Fusion	pLB062I	P-LB206	CATCACAGCAGCGGCTCCACCAACAAGCGCCCAC	pAR340[4]
			P-LB208	TGCGACCTCGGTTCCGGAGGACATTTCCTGAAGTTTCC	
		pLB062V	P-LB205	GCCGCTGCTGTGATGATGATG	pBL12[5]
			P-LB207	GGAACCGAGGTCGCACAAC	
pLB063	In-Fusion	pLB063I	P-MJD091	AACAAGCGCCCACTCTG	pAR340[4]
			P-LB209	AGAGCCACCTGCCGATCC	
		pLB063V	P-LB210	GAGTGGGCGCTTGTTGGATCCGGCGCCTTTTTCG	pLB047[1]
			P-LB211	TCGGCAGGTGGCTCTCTGCGGGAACGCCTCGG	
pYZ002	In-Fusion	pYZ002I	P-YZ001	ACCGGCGTCGCGCGCAAGCGCCCACTCTGGTTCATC	pAR127[6]
			P-LB235	CCAGTCGACCGTGCCGCCTGTGAGGTGCACCTTG	
		pYZ002V	P-YZ044	GCGCGCGACGCCGGTGAC	pCS005[1]
			P-LB236	GGCACGGTCGACTGGAGC	
pYZ003	In-Fusion	pYZ003I	P-YZ001	ACCGGCGTCGCGCGCAAGCGCCCACTCTGGTTCATC	pAR127[6]
			P-YZ029	GACCGTGCCGCCGTCGAGGTGCACCTTGCCAAGGTTG	
		pYZ003V	P-YZ044	GCGCGCGACGCCGGTGAC	pCS005[1]
			P-YZ030	GACGGCGGCACGGTCG	
pYZ004	In-Fusion	pYZ004I	P-YZ001	ACCGGCGTCGCGCGCAAGCGCCCACTCTGGTTCATC	pAR127[6]
			P-YZ031	GTCCACGTGGACCTGGCCAAGGTTGGTGAGAAAGAACTC	
		pYZ004V	P-YZ044	GCGCGCGACGCCGGTGAC	pCS005[1]
			P-YZ032	CAGGTCCACGTGGACGGCG	
pYZ005	In-	pYZ005I	P-YZ008	GTCGCGCGCCCCGGCCCACTCTGGTTCATCTGCTCAG	pAR127[6]

Plasmid	Cloning Method	Fragme nts	Primer Name	Primer Sequence 5'-3'	Template
	Fusion		P-LB235	CCAGTCGACCGTGCCGCCTGTGAGGTGCACCTTG	
		pYZ005V	P-YZ045	GCCGGGGCGCGCGACG	pCS005[1]
			P-LB236	GGCACGGTCGACTGGAGC	
pYZ006	In-Fusion	pYZ006I	P-YZ008	GTCGCGCGCCCCGGCCCACTCTGGTTCATCTGCTCAG	pAR127[6]
			P-YZ029	GACCGTGCCGCCGTCGAGGTGCACCTTGCCAAGGTTG	
		pYZ006V	P-YZ045	GCCGGGGCGCGCGACG	pCS005[1]
			P-YZ030	GACGGCGGCACGGTCG	
pYZ007	In-Fusion	pYZ007I	P-YZ008	GTCGCGCGCCCCGGCCCACTCTGGTTCATCTGCTCAG	pAR127[6]
			P-YZ031	GTCCACGTGGACCTGGCCAAGGTTGGTGAGAAAGAACTC	
		pYZ007V	P-YZ045	GCCGGGGCGCGCGACG	pCS005[1]
			P-YZ032	CAGGTCCACGTGGACGGCG	
pYZ008	In-Fusion	pYZ008I	P-YZ010	GCGCTGCTCTTCTCCGGGATGGGCACGCAGTGG	pAR127[6]
			P-LB235	CCAGTCGACCGTGCCGCCTGTGAGGTGCACCTTG	
		pYZ008V	P-YZ011	GGAGAAGAGCAGCGCGGTG	pCS005[1]
			P-LB236	GGCACGGTCGACTGGAGC	
pYZ009	In-Fusion	pYZ009I	P-YZ010	GCGCTGCTCTTCTCCGGGATGGGCACGCAGTGG	pAR127[32] [6]
			P-YZ029	GACCGTGCCGCCGTCGAGGTGCACCTTGCCAAGGTTG	
		pYZ009V	P-YZ011	GGAGAAGAGCAGCGCGGTG	pCS005[1]
			P-YZ030	GACGGCGGCACGGTCG	
pYZ014	In-Fusion	pYZ014I	P-YZ033	TCCGCGACCCCTCCCACTGCACACGCTGCCCTTC	pAR127[6]
			P-YZ023	CACCAGCCTGCCCCCGGGAGCCGGGAACTCCAC	
		pYZ014V	P-YZ040	GGGAGGGGTCGCGGACGG	pCS005[1]
			P-YZ024	GGGGGCAGGCTGGTGG	
pYZ015	In-Fusion	pYZ015I	P-YZ033	TCCGCGACCCCTCCCACTGCACACGCTGCCCTTC	pAR127[6]
			P-YZ025	GGGGAGGTCCACCAGAGTCCCTCGGGGAGCCG	

Plasmid	Cloning Method	Fragments	Primer Name	Primer Sequence 5'-3'	Template
		pYZ015V	P-YZ040	GGGAGGGGTCGCGGACGG	pCS005[1]
			P-YZ035	CTGGTGGACCTCCCCACGTAC	
pYZ016	In-Fusion	pYZ016I	P-YZ033	TCCGCGACCCCTCCCACTGCACACGCTGCCCTTC	pAR127[6]
			P-YZ027	GGGGTACGTGGGGAGGATGAGAGGAGTCCCTCGG	
		pYZ016V	P-YZ040	GGGAGGGGTCGCGGACGG	pCS005[1]
			P-YZ028	CTCCCCACGTACCCCTTCC	
pYZ017	In-Fusion	pYZ017I	P-YZ017	GCGCCCGGCCGCGCCGCCCTTCCCCATTTGCTGC	pAR127[6]
			P-YZ023	CACCAGCCTGCCCCCGGGAGCCGGGAACTCCAC	
		pYZ017V	P-YZ041	GGCGCGGCCGGGCGCC	pCS005[1]
			P-YZ024	GGGGGCAGGCTGGTGG	
pYZ018	In-Fusion	pYZ018I	P-YZ017	GCGCCCGGCCGCGCCGCCCTTCCCCATTTGCTGC	pAR127[6]
			P-YZ025	GGGGAGGTCCACCAGAGTCCCTCGGGGAGCCG	
		pYZ018V	P-YZ041	GGCGCGGCCGGGCGCC	pCS005[1]
			P-YZ035	CTGGTGGACCTCCCCACGTAC	
pYZ019	In-Fusion	pYZ019I	P-YZ017	GCGCCCGGCCGCGCCGCCCTTCCCCATTTGCTGC	pAR127[6]
			P-YZ027	GGGGTACGTGGGGAGGATGAGAGGAGTCCCTCGG	
		pYZ019V	P-YZ041	GGCGCGGCCGGGCGCC	pCS005[1]
			P-YZ028	CTCCCCACGTACCCCTTCC	
pYZ020	In-Fusion	pYZ020I	P-YZ019	GGCCGCGCCCGGCCGCCCCATTTGCTGCACGCCAG	pAR127[6]
			P-YZ023	CACCAGCCTGCCCCCGGGAGCCGGGAACTCCAC	
		pYZ020V	P-YZ042	CGGCCGGGCGCGGCCG	pCS005[1]
			P-YZ024	GGGGGCAGGCTGGTGG	
pYZ021	In-Fusion	pYZ021I	P-YZ019	GGCCGCGCCCGGCCGCCCCATTTGCTGCACGCCAG	pAR127[6]
			P-YZ025	GGGGAGGTCCACCAGAGTCCCTCGGGGAGCCG	
		pYZ021V	P-YZ042	CGGCCGGGCGCGGCCG	pCS005[1]

Plasmid	Cloning Method	Fragme nts	Primer Name	Primer Sequence 5'-3'	Template
			P-YZ035	CTGGTGGACCTCCCCACGTAC	
pYZ022	In-Fusion	pYZ022I	P-YZ019	GGCCGCGCCCGGCCGCCCCATTTGCTGCACGCCAG	pAR127[6]
			P-YZ027	GGGGTACGTGGGGAGGATGAGAGGAGTCCCTCGG	
		pYZ022V	P-YZ042	CGGCCGGGCGCGGCCG	pCS005[1]
			P-YZ028	CTCCCCACGTACCCCTTCC	
pYZ023	In-Fusion	pYZ023I	P-YZ021	GCCCGGCCGCCGGTGTTGCTGCACGCCAGTGGAC	pAR127[6]
			P-YZ023	CACCAGCCTGCCCCCGGGAGCCGGGAACTCCAC	
		pYZ023V	P-YZ043	CACCGGCGGCCGGGCGC	pCS005[1]
			P-YZ024	GGGGGCAGGCTGGTGG	
pYZ024	In-Fusion	pYZ024I	P-YZ021	GCCCGGCCGCCGGTGTTGCTGCACGCCAGTGGAC	pAR127[6]
			P-YZ025	GGGGAGGTCCACCAGAGTCCCTCGGGGAGCCG	
		pYZ024V	P-YZ043	CACCGGCGGCCGGGCGC	pCS005[1]
			P-YZ035	CTGGTGGACCTCCCCACGTAC	
pYZ025	In-Fusion	pYZ025I	P-YZ021	GCCCGGCCGCCGGTGTTGCTGCACGCCAGTGGAC	pAR127[6]
			P-YZ027	GGGGTACGTGGGGAGGATGAGAGGAGTCCCTCGG	
		pYZ025V	P-YZ043	CACCGGCGGCCGGGCGC	pCS005[1]
			P-YZ028	CTCCCCACGTACCCCTTCC	
pYZ026	In-Fusion	pYZ026I	P-YZ033	TCCGCGACCCCTCCCACTGCACACGCTGCCCTTC	pAR127[6]
			P-YZ031	GTCCACGTGGACCTGGCCAAGGTTGGTGAGAAAGAACTC	
		pYZ026V	P-YZ040	GGGAGGGGTCGCGGACGG	pCS005[1]
			P-YZ032	CAGGTCCACGTGGACGGCG	
pYZ027	In-Fusion	pYZ027I	P-YZ033	TCCGCGACCCCTCCCACTGCACACGCTGCCCTTC	pAR127[6]
			P-YZ029	GACCGTGCCGCCGTCGAGGTGCACCTTGCCAAGGTTG	
		pYZ027V	P-YZ040	GGGAGGGGTCGCGGACGG	pCS005[1]
			P-YZ030	GACGGCGGCACGGTCG	

Plasmid	Cloning Method	Fragme nts	Primer Name	Primer Sequence 5'-3'	Template
pYZ028	In-Fusion	pYZ028I	P-YZ033	TCCGCGACCCCTCCCACTGCACACGCTGCCCTTC	pAR127[6]
			P-LB235	CCAGTCGACCGTGCCGCCTGTGAGGTGCACCTTG	
		pYZ028V	P-YZ040	GGGAGGGGTCGCGGACGG	pCS005[1]
			P-LB236	GGCACGGTCGACTGGAGC	
pYZ029	In-Fusion	pYZ029I	P-YZ017	GCGCCCGGCCGCGCCGCCCTTCCCCATTTGCTGC	pAR127[6]
			P-YZ031	GTCCACGTGGACCTGGCCAAGGTTGGTGAGAAAGAACTC	
		pYZ029V	P-YZ041	GGCGCGGCCGGGCGCC	pCS005[1]
			P-YZ032	CAGGTCCACGTGGACGGCG	
pYZ030	In-Fusion	pYZ030I	P-YZ017	GCGCCCGGCCGCGCCGCCCTTCCCCATTTGCTGC	pAR127[6]
			P-YZ029	GACCGTGCCGCCGTCGAGGTGCACCTTGCCAAGGTTG	
		pYZ030V	P-YZ041	GGCGCGGCCGGGCGCC	pCS005[1]
			P-YZ030	GACGGCGGCACGGTCG	
pYZ031	In-Fusion	pYZ031I	P-YZ017	GCGCCCGGCCGCGCCGCCCTTCCCCATTTGCTGC	pAR127[6]
			P-LB235	CCAGTCGACCGTGCCGCCTGTGAGGTGCACCTTG	
		pYZ031V	P-YZ041	GGCGCGGCCGGGCGCC	pCS005[1]
			P-LB236	GGCACGGTCGACTGGAGC	
pYZ032	In-Fusion	pYZ032I	P-YZ019	GGCCGCGCCCGGCCGCCCCATTTGCTGCACGCCAG	pAR127[6]
			P-YZ031	GTCCACGTGGACCTGGCCAAGGTTGGTGAGAAAGAACTC	
		pYZ032V	P-YZ042	CGGCCGGGCGCGGCCG	pCS005[1]
			P-YZ032	CAGGTCCACGTGGACGGCG	
pYZ033	In-Fusion	pYZ033I	P-YZ019	GGCCGCGCCCGGCCGCCCCATTTGCTGCACGCCAG	pAR127[6]
			P-YZ029	GACCGTGCCGCCGTCGAGGTGCACCTTGCCAAGGTTG	
		pYZ033V	P-YZ042	CGGCCGGGCGCGGCCG	pCS005[1]
			P-YZ030	GACGGCGGCACGGTCG	
pYZ034	In-	pYZ034I	P-YZ019	GGCCGCGCCCGGCCGCCCCATTTGCTGCACGCCAG	pAR127[6]

Plasmid	Cloning Method	Fragments	Primer Name	Primer Sequence 5'-3'	Template
	Fusion		P-LB235	CCAGTCGACCGTGCCGCCTGTGAGGTGCACCTTG	
		pYZ034V	P-YZ042	CGGCCGGGCGCGGCCG	pCS005[1]
			P-LB236	GGCACGGTCGACTGGAGC	
pYZ035	In-Fusion	pYZ035I	P-YZ021	GCCCGGCCGCCGGTGTTGCTGCACGCCAGTGGAC	pAR127[6]
			P-YZ031	GTCCACGTGGACCTGGCCAAGGTTGGTGAGAAAGAACTC	
		pYZ035V	P-YZ043	CACCGGCGGCCGGGCGC	pCS005[1]
			P-YZ032	CAGGTCCACGTGGACGGCG	
pYZ036	In-Fusion	pYZ036I	P-YZ021	GCCCGGCCGCCGGTGTTGCTGCACGCCAGTGGAC	pAR127[6]
			P-YZ029	GACCGTGCCGCCGTCGAGGTGCACCTTGCCAAGGTTG	
		pYZ036V	P-YZ043	CACCGGCGGCCGGGCGC	pCS005[1]
			P-YZ030	GACGGCGGCACGGTCG	
pYZ038	In-Fusion	pYZ038I	P-LB241	GACGAGGTGCCCGCCAACAAGCGCCCACTCTGGTTC	pAR127[6]
			P-LB242	CCAGTCGACGGGCACGCCTGTGAGGTGCACCTTG	
		pYZ038V	P-LB244	GGCGGGCACCTCGTCC	pCS004.1[1]
			P-LB243	GTGCCCGTCGACTGGCG	
pYZ040	In-Fusion	pYZ040I	P-YZ052	CCCGACGCGCCGCCCACTGCACACGCTGCCCTTC	pAR127[6]
			P-YZ054	GGGGTAGACGGGCAGGATGAGAGGAGTCCCTCGG	
		pYZ040V	P-YZ053	GGGCGGCGCGTCGGGC	pCS004.1[1]
			P-YZ055	CTGCCCGTCTACCCCTTCG	
pYZ041	In-Fusion	pYZ041I	P-YZ058	GACGGGCAGGTCGACAGGAGTCCCTCGGGGAGC	pAR127[6]
			P-YZ056	GTCGCCGTCCATCTGATCCTCCAGCCCAACACACG	
		pYZ041V	P-YZ059	GTCGACCTGCCCGTCTACC	pCS004.1[1]
			P-YZ057	CAGATGGACGGCGACGAGG	
pYZ042	In-Fusion	pYZ042I	P-YZ062	GGGGTAGACGGGCAGGTCGAGAGGAGTCCCTCGG	pAR127[6]
			P-YZ060	CACCTCGTCCTGGAACCCAACACACGGCAGGCC	

Plasmid	Cloning Method	Fragme nts	Primer Name	Primer Sequence 5'-3'	Template
		pYZ042V	P-YZ063	GACCTGCCCGTCTACCCC	pCS004.1[1]
			P-YZ061	TTCCAGGACGAGGTGGACGTTG	
pYZ043	In-Fusion	pYZ043I	P-YZ064	CCGCCCGCCCTGCCGACTGCACACGCTGCC	pAR127[6]
			P-YZ066	GACGGGCACTCCGCGGAGGTGCACCTTGCCAAGG	
		pYZ043V	P-YZ065	CGGCAGGGCGGGCGGC	pCS004.1[1]
			P-YZ067	CGCGGAGTGCCCGTCGAC	
pYZ044	In-Fusion	pYZ044I	P-YZ068	GACGAGGTGCCCGCCAAGCGCCCACTCTGGTTCATC	pAR127[6]
			P-LB242	CCAGTCGACGGGCACGCCTGTGAGGTGCACCTTG	
		pYZ044V	P-LB244	GGCGGGCACCTCGTCC	pCS004.1[1]
			P-LB243	GTGCCCGTCGACTGGCG	
pYZ045	In-Fusion	pYZ045I	P-YZ070	CCGGACGAGGTGCCCAACAAGCGCCCACTCTGGTTC	pAR127[6]
			P-LB242	CCAGTCGACGGGCACGCCTGTGAGGTGCACCTTG	
		pYZ045V	P-YZ069	GTTGGGCACCTCGTCCGG	pCS004.1[1]
			P-LB243	GTGCCCGTCGACTGGCG	
pYZ046	In-Fusion	pYZ046I	P-LB242	CCAGTCGACGGGCACGCCTGTGAGGTGCACCTTG	pYZ038
			P-LB241	GACGAGGTGCCCGCCAACAAGCGCCCACTCTGGTTC	
		pYZ046V	P-LB243	GTGCCCGTCGACTGGCG	pCS008[1]
			P-LB244	GGCGGGCACCTCGTCC	
pSR008 (PIKS M5-TE)	In-Fusion	pSR008I	AR26_for	GAGGACCGAAGGAGCTAACC	pAR328
			PRSR016_rev	GTACGCCTCGTGGAGGTG	
		pSR008V	PRSR015_fwd	CTCCACGAGGCGTACTCCGGGGCCGACACC	pSR006
			AR27_rev	GGTTAGCTCCTTCGGTCCTC	
pSR015 (PIKS	In-Fusion	pSR015I	PRSR027_fwd	AACAAGCGCCCACTCTG	pAR264[4]

Plasmid	Cloning Method	Fragments	Primer Name	Primer Sequence 5'-3'	Template
M5*-TE)			PRSR028_rev	GCCTGTGAGGTGCACC	
		pSR015V	PRSR017_fwd	GTGCACCTCACAGGCCTCACCATCGACTGGGC	pSR008
			PRSR018_rev	GAGTGGGCGCTTGTTCGAGGACGTGCCGCGTAC	
pAR268 (M5-M6-TE)	In-Fusion	pAR268I	AR474_for	GGAGATATACATATGGGATCCAGCGGTGACAACGGCATGAC	genomic DNA ATCC 11635
			AR473_rev	GGTGATGATGCTCGAGCGAATTCCCTCCGCCCAG	
		pAR268V	AR202_for	CTCGAGCATCATCACCACCAC	pET22b(+) - Novagen
			AR72_rev	CATATGTATATCTCCTTCTTAAAGTTAAAC	
pSR022 (M5*-M6-TE)	In-Fusion	pSR022I	PRSR040_fwd	AGCGGTGACAACGGCAT	pMJD142
			AR27_rev	GGTTAGCTCCTTCGGTCCTC	
		pSR022V	AR26_for	GAGGACCGAAGGAGCTAACC	pAR18[4]
			PRSR041_rev	GCCGTTGTCACCGCTGGATCCGGCGCCTTTTTCG	
pMJD105 (M5-M6*-TE)	In-Fusion	pMJD105I	PRMJD160_fwd	CCGAAGGATGCCGACGACCCGATCGCGATC	pMJD077[3]
			AR473_rev	GGTGATGATGCTCGAGCGAATTCCCTCCGCCCAG	
		pMJD105V	AR202_for	CTCGAGCATCATCACCACCAC	pAR268
			PRMJD161_rev	GTCGGCATCCTTCGGCAC	
pSR024 (M5*-M6*-TE)	In-Fusion	pSR024I	PRSR041_fwd	AGCGGTGACAACGGCAT	PMJD147
			AR27_rev	GGTTAGCTCCTTCGGTCCTC	
		PSR024V	AR26_for	GAGGACCGAAGGAGCTAACC	PAR18[4]

Plasmid	Cloning Method	Fragments	Primer Name	Primer Sequence 5'-3'	Template
			PRSR040_rev	GCCGTTGTCACCGCTGGATCCGGCGCCTTTTTCG	

Table S2. DNA and amino acid sequences of plasmids which are not published elsewhere and were used as a template to generate the plasmids of the constructs used in this study. All plasmids were based on a pET22b vector backbone.

Construct	Sequence form start to stop codon
pAR328 (PIKS M5)	MSAWSHPQFEKGGGSGGGSGGSAWSHPQFEKGAGSANNEDKLRDYLKRVTAELQQNTRRLREIEGRTHEPVAIVGMACRLPGGVASPEDLWQLVAGDGDAISEFPQDRGWDVEGLYDPDP DASGRTYCRSGGFLHDAGEFDADFFGISPREALAMDPQQRLSLTTAWEAIESAGIDPTALKGSGLGVFVGGWHTGYTSGQTTAVQSPELEGHLVSGAALGFLSGRIAYVLGTDGPALTVDTACS SSLVALHLAVQALRKGECDMALAGGVTVMPNADLFVQFSRQRGLAADGRSKAFATSADGFGPAEGAGVLLVERLSDARRNGHRILAVVRGSAVNQDGASNGLTAPHGPSQQRVIRRALADA RLAPGDVDVVEAHGTGTRLGDPIEAQALIATYGQEKSSEQPLRLGALKSNIGHTQAAAGVAGVIKMVQAMRHGLLPKTLHVDEPSDQIDWSAGTVELLTEAVDWPEKQDGGLRRAAVSSFGI SGTNAHVVLEEAPAVEDSPAVEPPAGGGVVPWPVSAKTPAALDAQIGQLAAYADGRTDVDPAVAARALVDSRTAMEHRAVAVGDSREALRDALRMPEGLVRGTSSDVGRVAFVFPGQGTQ WAGMGAELLDSSPEFAASMAECETALSRYVDWSLEAVVRQEPGAPTLDRVDVVQPVTFAVMVSLAKVWQHHGITPQAVVGHSQGEIAAAYVAGALTLDDAARVVTLRSKSIAAHLAGKGG MISLALDEAAVLKRLSDFDGLSVAAVNGPTATVVSGDPTQIEELARTCEADGVRARIIPVDYASHSRQVEIIEKELAEVLAGLAPQAPHVPFFSTLEGTWITEPVLDGTYWYRNLRHRVGFAPA VETLAVDGFTHFIEVSAHPVLTMTLPETVTGLGTLRREQGGQERLVTSLAEAWANGLTIDWAPILPTATGHHPELPTYAFQTERFWLQSSAPTSAADDWRYRVEWKPLTASGQADLSGRWI VAVGSEPEAELLGALKAAGAEVDVLEAGADDDREALAARLTALTTGDGFTGVVSLLDDLVPQVAWVQALGDAGIKAPLWSVTQGAVSVGRLDTPADPDRAMLWGLGRVVALEHPERWAGL VDLPAQPDAAALAHLVTALSGATGEDQIAIRTTGLHARRLARAPLHGRRPTRDWQPHGTVLITGGTGALGSHAARWMAHHGAEHLLLVSRSGEQAPGATQLTAELTASGARVTIAACDVAD PHAMRTLLDAIPAETPLTAVVHTAGAPGGDPLDVTGPEDIARILGAKTSGAEVLDDLLRGTPLDAFVLYSSNAGVWGSGSQGVYAAANAHLDALAARRRARGETATSVAWGLWAGDGMGR GADDAYWQRRGIRPMSPDRALDELAKALSHDETFVAVADVDWERFAPAFTVSRPSLLLDGVPEARQALAAPVGAPAPGDAAVAPTGQSSALAAITALPEPERRPALLTLVRTHAAAVLGHSS PDRVAPGRAFTELGFDSLTAVQLRNQLSTVVGNRLPATTVFDHPTPAALAAHLHEAYLAPAEPAPTDWEGRVRRALAELPLDRLRDAGVLDTVLRLTGIEPEPGSGGSDGGAADPGAEPEAS IDDLDAEALIRMALGPRNTLEHHHHHHHH
pAR328 (PIKS M5)	ATGAGCGCTTGGAGCCATCCACAATTTGAGAAGGGTGGAGGTTCTGGCGGTGGATCGGGAGGTTCAGCGTGGAGCCACCCGCAGTTCGAAAAAGGCGCCGGATCCGCGAACAACGAAGACA AGCTCCGCGACTACCTCAAGCGCGTCACCGCCGAGCTGCAGCAGAACACCAGGCGTCTGCGCGAGATCGAGGGACGCACGCACGAGCCGGTGGCGATCGTGGGCATGGCCTGCCGCCTGCCGG GCGGTGTCGCCTCGCCCGAGGACCTGTGGCAGCTGGTGGCCGGGGACGGGGACGCGATCTCGGAGTTCCCGCAGGACCGCGGCTGGGACGTGGAGGGGCTGTACGACCCCGACCCGGACGCG TCCGGCAGGACGTACTGCCGGTCCGGCGGATTCCTGCACGACGCCGGCGAGTTCGACGCCGACTTCTTCGGGATCTCGCCGCGCGAGGCCCTCGCCATGGACCCGCAGCAGCGACTGTCCCTC ACCACCGCGTGGGAGGCGATCGAGAGCGCGGGCATCGACCCGACGGCCCTGAAGGGCAGCGGCCTCGGCGTCTTCGTCGGCGGCTGGCACACCGGCTACACCTCGGGGCAGACCACCGCCGTG CAGTCGCCCGAGCTGGAGGGCCACCTGGTCAGCGGCGCGGCGCTGGGCTTCCTGTCCGGCCGTATCGCGTACGTCCTCGGTACGGACGGACCGGCCCTGACCGTGGACACGGCCTGCTCGTCC TCGCTGGTCGCCCTGCACCTCGCCGTGCAGGCCCTCCGCAAGGGCGAGTGCGACATGGCCCTCGCCGGTGGTGTCACGGTCATGCCCAACGCGGACCTGTTCGTGCAGTTCAGCCGGCAGCGC GGGCTGGCCGCGGACGGCCGGTCGAAGGCGTTCGCCACCTCGGCGGACGGCTTCGGCCCCGCGGAGGGCGCCGGAGTCCTGCTGGTGGAGCGCCTGTCGGACGCCCGCCGCAACGGACACCGG ATCCTCGCGGTCGTCCGCGGCAGCGCGGTCAACCAGGACGGCGCCAGCAACGGCCTCACGGCTCCGCACGGGCCCTCCCAGCAGCGCGTCATCCGACGGGCCCTGGCGGACGCCCGGCTCGCG CCGGGTGACGTGGACGTCGTCGAGGCGCACGGCACGGGCACGCGGCTCGGCGACCCGATCGAGGCGCAGGCCCTCATCGCCACCTACGGCCAGGAGAAGAGCAGCGAACAGCCGCTGAGGCT GGGCGCGTTGAAGTCGAACATCGGGCACACGCAGGCCGCGGCCGGTGTCGCAGGTGTCATCAAGATGGTCCAGGCGATGCGCCACGGACTGCTGCCGAAGACGCTGCACGTCGACGAGCCCT CGGACCAGATCGACTGGTCGGCGGGCACGGTGGAACTCCTCACCGAGGCCGTCGACTGGCCGGAGAAGCAGGACGGCGGGCTGCGCCGCGCGGCTGTCTCCTCCTTCGGCATCAGCGGGACG AACGCGCACGTCGTCCTGGAGGAGGCCCCGGCGGTCGAGGACTCCCCGGCCGTCGAGCCGCCGGCCGGTGGCGGTGTGGTGCCGTGGCCGGTGTCCGCGAAGACTCCGGCCGCGCTGGACGCC CAGATCGGGCAGCTCGCCGCGTACGCGGACGGTCGTACGGACGTGGATCCGGCGGTGGCCGCCCGCGCCCTGGTCGACAGCCGTACGGCGATGGAGCACCGCGCGGTCGCGGTCGGCGACAGC CGGGAGGCACTGCGGGACGCCCTGCGGATGCCGGAAGGACTGGTACGCGGCACGTCCTCGGACGTGGGCCGGGTGGCGTTCGTCTTCCCCGGCCAGGGCACGCAGTGGGCCGGCATGGGCGC CGAACTCCTTGACAGCTCACCGGAGTTCGCTGCCTCGATGGCCGAATGCGAGACCGCGCTCTCCCGCTACGTCGACTGGTCTCTTGAAGCCGTCGTCCGACAGGAACCCGGCGCACCCACGCT CGACCGCGTCGACGTCGTCCAGCCCGTGACCTTCGCTGTCATGGTCTCGCTGGCGAAGGTCTGGCAGCACCACGGCATCACCCCCCAGGCCGTCGTCGGCCACTCGCAGGGCGAGATCGCCGC CGCGTACGTCGCCGGTGCACTCACCCTCGACGACGCCGCCCGCGTCGTCACCCTGCGCAGCAAGTCCATCGCCGCCCACCTCGCCGGCAAGGGCGGCATGATCTCCCTCGCCCTCGACGAGGCG GCCGTCCTGAAGCGACTGAGCGACTTCGACGGACTCTCCGTCGCCGCCGTCAACGGCCCCACCGCCACCGTCGTCTCCGGCGACCCGACCCAGATCGAGGAACTCGCCCGCACCTGCGAGGCC GACGGCGTCCGTGCGCGGATCATCCCGGTCGACTACGCCTCCCACAGCCGGCAGGTCGAGATCATCGAGAAGGAGCTGGCCGAGGTCCTCGCCGGACTCGCCCCGCAGGCTCCGCACGTGCCG TTCTTCTCCACCCTCGAAGGCACCTGGATCACCGAGCCGGTGCTCGACGGCACCTACTGGTACCGCAACCTGCGCCATCGCGTGGGCTTCGCCCCCGCCGTGGAGACCTTGGCGGTTGACGGC TTCACCCACTTCATCGAGGTCAGCGCCCACCCCGTCCTCACCATGACCCTCCCCGAGACCGTCACCGGCCTCGGCACCCTCCGCCGCGAACAGGGAGGCCAGGAGCGTCTGGTCACCTCACTCG CCGAAGCCTGGGCCAACGGCCTCACCATCGACTGGGCGCCCATCCTCCCCACCGCAACCGGCCACCACCCCGAGCTCCCCACCTACGCCTTCCAGACCGAGCGCTTCTGGCTGCAGAGCTCCGC

Construct	Sequence form start to stop codon
	GCCCACCAGCGCCGCCGACGACTGGCGTTACCGCGTCGAGTGGAAGCCGCTGACGGCCTCCGGCCAGGCGGACCTGTCCGGGCGGTGGATCGTCGCCGTCGGGAGCGAGCCAGAAGCCGAGCT<br>GCTGGGCGCGCTGAAGGCCGCGGGAGCGGAGGTCGACGTACTGGAAGCCGGGGCGGACGACGACCGTGAGGCCCTCGCCGCCCGGCTCACCGCACTGACGACCGGCGACGGCTTCACCGGCGT<br>GGTCTCGCTCCTCGACGACCTCGTGCCACAGGTCGCCTGGGTGCAGGCACTCGGCGACGCCGGAATCAAGGCGCCCCTGTGGTCCGTCACCCAGGGCGCGGTCTCCGTCGGACGTCTCGACAC<br>CCCCGCCGACCCCGACCGGCCATGCTCTGGGGCCTCGGCCGCGTCGTCGCCCTTGAGCACCCCGAACGCTGGGCCGGCCTCGTCGACCTCCCCGCCCAGCCCGATGCCGCCGCCCTCGCCCAC<br>CTCGTCACCGCACTCTCCGGCGCCACCGGCGAGGACCAGATCGCCATCCGCACCACCGGACTCCACGCCCGCCGCCTCGCCCGCGCACCCCTCCACGGACGTCGGCCCACCCGCGACTGGCAGC<br>CCCACGGCACCGTCCTCATCACCGGCGGCACCGGAGCCCTCGGCAGCCACGCCGCACGCTGGATGGCCCACCACGGAGCCGAACACCTCCTCCTCGTCAGCCGCAGCGGCGAACAAGCCCCCGG<br>AGCCACCCAACTCACCGCCGAACTCACCGCATCGGGCGCCCGCGTCACCATCGCCGCCTGCGACGTCGCCGACCCCCACGCCATGCGCACCCTCCTCGACGCCATCCCCGCCGAGACGCCCCTC<br>ACCGCCGTCGTCCACACCGCCGGCGCACCGGGCGGCGATCCGCTGGACGTCACCGGCCCGGAGGACATCGCCCGCATCCTGGGCGCGAAGACGAGCGGCGCCGAGGTCCTCGACGACCTGCTC<br>CGCGGCACTCCGCTGGACGCCTTCGTCCTCTACTCCTCGAACGCCGGGGTCTGGGGCAGCGGCAGCCAGGGCGTCTACGCGGCGGCCAACGCCCACCTCGACGCGCTCGCCGCCCGGCGCCGCG<br>CCCGGGGCGAGACGGCGACCTCGGTCGCCTGGGGCCTCTGGGCCGGCGACGGCATGGGCCGGGGCGCCGACGACGCGTACTGGCAGCGTCGCGGCATCCGTCCGATGAGCCCCGACCGCGCCC<br>TGGACGAACTGGCCAAGGCCCTGAGCCACGACGAGACCTTCGTCGCCGTGGCCGATGTCGACTGGGAGCGGTTCGCGCCCGCGTTCACGGTGTCCCGTCCCAGCCTTCTGCTCGACGGCGTCC<br>CGGAGGCCCGGCAGGCGCTCGCCGCACCCGTCGGTGCCCCGGCTCCCGGCGACGCCGCCGTGGCGCCGACCGGGCAGTCGTCGGCGCTGGCCGCGATCACCGCGCTCCCCGAGCCCGAGCGCCG<br>GCCGGCGCTCCTCACCCTCGTCCGTACCCACGCGGCGGCCGTACTCGGCCATTCCTCCCCCGACCGGGTGGCCCCCGGCCGTGCCTTCACCGAGCTCGGCTTCGACTCGCTGACGGCCGTGCAG<br>CTCCGCAACCAGCTCTCCACGGTGGTCGGCAACAGGCTCCCCGCCACCACGGTCTTCGACCACCCGACGCCCGCCGCACTCGCCGCGCACCTCCACGAGGCGTACCTCGCACCGGCCGAGCCGG<br>CCCCGACGGACTGGGAGGGGCGGGTGCGCCGGGCCCTGGCCGAACTGCCCCTCGACCGGCTGCGGGACGCGGGGGTCCTCGACACCGTCCTGCGCCTCACCGGCATCGAGCCCGAGCCGGGTT<br>CCGGCGGTTCGGACGGCGGCGCCGCCGACCCTGGTGCGGAGCCGGAGGCGTCGATCGACGACCTGGACGCCGAGGCCCTGATCCGGATGGCTCTCGGCCCCCGTAACACCCTCGAGCATCATC<br>ACCACCACCACCACCACTGA
pSR006 (PIKS M6-TE)	MTSSNEQLVDALRASLKENEELRKESRRRADRRQEPMAIVGMSCRFAGGIRSPEDLWDAVAAGKDLVSEVPEERGWDIDSLYDPVPGRKGTTYVRNAAFLDDAAGFDAAFFGISPREALAM<br>DPQQRQLLEASWEVFERAGIDPASVRGTDVGVYVGCGYQDYAPDIRVAPEGTGGYVVTGNSSAVASGRIAYSLGLEGPAVTVDTACSSSLVALHLALKGLRNGDCSTALVGGVAVLATPGAFIE<br>FSSQQAMAADGRTKGFASAADGLAWGEGVAVLLLERLSDARRKGHRVLAVVRGSAINQDGASNGLTAPHGPSQQRLIRQALADARLTSSDVDVVEGHGTGTRLGDPIEAQALLATYGQGRAP<br>GQPLRLGTLKSNIGHTQAASGVAGVIKMVQALRHGVLPKTLHVDEPTDQVDWSAGSVELLTEAVDWPERPGRLRRAGVSAFGVGGTNAHVVLEEAPAVEESPAVEPPAGGVVPWPVSAKT<br>SAALDAQIGQLAAYAEDRTDVDPAVAARALVDSRTAMEHRAVAVGDSREALRDALRMPEGLVRGTVTDPGRVAFVFPGQGTQWAGMGAELLDSSPEFAAAMAECETALSPYVDWSLEAVV<br>RQAPSAPTLDRVDVVQPVTFAVMVSLAKVWQHHGITPEAVIGHSQGEIAAAYVAGALTLDDAARVVTLRSKSIAAHLAGKGGMISLALSEEATRQRIENLHGLSIAAVNGPTATVVSGDPTQIQ<br>ELAQACEADGIRARIIPVDYASHSAHVETIENELADVLAGLSPQTPQVPFFSTLEGTWITEPALDGGYWYRNLRHRVGFAPAVETLATDEGFTHFIEVSAHPVLTMTLPDKVTGLATLRREDG<br>GQHRLTTSLAEAWANGLALDWASLLPATGALSPAVPDLPTYAFQHRSYWISPAGPGEAPAHTASGREAVAETGLAWGPGAEDLDEEGRRSAVLAMVMRQAASVLRCDSPEEVPVDRPLREI<br>GFDSLTAVDFRNRVNRLTGLQLPPTVVFQHPTPVALAERISDELAERNWAVAEPSDHEQAEEEKAAAPAGARSGADTGAGAGMFRALFRQAVEDDRYGEFLDVLAEASAFRPQFASPEACSE<br>RLDPVLLAGGPTDRAEGRAVLVGCTGTAANGGPHEFLRLSTSFQEERDFLAVPLPGYGTGTGTGTALLPADLDTALDAQARAILRAAGDAPVVLLGHSGGALLAHELAFRLERAHGAPPAGIV<br>LVDPYPPGHQEPIEVWSRQLGEGLFAGELEPMSDARLLAMGRYARFLAGPRPGRSSAPVLLVRASEPLGDWQEERGDWRAHWDLPHTVADVPGDHFTMMRDHAPAVAEAVLSWLDAIEG<br>IEGAGKLEHHHHHHHH
pSR006 (PIKS M6-TE)	ATGACGAGTTCCAACGAAcagttgGTGGACGCTCTGCGCGCCTCTCTCAAGGAGAACGAAGAACTCCGGAAAGAGAGCCGTCGCCGGGCCGACCGTCGGCAGGAGCCCATGGCGATCGTCGGCA<br>TGAGCTGCCGGTTCGCGGGCGGAATCCGGTCCCCCGAGGACCTCTGGGACGCCGTCGCCGCGGGCAAGGACCTGGTCTCCGAGGTACCGGAGGAGCGCGGCTGGGACATCGACTCCCTCTACG<br>ACCCGGTGCCCGGGCGCAAGGGCACGACGTACGTCCGCAACGCCGCGTTCCTCGACGACGCCGCCGGATTCGACGCGGCCTTCTTCGGGATCTCGCCGCGCGAGGCCCTCGCCATGGACCCGC<br>AGCAGCGGCAGCTCCTCGAAGCCTCCTGGGAGGTCTTCGAGCGGGCCGGCATCGACCCCGCGTCGGTCCGCGGCACCGACGTCGGCGTGTACGTGGGCTGTGGCTACCAGGACTACGCGCCGG<br>ACATCCGGGTCGCCCCCGAAGGCACCGGCGGTTACGTCGTCACCGGCAACTCCTCCGCCGTGGCCTCCGGGCGCATCGCGTACTCCCTCGGCCTGGAGGGACCCGCCGTGACCGTGGACACGG<br>CGTGCTCCTCTTCGCTCGTCGCCCTGCACCTCGCCCTGAAGGGCCTGCGGAACGGCGACTGCTCGACGGCACTCGTGGGCGGCGTGGCCGTCCTCGCGACGCCGGGCGCGTTCATCGAGTTCA<br>GCAGCCAGCAGGCCATGGCCGCCGACGGCCGGACCAAGGGCTTCGCCTCGGCGGCGGACGGCCTCGCCTGGGGCGAGGGCGTCGCCGTACTCCTCCTCGAACGGCTCTCCGACGCGCGGCGCA<br>AGGGCCACCGGGTCCTGGCCGTCGTGCGCGGCAGCGCCATCAACCAGGACGGCGCGAGCAACGGCCTCACGGCTCCGCACGGGCCCTCCCAGCAGCGCCTGATCCGCCAGGCCCTGGCCGACG<br>CGCGGCTCACGTCGAGCGACGTGGACGTCGTGGAGGGCCACGGCACGGGGACCCGTCTCGGCGACCCGATCGAGGCGCAGGCGCTGCTCGCCACGTACGGGCAGGGGCGCGCCCCGGGGCAGC<br>CGCTGCGGCTGGGGACGCTGAAGTCGAACATCGGGCACACGCAGGCCGCTTCGGGTGTCGCCGGTGTCATCAAGATGGTGCAGGCGCTGCGCCACGGGGTGCTGCCGAAGACCCTGCACGTG<br>GACGAGCCGACGGACCAGGTCGACTGGTCGGCCGGTTCGGTCGAGCTGCTCACCGAGGCCGTGGACTGGCCGGAGCGGCCGGGCCGGCTCCGCCGGGCGGGCGTCTCCGCGTTCGGCGTGGGC<br>GGGACGAACGCGCACGTCGTCCTGGAGGAGGCCCCGGCGGTCGAGGAGTCCCCTGCCGTCGAGCCGCCGGCCGGTGGCGGCGTGGTGCCGTGGCCGGTGTCCGCGAAGACCTCGGCCGCACTG<br>GACGCCCAGATCGGGCAGCTCGCCGCATACGCGGAAGACCGCACGGACGTGGATCCGGCGGTGGCCGCCCGCGCCCTGGTCGACAGCCGTACGGCGATGGAGCACCGCGCGGTCGCGGTCGGC<br>GACAGCCGGGAGGCACTGCGGGACGCCCTGCGGATGCCGGAAGGACTGGTACGGGGCACGGTCACCGATCCGGGCCGGGTGGCGTTCGTCTTCCCCGGCCAGGGCACGCAGTGGGCCGGCAT<br>GGGCGCCGAACTCCTCGACAGCTCACCCGAATTCGCCGCCGCCATGGCCGAATGCGAGACCGCACTCTCCCCGTACGTCGACTGGTCTCTCGAAGCCGTCGTCCGACAGGCTCCCAGCGCACC<br>GACACTCGACCGCGTCGACGTCGTCCAGCCCGTCACCTTCGCCGTCATGGTCTCCCTCGCCAAGGTCTGGCAGCACCACGGCATCACCCCCGAGGCCGTCATCGGCCACTCCCAGGGCGAGAT<br>CGCCGCCGCGTACGTCGCCGGTGCCCTCACCCTCGACGACGCCGCTCGTGTCGTGACCCTCCGCAGCAAGTCCATCGCCGCCCACCTCGCCGGCAAGGGCGGCATGATCTCCCTCGCCCTCAGC<br>GAGGAAGCCACCCGGCAGCGCATCGAGAACCTCCACGGACTGTCGATCGCCGCCGTCAACGGGCCTACCGCCACCGTGGTTTCGGGCGACCCCACCCAGATCCAAGAACTTGCTCAGGCGTGT

Construct	Sequence form start to stop codon
	GAGGCCGACGGCATCCGCGCACGGATCATCCCCGTCGACTACGCCTCCCACAGCGCCCACGTCGAGACCATCGAGAACGAACTCGCCGACGTCCTGGCGGGGTTGTCCCCCCAGACACCCCAG<br>GTCCCCTTCTTCTCCACCCTCGAAGGCACCTGGATCACCGAACCCGCCCTCGACGGCGGCTACTGGTACCGCAACCTCCGCCATCGTGTGGGCTTCGCCCCGGCCGTCGAGACCCTCGCCACCG<br>ACGAAGGCTTCACCCACTTCATCGAGGTCAGCGCCCACCCCGTCCTCACCATGACCCTCCCCGACAAGGTCACCGGCCTGGCCACCCTCCGACGCGAGGACGGCGGACAGCACCGCCTCACCAC<br>CTCCCTTGCCGAGGCCTGGGCCAACGGCCTCGCCCTCGACTGGGCCTCCCTCCTGCCCGCCACGGGCGCCCTCAGCCCCGCCGTCCCCGACCTCCCGACGTACGCCTTCCAGCACCGCTCGTAC<br>TGGATCAGCCCCGCGGGTCCCGGCGAGGCGCCCGCGCACACCGCTTCCGGGCGCGAGGCCGTCGCCGAGACGGGGCTCGCGTGGGGCCCGGGTGCCGAGGACCTCGACGAGGAGGGCCGGCGC<br>AGCGCCGTACTCGCGATGGTGATGCGGCAGGCGGCCTCCGTGCTCCGGTGCGACTCGCCCGAAGAGGTCCCCGTCGACCGCCCGCTGCGGGAGATCGGCTTCGACTCGCTGACCGCCGTCGAC<br>TTCCGCAACCGCGTCAACCGGCTGACCGGTCTCCAGCTGCCGCCCACCGTCGTGTTCCAGCACCCGACGCCCGTCGCGCTCGCCGAGCGCATCAGCGACGAGCTGGCCGAGCGGAACTGGGCC<br>GTCGCCGAGCCGTCGGATCACGAGCAGGCGGAGGAGGAGAAGGCCGCCGCTCCGGCGGGGGCCCGCTCCGGGGCCGACACCGGCGCCGGCGCCGGGATGTTCCGCGCCCTGTTCCGGCAGGCC<br>GTGGAGGACGACCGGTACGGCGAGTTCCTCGACGTCCTCGCCGAAGCCTCCGCGTTCCGCCCGCAGTTCGCCTCGCCCGAGGCCTGCTCGGAGCGGCTCGACCCGGTGCTGCTCGCCGGCGGT<br>CCGACGGACCGGGCGGAAGGCCGTGCCGTTCTCGTCGGCTGCACCGGCACCGCGGCGAACGGCGGCCCGCACGAGTTCCTGCGGCTCAGCACCTCCTTCCAGGAGGAGCGGGACTTCCTCGCC<br>GTACCTCTCCCCGGCTACGGCACGGGTACGGGCACCGGCACGGCCCTCCTCCCGGCCGATCTCGACACCGCGCTCGACGCCCAGGCCCGGGCGATCCTCCGGGCCGCCGGGGACGCCCCGGTCG<br>TCCTGCTCGGGCACTCCGGCGGCGCCCTGCTCGCGCACGAGCTGGCCTTCCGCCTGGAGCGGGCGCACGGCGCGCCGCCGGCCGGGATCGTCCTGGTCGACCCCTATCCGCCGGGCCATCAGGA<br>GCCCATCGAGGTGTGGAGCAGGCAGCTGGGCGAGGGCCTGTTCGCGGGCGAGCTGGAGCCGATGTCCGATGCGCGGCTGCTGGCCATGGGCCGGTACGCGCGGTTCCTCGCCGGCCCGCGGCC<br>GGGCCGCAGCAGCGCGCCCGTGCTTCTGGTCCGTGCCTCCGAACCGCTGGGCGACTGGCAGGAGGAGCGGGGCGACTGGCGTGCCCACTGGGACCTTCCGCACACCGTCGCGGACGTGCCGGG<br>CGACCACTTCACGATGATGCGGGACCACGCGCCGGCCGTCGCCGAGGCCGTCCTCTCCTGGCTCGACGCCATCGAGGGCATCGAGGGGGCGGGCAAGCTCGAGCATCATCACCACCACCACCA<br>CCACTGA
pMJD142 (M5*-M6-TE)	MGSSGDNGMTEEKLRRYLKRTVTELDSVTARLREVEHRAGEPIAIVGMACRFPGDVDSPESFWEFVSGGGDAIAEAPADRGWEPDPDARLGGMLAAAGDFDAGFFGISPREALAMDPQQRI<br>MLEISWEALERAGHDPVSLRGSATGVFTGVGTVDYGPRPDEAPDEVLGYVGTGTASSVASGRVAYCLGLEGPAMTVDTACSSGLTALHLAMESLRRDECGLALAGGVTVMSSPGAFTEFRSQ<br>GGLAADGRCKPFSKAADGFGLAEGAGVLVLQRLSAARREGRPVLAVLRGSAVNQDGASNGLTAPSGPAQQRVIRRALENAGVRAGDVDYVEAHGTGTRLGDPIEVHALLSTYGAERDPDDPL<br>WIGSVKSNIGHTQAAAGVAGVMKAVLALRHGEMPRTLHFDEPSPQIEWDLGAVSVVSQARSWPAGERPRRAGVSSFGISGTNAHVIVEEAPEADEPEPAPDSGPVPLVLSGRDEQAMRAQAG<br>RLADHLAREPRNSLRDTGFTLATRRSAWEHRAVVVGDRDDALAGLRAVADGRIADRTATGQARTNKRPLWFICSGMGTQWRGMGLSLMRLDSFRESILRSDEAVKPLGVKVSDLLLSTDER<br>TFDDIVHAFVSLTAIQIALIDLLTSVGLKPDGIIGHSLGEVACGYADGCLSQREAVLAAYWRGQCIKDAHLPPGSMAAVGLSWEECKQRCPAGVVPACHNSEDTVTISGPQAAVNEFVEQLKQE<br>GVFAKEVRTGGLAFHSYFMEGIAPTLLQALKKVIREPRPRSARWLSTSIPEAQWQSSLARTSSAEYNVNNLVSPVLFQEALWHIPEHAVVLEIAPHALLQAVLKRGVKSSCTIIPLMKRDHKDN<br>LEFFLTNLGKVHLTGVEVDWSPAFADARPVELPVYPFQRQRYWLPIPTGGRARDEDDDWRYQVVWREAEWESASLAGRVLLVTGPGVPSELSDAIRSGLEQSGATVLTCDVESRSTIGTALE<br>AADTDALSTVVSLLSRDGEAVDPSLDALALVQALGAAGVEAPLWVLTRNAVQVADGELVDPAQAMVGGLGRVVGIEQPGRWGGLVDLVDADAASIRSLAAVLADPRGEEQVAIRADGIKVA<br>RLVPAPARAARTRWSPRGTVLVTGGTGGIGAHVARWLARSGAEHLVLLGRRGADAPGASELREELTALGTGVTIAACDVADRARLEAVLAAERAEGRTVSAVMHAAGVSTSTPLDDLTEAE<br>FTEIADVKVRGTVNLDELCPDLDAFVLFSSNAGVWGSPGLASYAAANAFLDGFARRRRSEGAPVTSIAWGLWAGQNMAGDEGGEYLRSQGLRAMDPDRAVEELHITLDHGQTSVSVVDMD<br>RRRFVELFTAARHRPLFDEIAGARAEARQSEEGPALAQRLAALSTAERREHLAHLIRAEVAAVLGHGDDAAIDRDRAFRDLGFDSMTAVDLRNRLAAVTGVREAATVVFDHPTITRLADHYL<br>ERLVGAAEAEQAPALVREVPKDADDPIAIVGMACRFPGGVHNPGELWEFIVGGGDAVTEMPTDRGWDLDALFDPDPQRHGTSYSRHGAFLDGAADFDAAFFGISPREALAMDPQQRQVLET<br>TWELFENAGIDPHSLRGSDTGVFLGAAYQGYGQDAVVPEDSEGYLLTGNSSAVVSGRVAYVLGLEGPAVTVDTACSSSLVALHSACGSLRDGDCGLAVAGGVSVMAGPEVFTEFSRQGGLAVD<br>GRCKAFSAEADGFGFAEGVAVVLLQRLSDARRAGRQVLGVVAGSAINQDGASNGLAAPSGVAQQRVIRKAWARAGITGADVAVVEAHGTGTRLGDPVEASALLATYGKSRGSSGPVLLGSVKS<br>NIGHAQAAAGVAGVIKVVLGLNRGLVPPMLCRGERSPLIEWSSGGVELAEAVSPWPPAADGVRRAGVSAFGVSGTNAHVIIAEPPEPEPLPEPGPVGVLAAANSVPVLLSARTETALAAQARLL<br>ESAVDDSVPLTALASALATGRAHLPRRAALLAGDHEQLRGQLRAVAEGVAAPGATTGTASAGGVVFVFPGQGAQWEGMARGLLSVPVFAESIAECDAVLSEVAGFSASEVLEQRPDAPSLERV<br>DVVQPVLFSVMVSLARLWGACGVSPSAVIGHSQGEIAAAVVAGVLSLEDGVRVVALRAKALRALAGKGGMVSLAAPGERARALIAPWEDRISVAAVNSPSSVVVSGDPEALAELVARCEDEGV<br>RAKTLPVDYASHSRHVEEIRETILADLDGISARRAAIPLYSTLHGERRDGADMGPRYWYDNLRSQVRFDEAVSAAVADGHATFVEMSPHPVLTAAVQEIAADAVAIGSLHRDTAEEHLIAELA<br>RAHVHGVAVDWRNVFPAAPPVALPNYPFEPQRYWLAPEVSDQLADSRYRVDWRPLATTPVDLEGGFLVHGSAPESLTSAVEKAGGRVVPVASADREALAAALREVPGEVAGVLSVHTGAA<br>THLALHQSLGEAGVRAPLWLVTSRAVALGESEPVDPEQAMVWGLGRVMGLETPERWGGLVDLPAEPAPGDGEAFVACLGADGHEDQVAIRDHARYGRRLVRAPLGTRESSWEPAGTALVT<br>GGTGALGGHVARHLARCGVEDLVLVSRRGVDAPGAAELEAELVALGAKTTITACDVADREQLSKLLEELRGQGRPVRTVVHTAGVPESRPLHEIGELESVCAAKVTGARLLDELCPDAETFVL<br>FSSGAGVWGSANLGAYSAANAYLDALAHRRRAEGRAATSVAWGAWAGEGMATGDLEGLTRRGLRPMAPERAIRALHQALDNGDTCVSIADVDWERFAVGFTAARPRPLLDELVTPAVGA<br>VPAVQAAPAREMTSQELLEFTHSHVAAILGHSSPDAVGQDQPFTELGFDSLTAVGLRNQLQQATGLALPATLVFEHPTVRRLADHIGQQLDSGTPAREASSALRDGYRQAGVSGRVRSYLDLL<br>AGLSDFREHFDGSDGFSLDLVDMADGPGEVTVICCAGTAAISGPHEFTRLAGALRGIAPVRAVPQPGYEEGEPLPSSMAAVAAVQADAVIRTQGDKPFVVAGHSAGALMAYALATELLDRGHP<br>PRGVVLIDVYPPGHQDAMNAWLEELTATLFDRETVRMDDTRLTALGAYDRLTGQWRPRETGLPTLLVSAGEPMGPWPDDSWKPTWPFEHDTVAVPGDHFTMVQEHADAIARHIDAWLG<br>GGNSLEHHHHHHHH
pMJD142 (M5*-M6-TE)	ATGGGATCCAGCGGTGACAACGGCATGACCGAGGAAAAGCTCCGGCGCTACCTCAAGCGCACCGTCACCGAGCTCGACTCGGTGACCGCGCGCCTGCGTGAAGTCGAGCACCGGGCCGGTGA<br>GCCGATCGCGATCGTCGGCATGGCGTGCCGGTTCCCCGGCGACGTGGACTCGCCGGAGTCGTTCTGGGAGTTCGTGTCCGGCGGCGGGGACGCCATCGCGGAGGCCCCCGCCGACCGCGGCTG<br>GGAGCCGGACCCCGACGCGCGGCTGGGCGGGATGCTCGCGGCCGCGGGCGACTTCGACGCGGGCTTCTTCGGGATCTCGCCGCGCGAGGCGCTGGCGATGGACCCGCAGCAGCGGATCATGCT<br>GGAGATCTCGTGGGAGGCGCTGGAGCGCGCCGGCCACGATCCGGTGTCCCTGCGCGGCAGCGCGACCGGGGTGTTCACCGGTGTCGGCACCGTGGACTACGGCCCGCGACCCGACGAGGCCCC

**Construct**	**Sequence form start to stop codon**
	GGACGAGGTCCTGGGCTACGTCGGCACCGGCACCGCCTCCAGCGTCGCCTCCGGCCGGGTCGCCTACTGCCTGGGCCTGGAAGGCCCGGCGATGACCGTCGACACCGCCTGTTCCTCCGGGCT<br>CACCGCCCTGCACCTGGCGATGGAGTCGCTGCGCCGGGACGAGTGCGGCCTGGCGCTGGCCGGCGGCGTGACGGTGATGAGCAGTCCCGGGGCGTTCACCGAGTTCCGCAGCCAGGGCGGGCT<br>CGCCGCCGACGGCCGCTGCAAGCCGTTCTCGAAGGCCGCCGACGGGTTCGGCCTGGCCGAGGGTGCCGGGGTCCTGGTGCTGCAACGGCTGTCGGCCGCGCGGCGGGAGGGCAGACCGGTGCT<br>GGCCGTGCTGCGGGGCTCGGCGGTCAACCAGGACGGCGCCAGCAACGGGCTGACCGCGCCGAGCGGACCCGCGCAGCAGCGGGTCATCCGCCGGGCGCTGGAGAACGCCGGTGTCCGGGCGG<br>GCGACGTCGACTACGTGGAGGCCCACGGCACCGGCACCAGGCTGGGCGACCCCATCGAGGTGCACGCGCTGCTCTCGACCTACGGCGCGGAACGCGACCCGGACGATCCACTGTGGATCGGTT<br>CGGTCAAGTCCAACATCGGCCACACCCAGGCCGCCGCCGGCGTCGCCGGGGTGATGAAGGCGGTGCTGGCGCTGCGGCACGGCGAGATGCCGCGCACGCTGCACTTCGACGAGCCCTCGCCGC<br>AGATCGAGTGGGACCTGGGCGCGGTGTCGGTGGTGTCGCAGGCGCGGTCGTGGCCCGCCGGCGAGAGGCCCCGCAGGGCGGGCGTCTCCTCGTTCGGCATCAGCGGCACCAACGCGCACGTC<br>ATCGTCGAAGAGGCGCCCGAGGCCGACGAGCCCGAGCCGGCACCCGACTCGGGTCCGGTCCCGCTGGTGTTGTCCGGCCGCGACGAGCAGGCGATGCGGGCGCAGGCGGGACGGCTGGCAGA<br>CCACCTCGCCCGCGAGCCGCGGAACTCGTTGCGCGACACCGGTTTCACGCTGGCCACCCGCCGCAGCGCGTGGGAGCACCGCGCGGTGGTGGTCGGCGACCGCGACGACGCCCTCGCCGGGCT<br>GCGCGCGGTGGCCGACGGCCGCATCGCCGACCGGACGGCCACCGGGCAGGCCCGAACTAACAAGCGCCCACTCTGGTTCATCTGCTCAGGGATGGGCACGCAGTGGCGCGGGATGGGGCTGA<br>GCCTCATGCGCCTGGACAGCTTCCGTGAGTCTATCCTGCGCTCCGATGAGGCTGTGAAGCCGTTGGGAGTGAAAGTGTCAGATCTGCTGTTGAGCACAGATGAGCGCACCTTTGATGACATC<br>GTGCATGCCTTTGTGAGCCTCACTGCCATCCAGATTGCCCTCATCGACCTACTGACTTCTGTGGGACTGAAACCTGACGGCATCATTGGGCACTCCTTGGGAGAGGTTGCCTGTGGCTATGC<br>AGATGGCTGTCTCTCCCAGAGAGAGGCTGTGCTTGCAGCTTACTGGCGAGGCCAGTGCATCAAAGATGCCCACCTCCCGCCTGGATCCATGGCAGCTGTTGGTTTGTCCTGGGAGGAATGTA<br>AACAGCGCTGCCCCGCTGGCGTGGTGCCTGCCTGCCACAACTCTGAGGACACCGTGACCATCTCTGGACCTCAGGCTGCAGTGAATGAATTTGTGGAGCAGCTAAAGCAAGAAGGTGTGTTT<br>GCCAAGGAGGTACGAACAGGAGGCCTGGCTTTCCACTCCTACTTCATGGAAGGAATTGCCCCCACATTGCTGCAGGCTCTCAAGAAGGTGATCCGGGAACCACGGCCGCGCTCGGCTCGATG<br>GCTCAGCACCTCTATCCCTGAGGCCCAGTGGCAGAGCAGCCTGGCCCGCACATCTTCTGCCGAGTACAATGTCAACAACCTGGTGAGCCCTGTGCTCTTCCAGGAAGCACTGTGGCACATCCC<br>TGAGCATGCCGTGGTGCTGGAGATTGCGCCCCACGCACTGTTGCAGGCTGTCCTGAAGCGAGGCGTGAAGTCCAGCTGCACCATCATTCCCTTGATGAAGAGGGATCATAAAGATAACTTGG<br>AGTTCTTTCTCACCAACCTTGGCAAGGTGCACCTCACAGGCGTGGAGGTCGACTGGAGCCCCGCCTTCGCCGACGCGCGGCCGGTCGAGCTGCCCGTCTACCCGTTCCAGCGGCAGCGGTACT<br>GGCTGCCCATCCCCACCGGCGGGCGCGCACGGGACGAGGACGACGACTGGCGCTACCAGGTCGTATGGCGGGAAGCCGAGTGGGAGAGCGCTTCGCTGGCCGGACGCGTGCTGCTGGTGACC<br>GGACCGGGCGTGCCGTCCGAGTTGTCGGACGCCATCCGAAGTGGACTGGAGCAGAGCGGTGCGACGGTCCTGACCTGCGACGTGGAATCCCGTTCGACCATCGGCACCGCACTGGAGGCCGC<br>CGACACCGACGCTCTGTCCACTGTGGTGTCGCTGCTGTCCCGCGACGGCGAGGCCGTCGATCCGTCGCTGGACGCGCTCGCCCTGGTCCAGGCCCTCGGAGCGGCCGGGGTCGAAGCACCGCT<br>GTGGGTGCTGACCCGCAACGCCGTGCAGGTGGCCGACGGCGAACTGGTCGATCCGGCGCAGGCCATGGTGGGCGGTCTCGGCCGCGTGGTCGGCATCGAGCAGCCGGGGCGCTGGGGCGGTC<br>TGGTGGACCTGGTCGACGCCGATGCCGCGTCGATCCGGTCGCTGGCCGCGGTGCTGGCGGACCCGCGCGGCGAGGAGCAGGTCGCGATCCGGGCGGACGGGATCAAGGTGGCGAGGCTCGTG<br>CCCGCCCCCGCCCGCGCCGCACGCACCCGCTGGAGCCCTCGCGGCACCGTGCTGGTCACCGGCGGCACCGGAGGGATCGGCGCGCACGTCGCCCGCTGGCTGGCCCGCTCGGGCGCCGAGCACC<br>TGGTGCTGCTGGGCAGGCGCGGTGCCGACGCACCCGGCGCGTCCGAGCTGAGGGAGGAGCTGACCGCGCTCGGCACGGGCGTGACCATCGCCGCCTGCGACGTCGCCGACCGGGCGCGGCTCG<br>AAGCGGTGCTCGCCGCGGAGCGCGCCGAGGGACGCACGGTCAGCGCCGTGATGCACGCGGCGGGGGTTTCCACGTCCACGCCCCTCGACGACCTCACCGAAGCCGAGTTCACCGAGATCGCCG<br>ACGTGAAGGTGCGCGGCACCGTCAACCTGGACGAGCTCTGCCCGGACCTCGACGCGTTCGTGTTGTTCTCCTCCAACGCGGGCGTGTGGGGCAGTCCGGGGCTCGCCTCCTACGCGGCGGCCA<br>ACGCCTTCCTCGACGGCTTCGCGCGGCGGCGCCGGAGCGAGGGCGCGCCGGTGACGTCCATCGCCTGGGGGCTCTGGGCCGGGCAGAACATGGCCGGGGACGAGGGCGGCGAGTACCTGCGC<br>AGCCAGGGCCTGCGGGCCATGGACCCGGATCGGGCCGTCGAGGAACTGCACATCACCCTCGACCACGGTCAGACGTCCGTGTCGGTCGTGGACATGGATCGCAGGCGGTTCGTCGAGCTGTT<br>CACCGCGGCCCGGCACCGGCCGCTGTTCGACGAGATCGCCGGTGCCCGGGCGGAAGCCCGGCAGAGCGAGGAGGGCCCGGCGCTCGCCCAGCGGCTCGCGGCGCTGTCGACGGCCGAGAGGCG<br>CGAGCACCTCGCCCACCTGATCCGCGCCGAGGTCGCCGCGGTGCTCGGCCACGGCGACGACGCGGCGATCGACCGCGACCGCGCCTTCCGCGACCTCGGCTTCGACTCCATGACCGCCGTCGAC<br>CTGCGGAACCGGCTCGCCGCGGTGACCGGGGTGCGGGAAGCCGCGACGGTGGTCTTCGACCACCCGACCATCACCCGGCTCGCCGACCACTACCTGGAGCGGCTCGTCGGCGCAGCAGAGGCG<br>GAGCAAGCCCCGGCGCTCGTGCGCGAGGTGCCGAAGGATGCCGACGACCCGATCGCGATCGTCGGCATGGCCTGCCGCTTCCCCGGCGGCGTGCACAACCCCGGTGAGCTGTGGGAGTTCATC<br>GTCGGCGGCGGAGACGCCGTGACGGAGATGCCCACCGACCGCGGCTGGGACCTCGACGCGCTGTTCGACCCCGACCCGCAGCGCCACGGAACCAGCTACTCGCGACACGGCGCGTTCCTCGAC<br>GGGGCCGCCGACTTCGACGCGGCGTTCTTCGGGATCTCGCCGCGCGAGGCGCTGGCGATGGACCCGCAGCAGCGCCAGGTCCTGGAAACGACGTGGGAGCTGTTCGAGAACGCCGGCATCGA<br>CCCGCACTCGCTGCGGGGCAGCGACACCGGCGTCTTCCTCGGCGCCGCGTACCAGGGCTACGGCCAGGACGCGGTGGTGCCCGAGGACAGCGAGGGCTACCTGCTCACCGGCAACTCCTCCGC<br>CGTGGTGTCCGGCCGGGTCGCCTACGTGCTGGGGCTGGAAGGCCCCGCGGTCACGGTGGACACGGCGTGTTCGTCGTCGTTGGTGGCCTTGCATTCGGCGTGTGGGTCGTTGCGTGACGGTG<br>ACTGCGGTCTTGCGGTGGCCGGTGGTGTGTCGGTGATGGCGGGCCCGGAGGTGTTCACCGAGTTCTCCCGCCAGGGCGGCTTGGCCGTGGACGGGCGCTGCAAGGCGTTCTCCGCGGAGGCC<br>GACGGCTTCGGTTTCGCCGAGGGCGTCGCGGTGGTCCTGCTCCAGCGGTTGTCCGACGCCCGCAGGGCGGGTCGCCAGGTGCTCGGCGTGGTCGCGGGCTCGGCGATCAACCAGGACGGCGCG<br>AGCAACGGTCTCGCGGCGCCGAGCGGCGTCGCCCAGCAGCGCGTGATCCGCAAGGCGTGGGCGCGTGCGGGGATCACGGGCGCGGATGTGGCCGTGGTGGAGGCGCATGGGACCGGTACGCG<br>GCTGGGCGATCCGGTGGAGGCGTCGGCGTTGCTGGCTACTTACGGCAAGTCGCGCGGGTCGTCGGGCCCGGTGCTGCTGGGTTCGGTGAAGTCGAACATCGGTCACGCGCAGGCGGCCGCGG<br>GTGTCGCGGGCGTGATCAAGGTGGTCCTGGGGTTGAACCGCGGCCTGGTGCCGCCGATGCTCTGCCGCGGCGAGCGGTCGCCGCTGATCGAATGGTCCTCGGGTGGTGTGGAACTTGCCGAG<br>GCCGTGAGCCCGTGGCCTCCGGCCGCGGACGGGGTGCGCCGGGCCGGTGTGTCGGCGTTCGGGGTGAGCGGGACGAACGCGCACGTGATCATCGCCGAGCCCCCGGAGCCCGAGCCGCTGCCG<br>GAACCCGGACCGGTGGGCGTGCTGGCCGCTGCGAACTCGGTGCCCGTACTGCTGTCGGCCAGGACCGAGACCGCGTTGGCAGCGCAGGCGCGGCTCCTGGAGTCCGCAGTGGACGACTCGGTT<br>CCGTTGACGGCATTGGCTTCCGCGCTGGCCACCGGACGCGCCCACCTGCCGCGTCGTGCGGCGTTGCTGGCAGGCGACCACGAACAGCTCCGCGGGCAGTTGCGAGCGGTCGCCGAGGGCGTT<br>GCGGCTCCCGGTGCCACCACCGGAACCGCCTCCGCCGGCGGCGTGGTTTTCGTCTTCCCAGGTCAGGGTGCTCAGTGGGAGGGCATGGCCCGGGGCTTGCTCTCGGTCCCCGTCTTCGCCGAG<br>TCGATCGCCGAGTGCGATGCGGTGTTGTCGGAGGTGGCCGGGTTCTCGGCCTCCGAAGTGCTGGAGCAGCGTCCGGACGCGCCGTCGCTGGAGCGGGTCGACGTCGTACAGCCGGTGTTGTT<br>CTCCGTGATGGTGTCGCTGGCGCGGCTGTGGGGCGCTTGCGGAGTCAGCCCCTCGGCCGTCATCGGCCATTCGCAGGGCGAGATCGCCGCCGCGGTGGTGGCCGGGGTGTTGTCGCTGGAGG

Construct	Sequence form start to stop codon
	ACGGCGTGCGCGTCGTGGCCCTGCGCGCGAAGGCGTTGCGTGCGCTGGCGGGCAAGGGCGGCATGGTCTCGTTGGCGGCTCCCGGTGAACGCGCCCGCGCGCTGATCGCACCGTGGGAGGACC<br>GGATCTCCGTCGCGGCGGTCAACTCCCCGTCCTCGGTCGTGGTCTCCGGCGATCCGGAGGCGCTGGCCGAACTCGTCGCACGTTGCGAGGACGAGGGCGTGCGCGCCAAGACGCTCCCGGTGG<br>ACTACGCCTCGCACTCCCGCCACGTCGAGGAGATCCGCGAGACGATCCTCGCCGACCTCGACGGCATCTCCGCGCGGCGTGCCGCCATCCCGCTCTACTCCACGCTGCACGGCGAACGGCGCG<br>ACGGCGCCGACATGGGTCCGCGGTACTGGTACGACAACCTGCGCTCCCAGGTGCGCTTCGACGAGGCGGTCTCGGCCGCCGTCGCCGACGGTCACGCCACCTTCGTCGAGATGAGCCCGCACC<br>CGGTGCTCACCGCGGCGGTGCAGGAGATCGCCGCGGACGCCGTGGCCATCGGGTCGCTGCACCGCGACACCGCGGAGGAGCACCTGATCGCCGAGCTCGCCCGGGCGCACGTGCACGGCGTGG<br>CCGTGGACTGGCGGAACGTCTTCCCGGCGGCACCTCCGGTGGCGCTGCCCAACTACCCGTTCGAGCCCCAGCGGTACTGGCTCGCGCCGGAGGTGTCCGACCAGCTCGCCGACAGCCGCTACC<br>GCGTCGACTGGCGACCGCTGGCCACCACGCCGGTGGACCTGGAAGGCGGCTTCCTGGTCCACGGGTCCGCACCGGAGTCGCTGACCAGCGCAGTCGAGAAGGCCGGAGGCCGCGTCGTGCCGG<br>TCGCCTCGGCCGACCGCGAAGCGCTCGCGGCGGCCCTGCGGGAGGTGCCGGGCGAGGTCGCCGGCGTGCTCTCGGTCCACACCGGCGCCGCAACGCACCTCGCCCTGCACCAGTCGCTGGGTG<br>AGGCCGGCGTGCGGGCCCCGCTCTGGCTGGTCACCAGCCGAGCGGTCGCGCTCGGGGAGTCCGAGCCGGTCGATCCCGAGCAGGCGATGGTGTGGGGTCTCGGGCGCGTCATGGGCCTGGAG<br>ACCCCGGAACGGTGGGGCGGTCTGGTGGACCTGCCCGCCGAACCCGCGCCGGGGGACGGCGAGGCGTTCGTCGCCTGCCTCGGCGCGGACGGCCACGAGGACCAGGTCGCGATCCGTGACCAC<br>GCCCGCTACGGCCGCCGCCTCGTCCGCGCCCCGCTGGGCACCCGCGAGTCGAGCTGGGAGCCGGCGGGCACGGCGCTGGTCACCGGCGGCACCGGTGCGCTCGGCGGCCACGTCGCCCGCCACC<br>TCGCCAGGTGCGGGGTGGAGGACCTGGTGCTGGTCAGCAGGCGCGGCGTCGACGCTCCCGGCGCGGCCGAGCTGGAAGCCGAACTGGTCGCCCTCGGCGCGAAGACGACCATCACCGCCTGCG<br>ACGTGGCCGACCGCGAGCAGCTCTCCAAGCTGCTGGAAGAACTGCGCGGGCAGGGACGTCCGGTGCGGACCGTCGTGCACACCGCCGGGGTGCCCGAATCGAGGCCGCTGCACGAGATCGGC<br>GAGCTGGAGTCGGTCTGCGCGGCGAAGGTGACCGGGGCCCGGCTGCTCGACGAGCTGTGCCCGGACGCCGAGACCTTCGTCCTGTTCTCGTCCGGAGCGGGGGTGTGGGGCAGTGCGAACCT<br>CGGCGCCTACTCCGCGGCCAACGCCTACCTCGACGCGCTGGCCCACCGCCGCCGTGCGGAAGGCCGTGCGGCGACGTCCGTCGCGTGGGGCGCCTGGGCGGGCGAGGGCATGGCCACCGGCGA<br>CCTCGAGGGGCTCACCCGGCGCGGCCTGCGCCCGATGGCGCCCGAGCGCGCGATCCGCGCGCTGCACCAGGCGCTGGACAACGGCGACACGTGCGTTTCGATCGCCGACGTCGACTGGGAGCG<br>CTTCGCGGTCGGCTTCACCGCCGCCCGGCCGCGTCCGCTGCTGGACGAGCTCGTCACGCCGGCGGTGGGGGCCGTCCCCGCGGTGCAGGCGGCCCCGGCGCGGGAGATGACGTCGCAGGAGTT<br>GCTGGAGTTCACGCACTCGCACGTCGCGGCGATCCTCGGGCATTCCAGCCCGGACGCGGTCGGGCAGGACCAGCCGTTCACCGAGCTCGGCTTCGACTCGCTGACCGCGGTCGGGCTGCGCAA<br>CCAGCTCCAGCAGGCCACCGGGCTCGCGCTGCCCGCGACCCTGGTGTTCGAGCACCCCACGGTCCGCAGGTTGGCCGACCACATAGGACAGCAGCTCGACAGCGGGACTCCCGCCCGGGAAGC<br>GAGCAGCGCTCTTCGCGACGGCTACCGGCAGGCGGGCGTGTCGGGCAGGGTCCGGTCCTACCTCGACCTGCTGGCGGGGCTGTCGGACTTCCGCGAGCACTTCGACGGCTCCGACGGGTTCTC<br>CCTCGATCTCGTGGACATGGCCGACGGTCCCGGAGAGGTCACGGTGATCTGCTGCGCGGGAACGGCGGCGATCTCCGGTCCGCACGAGTTCACCCGGCTCGCCGGGGCGCTGCGCGGAATCGC<br>TCCGGTTCGGGCCGTGCCCCAGCCCGGCTACGAGGAGGGCGAACCTCTGCCGTCGTCGATGGCGGCGGTGGCGGCGGTGCAGGCCGATGCGGTCATCAGGACACAGGGGGACAAGCCGTTCG<br>TGGTGGCCGGTCACTCCGCGGGGGCACTGATGGCCTACGCGCTGGCGACCGAACTGCTCGATCGCGGGCACCCGCCACGCGGTGTCGTCCTGATCGACGTCTACCCGCCCGGTCACCAGGACG<br>CGATGAACGCCTGGCTGGAGGAGCTGACCGCCACGCTGTTCGACCGCGAGACGGTGCGGATGGACGACACCAGGCTCACCGCCCTGGGCGCCTACGACCGCCTCACCGGTCAGTGGCGACCCC<br>GGGAAACCGGGCTGCCGACGCTGCTGGTCAGCGCCGGCGAGCCGATGGGTCCGTGGCCCGACGACAGCTGGAAGCCGACGTGGCCCTTCGAGCACGACACCGTCGCCGTCCCCGGCGACCACT<br>TCACGATGGTGCAGGAACACGCCGACGCGATCGCGCGGCACATCGACGCCTGGCTGGGCGGAGGGAATTCGCTCGAGCATCATCACCACCACCACCACCACTGA
pMJD147<br>(M5*-<br>M6*-TE)	MGSSGDNGMTEEKLRRYLKRTVTELDSVTARLREVEHRAGEPIAIVGMACRFPGDVDSPESFWEFVSGGGDAIAEAPADRGWEPDPDARLGGMLAAAGDFDAGFFGISPREALAMDPQQRI<br>MLEISWEALERAGHDPVSLRGSATGVFTGVGTVDYGPRPDEAPDEVLGYVGTGTASSVASGRVAYCLGLEGPAMTVDTACSSGLTALHLAMESLRRDECGLALAGGVTVMSSPGAFTEFRSQ<br>GGLAADGRCKPFSKAADGFGLAEGAGVLVLQRLSAARREGRPVLAVLRGSAVNQDGASNGLTAPSGPAQQRVIRRALENAGVRAGDVDYVEAHGTGTRLGDPIEVHALLSTYGAERDPDDPL<br>WIGSVKSNIGHTQAAAGVAGVMKAVLALRHGEMPRTLHFDEPSPQIEWDLGAVSVVSQARSWPAGERPRRAGVSSFGISGTNAHVIVEEAPEADEPEPAPDSGPVPLVLSGRDEQAMRAQAG<br>RLADHLAREPRNSLRDTGFTLATRRSAWEHRAVVVGDRDDALAGLRAVADGRIADRTATGQARTNKRPLWFICSGMGTQWRGMGLSLMRLDSFRESILRSDEAVKPLGVKVSDLLLSTDER<br>TFDDIVHAFVSLTAIQIALIDLLTSVGLKPDGIIGHSLGEVACGYADGCLSQREAVLAAYWRGQCIKDAHLPPGSMAAVGLSWEECKQRCPAGVVPACHNSEDTVTISGPQAAVNEFVEQLKQE<br>GVFAKEVRTGGLAFHSYFMEGIAPTLLQALKKVIREPRPRSARWLSTSIPEAQWQSSLARTSSAEYNVNNLVSPVLFQEALWHIPEHAVVLEIAPHALLQAVLKRGVKSSCTIIPLMKRDHKDN<br>LEFFLTNLGKVHLTGVEVDWSPAFADARPVELPVYPFQRQRYWLPIPTGGRARDEDDDWRYQVVWREAEWESASLAGRVLLVTGPGVPSELSDAIRSGLEQSGATVLTCDVESRSTIGTALE<br>AADTDALSTVVSLLSRDGEAVDPSLDALALVQALGAAGVEAPLWVLTRNAVQVADGELVDPAQAMVGGLGRVVGIEQPGRWGGLVDLVDADAASIRSLAAVLADPRGEEQVAIRADGIKVA<br>RLVPAPARAARTRWSPRGTVLVTGGTGGIGAHVARWLARSGAEHLVLLGRRGADAPGASELREELTALGTGVTIAACDVADRARLEAVLAAERAEGRTVSAVMHAAGVSTSTPLDDLTEAE<br>FTEIADVKVRGTVNLDELCPDLDAFVLFSSNAGVWGSPGLASYAAANAFLDGFARRRRSEGAPVTSIAWGLWAGQNMAGDEGGEYLRSQGLRAMDPDRAVEELHITLDHGQTSVSVVDMD<br>RRRFVELFTAARHRPLFDEIAGARAEARQSEEGPALAQRLAALSTAERREHLAHLIRAEVAAVLGHGDDAAIDRDRAFRDLGFDSMTAVDLRNRLAAVTGVREAATVVFDHPTITRLADHYL<br>ERLVGAAEAEQAPALVREVPKDADDPIAIVGMACRFPGGVHNPGELWEFIVGGGDAVTEMPTDRGWDLDALFDPDPQRHGTSYSRHGAFLDGAADFDAAFFGISPREALAMDPQQRQVLET<br>TWELFENAGIDPHSLRGSDTGVFLGAAYQGYGQDAVVPEDSEGYLLTGNSSAVVSGRVAYVLGLEGPAVTVDTACSSSLVALHSACGSLRDGDCGLAVAGGVSVMAGPEVFTEFSRQGGLAVD<br>GRCKAFSAEADGFGFAEGVAVVLLQRLSDARRAGRQVLGVVAGSAINQDGASNGLAAPSGVAQQRVIRKAWARAGITGADVAVVEAHGTGTRLGDPVEASALLATYGKSRGSSGPVLLGSVKS<br>NIGHAQAAAGVAGVIKVVLGLNRGLVPPMLCRGERSPLIEWSSGGVELAEAVSPWPPAADGVRRAGVSAFGVSGTNAHVIIAEPPEPEPLPEPGPVGVLAAANSVPVLLSARTETALAAQARLL<br>ESAVDDSVPLTALASALATGRAHLPRRAALLAGDHEQLRGQLRAVAEGVAAPGATTGTASNKRPLWFICSGMGTQWRGMGLSLMRLDSFRESILRSDEAVKPLGVKVSDLLLSTDERTFDDI<br>VHAFVSLTAIQIALIDLLTSVGLKPDGIIGHSLGEVACGYADGCLSQREAVLAAYWRGQCIKDAHLPPGSMAAVGLSWEECKQRCPAGVVPACHNSEDTVTISGPQAAVNEFVEQLKQEGVFAK<br>EVRTGGLAFHSYFMEGIAPTLLQALKKVIREPRPRSARWLSTSIPEAQWQSSLARTSSAEYNVNNLVSPVLFQEALWHIPEHAVVLEIAPHALLQAVLKRGVKSSCTIIPLMKRDHKDNLEFFL<br>TNLGKVHLTGVAVDWRNVFPAAPPVALPNYPFEPQRYWLAPEVSDQLADSRYRVDWRPLATTPVDLEGGFLVHGSAPESLTSAVEKAGGRVVPVASADREALAAALREVPGEVAGVLSVHT<br>GAATHLALHQSLGEAGVRAPLWLVTSRAVALGESEPVDPEQAMVWGLGRVMGLETPERWGGLVDLPAEPAPGDGEAFVACLGADGHEDQVAIRDHARYGRRLVRAPLGTRESSWEPAGT

Construct	Sequence form start to stop codon
	ALVTGGTGALGGHVARHLARCGVEDLVLVSRRGVDAPGAAELEAELVALGAKTTITACDVADREQLSKLLEELRGQGRPVRTVVHTAGVPESRPLHEIGELESVCAAKVTGARLLDELCPDAE<br>TFVLFSSGAGVWGSANLGAYSAANAYLDALAHRRRAEGRAATSVAWGAWAGEGMATGDLEGLTRRGLRPMAPERAIRALHQALDNGDTCVSIADVDWERFAVGFTAARPRPLLDELVTPA<br>VGAVPAVQAAPAREMTSQELLEFTHSHVAAILGHSSPDAVGQDQPFTELGFDSLTAVGLRNQLQQATGLALPATLVFEHPTVRRLADHIGQQLDSGTPAREASSALRDGYRQAGVSGRVRSYL<br>DLLAGLSDFREHFDGSDGFSLDLVDMADGPGEVTVICCAGTAAISGPHEFTRLAGALRGIAPVRAVPQPGYEEGEPLPSSMAAVAAVQADAVIRTQGDKPFVVAGHSAGALMAYALATELLDR<br>GHPPRGVVLIDVYPPGHQDAMNAWLEELTATLFDRETVRMDDTRLTALGAYDRLTGQWRPRETGLPTLLVSAGEPMGPWPDDSWKPTWPFEHDTVAVPGDHFTMVQEHADAIARHIDA<br>WLGGGNSLEHHHHHHHH
pMJD147<br>(M5*-<br>M6*-TE)	ATGGGATCCAGCGGTGACAACGGCATGACCGAGGAAAAGCTCCGGCGCTACCTCAAGCGCACCGTCACCGAGCTCGACTCGGTGACCGCGCGCCTGCGTGAAGTCGAGCACCGGGCCGGTGA<br>GCCGATCGCGATCGTCGGCATGGCGTGCCGGTTCCCCGGCGACGTGGACTCGCCGGAGTCGTTCTGGGAGTTCGTGTCCGGCGGCGGGGACGCCATCGCGGAGGCCCCCGCCGACCGCGGCTG<br>GGAGCCGGACCCCGACGCGCGGCTGGGCGGGATGCTCGCGGCCGCGGGCGACTTCGACGCGGGCTTCTTCGGGATCTCGCCGCGCGAGGCGCTGGCGATGGACCCGCAGCAGCGGATCATGCT<br>GGAGATCTCGTGGGAGGCGCTGGAGCGCGCCGGCCACGATCCGGTGTCCCTGCGCGGCAGCGCGACCGGGGTGTTCACCGGTGTCGGCACCGTGGACTACGGCCCGCGACCCGACGAGGCCCC<br>GGACGAGGTCCTGGGCTACGTCGGCACCGGCACCGCCTCCAGCGTCGCCTCCGGCCGGGTCGCCTACTGCCTGGGCCTGGAAGGCCCGGCGATGACCGTCGACACCGCCTGTTCCTCCGGGCT<br>CACCGCCCTGCACCTGGCGATGGAGTCGCTGCGCCGGGACGAGTGCGGCCTGGCGCTGGCCGGCGGCGTGACGGTGATGAGCAGTCCCGGGGCGTTCACCGAGTTCCGCAGCCAGGGCGGGCT<br>CGCCGCCGACGGCCGCTGCAAGCCGTTCTCGAAGGCCGCCGACGGGTTCGGCCTGGCCGAGGGTGCCGGGGTCCTGGTGCTGCAACGGCTGTCGGCCGCGCGGCGGGAGGGCAGACCGGTGCT<br>GGCCGTGCTGCGGGGCTCGGCGGTCAACCAGGACGGCGCCAGCAACGGGCTGACCGCGCCGAGCGGACCCGCGCAGCAGCGGGTCATCCGCCGGGCGCTGGAGAACGCCGGTGTCCGGGCGG<br>GCGACGTCGACTACGTGGAGGCCCACGGCACCGGCACCAGGCTGGGCGACCCCATCGAGGTGCACGCGCTGCTCTCGACCTACGGCGCGGAACGCGACCCGGACGATCCACTGTGGATCGGTT<br>CGGTCAAGTCCAACATCGGCCACACCCAGGCCGCCGCCGGCGTCGCCGGGGTGATGAAGGCGGTGCTGGCGCTGCGGCACGGCGAGATGCCGCGCACGCTGCACTTCGACGAGCCCTCGCCGC<br>AGATCGAGTGGGACCTGGGCGCGGTGTCGGTGGTGTCGCAGGCGCGGTCGTGGCCCGCCGGCGAGAGGCCCCGCAGGGCGGGCGTCTCCTCGTTCGGCATCAGCGGCACCAACGCGCACGTC<br>ATCGTCGAAGAGGCGCCCGAGGCCGACGAGCCCGAGCCGGCACCCGACTCGGGTCCGGTCCCGCTGGTGTTGTCCGGCCGCGACGAGCAGGCGATGCGGGCGCAGGCGGGACGGCTGGCAGA<br>CCACCTCGCCCGCGAGCCGCGGAACTCGTTGCGCGACACCGGTTTCACGCTGGCCACCCGCCGCAGCGCGTGGGAGCACCGCGCGGTGGTGGTCGGCGACCGCGACGACGCCCTCGCCGGGCT<br>GCGCGCGGTGGCCGACGGCCGCATCGCCGACCGGACGGCCACCGGGCAGGCCCGAACTAACAAGCGCCCACTCTGGTTCATCTGCTCAGGGATGGGCACGCAGTGGCGCGGGATGGGGCTGA<br>GCCTCATGCGCCTGGACAGCTTCCGTGAGTCTATCCTGCGCTCCGATGAGGCTGTGAAGCCGTTGGGAGTGAAAGTGTCAGATCTGCTGTTGAGCACAGATGAGCGCACCTTTGATGACATC<br>GTGCATGCCTTTGTGAGCCTCACTGCCATCCAGATTGCCCTCATCGACCTACTGACTTCTGTGGGACTGAAACCTGACGGCATCATTGGGCACTCCTTGGGAGAGGTTGCCTGTGGCTATGC<br>AGATGGCTGTCTCTCCCAGAGAGAGGCTGTGCTTGCAGCTTACTGGCGAGGCCAGTGCATCAAAGATGCCCACCTCCCGCCTGGATCCATGGCAGCTGTTGGTTTGTCCTGGGAGGAATGTA<br>AACAGCGCTGCCCCGCTGGCGTGGTGCCTGCCTGCCACAACTCTGAGGACACCGTGACCATCTCTGGACCTCAGGCTGCAGTGAATGAATTTGTGGAGCAGCTAAAGCAAGAAGGTGTGTTT<br>GCCAAGGAGGTACGAACAGGAGGCCTGGCTTTCCACTCCTACTTCATGGAAGGAATTGCCCCCACATTGCTGCAGGCTCTCAAGAAGGTGATCCGGGAACCACGGCCGCGCTCGGCTCGATG<br>GCTCAGCACCTCTATCCCTGAGGCCCAGTGGCAGAGCAGCCTGGCCCGCACATCTTCTGCCGAGTACAATGTCAACAACCTGGTGAGCCCTGTGCTCTTCCAGGAAGCACTGTGGCACATCCC<br>TGAGCATGCCGTGGTGCTGGAGATTGCGCCCCACGCACTGTTGCAGGCTGTCCTGAAGCGAGGCGTGAAGTCCAGCTGCACCATCATTCCCTTGATGAAGAGGGATCATAAAGATAACTTGG<br>AGTTCTTTCTCACCAACCTTGGCAAGGTGCACCTCACAGGCGTGGAGGTCGACTGGAGCCCCGCCTTCGCCGACGCGCGGCCGGTCGAGCTGCCCGTCTACCCGTTCCAGCGGCAGCGGTACT<br>GGCTGCCCATCCCCACCGGCGGGCGCGCACGGGACGAGGACGACGACTGGCGCTACCAGGTCGTATGGCGGGAAGCCGAGTGGGAGAGCGCTTCGCTGGCCGGACGCGTGCTGCTGGTGACC<br>GGACCGGGCGTGCCGTCCGAGTTGTCGGACGCCATCCGAAGTGGACTGGAGCAGAGCGGTGCGACGGTCCTGACCTGCGACGTGGAATCCCGTTCGACCATCGGCACCGCACTGGAGGCCGC<br>CGACACCGACGCTCTGTCCACTGTGGTGTCGCTGCTGTCCCGCGACGGCGAGGCCGTCGATCCGTCGCTGGACGCGCTCGCCCTGGTCCAGGCCCTCGGAGCGGCCGGGGTCGAAGCACCGCT<br>GTGGGTGCTGACCCGCAACGCCGTGCAGGTGGCCGACGGCGAACTGGTCGATCCGGCGCAGGCCATGGTGGGCGGTCTCGGCCGCGTGGTCGGCATCGAGCAGCCGGGGCGCTGGGGCGGTC<br>TGGTGGACCTGGTCGACGCCGATGCCGCGTCGATCCGGTCGCTGGCCGCGGTGCTGGCGGACCCGCGCGGCGAGGAGCAGGTCGCGATCCGGGCGGACGGGATCAAGGTGGCGAGGCTCGTG<br>CCCGCCCCCGCCCGCGCCGCACGCACCCGCTGGAGCCCTCGCGGCACCGTGCTGGTCACCGGCGGCACCGGAGGGATCGGCGCGCACGTCGCCCGCTGGCTGGCCCGCTCGGGCGCCGAGCACC<br>TGGTGCTGCTGGGCAGGCGCGGTGCCGACGCACCCGGCGCGTCCGAGCTGAGGGAGGAGCTGACCGCGCTCGGCACGGGCGTGACCATCGCCGCCTGCGACGTCGCCGACCGGGCGCGGCTCG<br>AAGCGGTGCTCGCCGCGGAGCGCGCCGAGGGACGCACGGTCAGCGCCGTGATGCACGCGGCGGGGGTTTCCACGTCCACGCCCCTCGACGACCTCACCGAAGCCGAGTTCACCGAGATCGCCG<br>ACGTGAAGGTGCGCGGCACCGTCAACCTGGACGAGCTCTGCCCGGACCTCGACGCGTTCGTGTTGTTCTCCTCCAACGCGGGCGTGTGGGGCAGTCCGGGGCTCGCCTCCTACGCGGCGGCCA<br>ACGCCTTCCTCGACGGCTTCGCGCGGCGGCGCCGGAGCGAGGGCGCGCCGGTGACGTCCATCGCCTGGGGGCTCTGGGCCGGGCAGAACATGGCCGGGGACGAGGGCGGCGAGTACCTGCGC<br>AGCCAGGGCCTGCGGGCCATGGACCCGGATCGGGCCGTCGAGGAACTGCACATCACCCTCGACCACGGTCAGACGTCCGTGTCGGTCGTGGACATGGATCGCAGGCGGTTCGTCGAGCTGTT<br>CACCGCGGCCCGGCACCGGCCGCTGTTCGACGAGATCGCCGGTGCCCGGGCGGAAGCCCGGCAGAGCGAGGAGGGCCCGGCGCTCGCCCAGCGGCTCGCGGCGCTGTCGACGGCCGAGAGGCG<br>CGAGCACCTCGCCCACCTGATCCGCGCCGAGGTCGCCGCGGTGCTCGGCCACGGCGACGACGCGGCGATCGACCGCGACCGCGCCTTCCGCGACCTCGGCTTCGACTCCATGACCGCCGTCGAC<br>CTGCGGAACCGGCTCGCCGCGGTGACCGGGGTGCGGGAAGCCGCGACGGTGGTCTTCGACCACCCGACCATCACCCGGCTCGCCGACCACTACCTGGAGCGGCTCGTCGGCGCAGCAGAGGCG<br>GAGCAAGCCCCGGCGCTCGTGCGCGAGGTGCCGAAGGATGCCGACGACCCGATCGCGATCGTCGGCATGGCCTGCCGCTTCCCCGGCGGCGTGCACAACCCCGGTGAGCTGTGGGAGTTCATC<br>GTCGGCGGCGGAGACGCCGTGACGGAGATGCCCACCGACCGCGGCTGGGACCTCGACGCGCTGTTCGACCCCGACCCGCAGCGCCACGGAACCAGCTACTCGCGACACGGCGCGTTCCTCGAC<br>GGGGCCGCCGACTTCGACGCGGCGTTCTTCGGGATCTCGCCGCGCGAGGCGCTGGCGATGGACCCGCAGCAGCGCCAGGTCCTGGAAACGACGTGGGAGCTGTTCGAGAACGCCGGCATCGA<br>CCCGCACTCGCTGCGGGGCAGCGACACCGGCGTCTTCCTCGGCGCCGCGTACCAGGGCTACGGCCAGGACGCGGTGGTGCCCGAGGACAGCGAGGGCTACCTGCTCACCGGCAACTCCTCCGC<br>CGTGGTGTCCGGCCGGGTCGCCTACGTGCTGGGGCTGGAAGGCCCCGCGGTCACGGTGGACACGGCGTGTTCGTCGTCGTTGGTGGCCTTGCATTCGGCGTGTGGGTCGTTGCGTGACGGTG

Construct	Sequence form start to stop codon
	ACTGCGGTCTTGCGGTGGCCGGTGGTGTGTCGGTGATGGCGGGCCCGGAGGTGTTCACCGAGTTCTCCCGCCAGGGCGGCTTGGCCGTGGACGGGCGCTGCAAGGCGTTCTCCGCGGAGGCC<br>GACGGCTTCGGTTTCGCCGAGGGCGTCGCGGTGGTCCTGCTCCAGCGGTTGTCCGACGCCCGCAGGGCGGGTCGCCAGGTGCTCGGCGTGGTCGCGGGCTCGGCGATCAACCAGGACGGCGCG<br>AGCAACGGTCTCGCGGCGCCGAGCGGCGTCGCCCAGCAGCGCGTGATCCGCAAGGCGTGGGCGCGTGCGGGGATCACGGGCGCGGATGTGGCCGTGGTGGAGGCGCATGGGACCGGTACGCG<br>GCTGGGCGATCCGGTGGAGGCGTCGGCGTTGCTGGCTACTTACGGCAAGTCGCGCGGGTCGTCGGGCCCGGTGCTGCTGGGTTCGGTGAAGTCGAACATCGGTCACGCGCAGGCGGCCGCGG<br>GTGTCGCGGGCGTGATCAAGGTGGTCCTGGGGTTGAACCGCGGCCTGGTGCCGCCGATGCTCTGCCGCGGCGAGCGGTCGCCGCTGATCGAATGGTCCTCGGGTGGTGTGGAACTTGCCGAG<br>GCCGTGAGCCCGTGGCCTCCGGCCGCGGACGGGGTGCGCCGGGCCGGTGTGTCGGCGTTCGGGGTGAGCGGGACGAACGCGCACGTGATCATCGCCGAGCCCCCGGAGCCCGAGCCGCTGCCG<br>GAACCCGGACCGGTGGGCGTGCTGGCCGCTGCGAACTCGGTGCCCGTACTGCTGTCGGCCAGGACCGAGACCGCGTTGGCAGCGCAGGCGCGGCTCCTGGAGTCCGCAGTGGACGACTCGGTT<br>CCGTTGACGGCATTGGCTTCCGCGCTGGCCACCGGACGCGCCCACCTGCCGCGTCGTGCGGCGTTGCTGGCAGGCGACCACGAACAGCTCCGCGGGCAGTTGCGAGCGGTCGCCGAGGGCGTT<br>GCGGCTCCCGGTGCCACCACCGGAACCGCCTCCAACAAGCGCCCACTCTGGTTCATCTGCTCAGGGATGGGCACGCAGTGGCGCGGGATGGGGCTGAGCCTCATGCGCCTGGACAGCTTCCGT<br>GAGTCTATCCTGCGCTCCGATGAGGCTGTGAAGCCGTTGGGAGTGAAAGTGTCAGATCTGCTGTTGAGCACAGATGAGCGCACCTTTGATGACATCGTGCATGCCTTTGTGAGCCTCACTGC<br>CATCCAGATTGCCCTCATCGACCTACTGACTTCTGTGGGACTGAAACCTGACGGCATCATTGGGCACTCCTTGGGAGAGGTTGCCTGTGGCTATGCAGATGGCTGTCTCTCCCAGAGAGAGG<br>CTGTGCTTGCAGCTTACTGGCGAGGCCAGTGCATCAAAGATGCCCACCTCCCGCCTGGATCCATGGCAGCTGTTGGTTTGTCCTGGGAGGAATGTAAACAGCGCTGCCCCGCTGGCGTGGTG<br>CCTGCCTGCCACAACTCTGAGGACACCGTGACCATCTCTGGACCTCAGGCTGCAGTGAATGAATTTGTGGAGCAGCTAAAGCAAGAAGGTGTGTTTGCCAAGGAGGTACGAACAGGAGGCCT<br>GGCTTTCCACTCCTACTTCATGGAAGGAATTGCCCCCACATTGCTGCAGGCTCTCAAGAAGGTGATCCGGGAACCACGGCCGCGCTCGGCTCGATGGCTCAGCACCTCTATCCCTGAGGCCCA<br>GTGGCAGAGCAGCCTGGCCCGCACATCTTCTGCCGAGTACAATGTCAACAACCTGGTGAGCCCTGTGCTCTTCCAGGAAGCACTGTGGCACATCCCTGAGCATGCCGTGGTGCTGGAGATTG<br>CGCCCCACGCACTGTTGCAGGCTGTCCTGAAGCGAGGCGTGAAGTCCAGCTGCACCATCATTCCCTTGATGAAGAGGGATCATAAAGATAACTTGGAGTTCTTTCTCACCAACCTTGGCAAG<br>GTGCACCTCACAGGCGTGGCCGTGGACTGGCGGAACGTCTTCCCGGCGGCACCTCCGGTGGCGCTGCCCAACTACCCGTTCGAGCCCCAGCGGTACTGGCTCGCGCCGGAGGTGTCCGACCAG<br>CTCGCCGACAGCCGCTACCGCGTCGACTGGCGACCGCTGGCCACCACGCCGGTGGACCTGGAAGGCGGCTTCCTGGTCCACGGGTCCGCACCGGAGTCGCTGACCAGCGCAGTCGAGAAGGCC<br>GGAGGCCGCGTCGTGCCGGTCGCCTCGGCCGACCGCGAAGCGCTCGCGGCGGCCCTGCGGGAGGTGCCGGGCGAGGTCGCCGGCGTGCTCTCGGTCCACACCGGCGCCGCAACGCACCTCGCC<br>CTGCACCAGTCGCTGGGTGAGGCCGGCGTGCGGGCCCCGCTCTGGCTGGTCACCAGCCGAGCGGTCGCGCTCGGGGAGTCCGAGCCGGTCGATCCCGAGCAGGCGATGGTGTGGGGTCTCGGG<br>CGCGTCATGGGCCTGGAGACCCCGGAACGGTGGGGCGGTCTGGTGGACCTGCCCGCCGAACCCGCGCCGGGGGACGGCGAGGCGTTCGTCGCCTGCCTCGGCGCGGACGGCCACGAGGACCAG<br>GTCGCGATCCGTGACCACGCCCGCTACGGCCGCCGCCTCGTCCGCGCCCCGCTGGGCACCCGCGAGTCGAGCTGGGAGCCGGCGGGCACGGCGCTGGTCACCGGCGGCACCGGTGCGCTCGGC<br>GGCCACGTCGCCCGCCACCTCGCCAGGTGCGGGGTGGAGGACCTGGTGCTGGTCAGCAGGCGCGGCGTCGACGCTCCCGGCGCGGCCGAGCTGGAAGCCGAACTGGTCGCCCTCGGCGCGAAG<br>ACGACCATCACCGCCTGCGACGTGGCCGACCGCGAGCAGCTCTCCAAGCTGCTGGAAGAACTGCGCGGGCAGGGACGTCCGGTGCGGACCGTCGTGCACACCGCCGGGGTGCCCGAATCGAG<br>GCCGCTGCACGAGATCGGCGAGCTGGAGTCGGTCTGCGCGGCGAAGGTGACCGGGGCCCGGCTGCTCGACGAGCTGTGCCCGGACGCCGAGACCTTCGTCCTGTTCTCGTCCGGAGCGGGGGT<br>GTGGGGCAGTGCGAACCTCGGCGCCTACTCCGCGGCCAACGCCTACCTCGACGCGCTGGCCCACCGCCGCCGTGCGGAAGGCCGTGCGGCGACGTCCGTCGCGTGGGGCGCCTGGGCGGGCGA<br>GGGCATGGCCACCGGCGACCTCGAGGGGCTCACCCGGCGCGGCCTGCGCCCGATGGCGCCCGAGCGCGCGATCCGCGCGCTGCACCAGGCGCTGGACAACGGCGACACGTGCGTTTCGATCGC<br>CGACGTCGACTGGGAGCGCTTCGCGGTCGGCTTCACCGCCGCCCGGCCGCGTCCGCTGCTGGACGAGCTCGTCACGCCGGCGGTGGGGGCCGTCCCCGCGGTGCAGGCGGCCCCGGCGCGGGA<br>GATGACGTCGCAGGAGTTGCTGGAGTTCACGCACTCGCACGTCGCGGCGATCCTCGGGCATTCCAGCCCGGACGCGGTCGGGCAGGACCAGCCGTTCACCGAGCTCGGCTTCGACTCGCTGAC<br>CGCGGTCGGGCTGCGCAACCAGCTCCAGCAGGCCACCGGGCTCGCGCTGCCCGCGACCCTGGTGTTCGAGCACCCCACGGTCCGCAGGTTGGCCGACCACATAGGACAGCAGCTCGACAGCGG<br>GACTCCCGCCCGGGAAGCGAGCAGCGCTCTTCGCGACGGCTACCGGCAGGCGGGCGTGTCGGGCAGGGTCCGGTCCTACCTCGACCTGCTGGCGGGGCTGTCGGACTTCCGCGAGCACTTCGA<br>CGGCTCCGACGGGTTCTCCCTCGATCTCGTGGACATGGCCGACGGTCCCGGAGAGGTCACGGTGATCTGCTGCGCGGGAACGGCGGCGATCTCCGGTCCGCACGAGTTCACCCGGCTCGCCGG<br>GGCGCTGCGCGGAATCGCTCCGGTTCGGGCCGTGCCCCAGCCCGGCTACGAGGAGGGCGAACCTCTGCCGTCGTCGATGGCGGCGGTGGCGGCGGTGCAGGCCGATGCGGTCATCAGGACAC<br>AGGGGGACAAGCCGTTCGTGGTGGCCGGTCACTCCGCGGGGGCACTGATGGCCTACGCGCTGGCGACCGAACTGCTCGATCGCGGGCACCCGCCACGCGGTGTCGTCCTGATCGACGTCTACC<br>CGCCCGGTCACCAGGACGCGATGAACGCCTGGCTGGAGGAGCTGACCGCCACGCTGTTCGACCGCGAGACGGTGCGGATGGACGACACCAGGCTCACCGCCCTGGGCGCCTACGACCGCCTCA<br>CCGGTCAGTGGCGACCCCGGGAAACCGGGCTGCCGACGCTGCTGGTCAGCGCCGGCGAGCCGATGGGTCCGTGGCCCGACGACAGCTGGAAGCCGACGTGGCCCTTCGAGCACGACACCGTCG<br>CCGTCCCCGGCGACCACTTCACGATGGTGCAGGAACACGCCGACGCGATCGCGCGGCACATCGACGCCTGGCTGGGCGGAGGGAATTCGCTCGAGCATCATCACCACCACCACCACCAC

Table S3. Plasmids used in this study and their origin

Plasmid	Origin
pLB062_H6-mFAS(MAT-ACP)-DEBS2(cDD)_kan	this study
pLB063_twinstrep_mFAS(MAT)-VemG(ACP0)-DEBS2(cDD)_H6_pET22b_carb	this study
pYZ002_Vem(DD-M2-TE)-H1_1-1-H8_pET22b_carb	this study
pYZ003_Vem(DD-M2-TE)-H1_1-2-H8_pET22b_carb	this study
pYZ004_Vem(DD-M2-TE)-H1_1-3-H8_pET22b_carb	this study
pYZ005_Vem(DD-M2-TE)-H1_2-1-H8_pET22b_carb	this study
pYZ006_Vem(DD-M2-TE)-H1_2-2-H8_pET22b_carb	this study
pYZ007_Vem(DD-M2-TE)-H1_2-3-H8_pET22b_carb	this study
pYZ008_Vem(DD-M2-TE)-H1_3-1-H8_pET22b_carb	this study
pYZ009_Vem(DD-M2-TE)-H1_3-2-H8_pET22b_carb	this study
pYZ014_VemH(DD M2-TE)-H2_1-1-H8_pET22b_carb	this study
pYZ015_VemH(DD M2-TE)-H2_1-2-H8_pET22b_carb	this study
pYZ016_VemH(DD M2-TE)-H2_1-3-H8_pET22b_carb	this study
pYZ017_VemH(DD M2-TE)-H2_2-1-H8_pET22b_carb	this study
pYZ018_VemH(DD M2-TE)-H2_2-2-H8_pET22b_carb	this study
pYZ019_VemH(DD M2-TE)-H2_2-3-H8_pET22b_carb	this study
pYZ020_VemH(DD M2-TE)-H2_3-1-H8_pET22b_carb	this study
pYZ021_VemH(DD M2-TE)-H2_3-2-H8_pET22b_carb	this study
pYZ022_VemH(DD M2-TE)-H2_3-3-H8_pET22b_carb	this study
pYZ023_VemH(DD M2-TE)-H2_4-1-H8_pET22b_carb	this study
pYZ024_VemH(DD M2-TE)-H2_4-2-H8_pET22b_carb	this study
pYZ025_VemH(DD M2-TE)-H2_4-3-H8_pET22b_carb	this study
pYZ026_VemH(DD M2-TE)-H3_1-1-H8_pET22b_carb	this study
pYZ027_VemH(DD M2-TE)-H3_1-2-H8_pET22b_carb	this study
pYZ028_VemH(DD M2-TE)-H3_1-3-H8_pET22b_carb	this study
pYZ029_VemH(DD M2-TE)-H3_2-1-H8_pET22b_carb	this study
pYZ030_VemH(DD M2-TE)-H3_2-2-H8_pET22b_carb	this study
pYZ031_VemH(DD M2-TE)-H3_2-3-H8_pET22b_carb	this study
pYZ032_VemH(DD M2-TE)-H3_3-1-H8_pET22b_carb	this study
pYZ033_VemH(DD M2-TE)-H3_3-2-H8_pET22b_carb	this study
pYZ034_VemH(DD M2-TE)-H3_3-3-H8_pET22b_carb	this study
pYZ035_VemH(DD M2-TE)-H3_4-1-H8_pET22b_carb	this study
pYZ036_VemH(DD M2-TE)-H3_4-2-H8_pET22b_carb	this study
pYZ038_twinstrep-VemG(M0-M1-DD)-H1-1-H8_pET22b_carb	this study
pYZ040_twinstrep-VemG(M0-M1-DD)-H1-3-H8_pET22b_carb	this study
pYZ041_twinstrep-VemG(M0-M1-DD)-H3-1-H8_pET22b_carb	this study
pYZ042_twinstrep-VemG(M0-M1-DD)-H2-2-H8_pET22b_carb	this study

pYZ043_twinstrep-VemG(M0-M1-DD)H3-3-H8_pET22b_carb	this study
pYZ044_twinstrep-VemG(M0-M1-DD)-H1-1-H8_pET22b_carb	this study
pYZ045_twinstrep-VemG(M0-M1-DD)-H1-1-H8_pET22b_carb	this study
pYZ046_DEBS3(nDD)_VemM1(KS1-LD1)_mFAS(MAT)_VemM1(LD2-ACP-DD)-H6_pET22b_carb	this study
pLB047_twinstrep_VemG(M0)-DEBS(DD4)-H6_pET22b_carb	Buyachuihan et al.[1]
pCS008_DEBS3(nDD)-VemG(M1-DD)-H6_pET22b_car	Buyachuihan et al.[1]
pCS005_VemH(DD2-M2-TE)-H8_pET22b_carb	Buyachuihan et al.[1]
pAR357_SFP_pCDF-1b	Rittner et al.[4]
pSR008_StrepI_PikAIII_M5_PikAIV_TE_H8_pET22b	this study
pSR015_StrepI_PikAIII_M5_mFAS_MAT_H1_PikAIV_TE_H8_pET22b	this study
pAR268_DEBS3_H8_pET22b	this study
pSR022_StrepII_DEBS3_M5_H1_M6_WT_H8_pET22b	this study
pSR024_ StrepII_DEBS3_M5_H1_M6_H1_H8_pET22b	this study
pMJD105_DEBS3_M6_H1_H8_pET22b	this study

Table S4. List of all constructs that were cloned and tested for expression in *E. coli*. The constructs are sorted based on their hybrid group (H1-H3). Boundaries are categorized into upstream (usXX) and downstream (dsXX) of the transferase domain according to Englund et al.[7] MSAs for VemG- and VemH-based constructs can be found in Figure S6 and Figure S5, respectively. Constructs in grey were cloned, but not tested for expression.

	**Acceptor module VemG M1**		**Acceptor module VemH M2**	
Hybrid Group	Construct	Boundary	Construct	Boundary
H1	**pYZ038**	us106(mFAS sequence, us107 of VemG sequence)/ds26	pYZ002	us106/ds21
	pYZ044	us107/ds26	pYZ003	us106/ds25
	pYZ045	us106/ds26	pYZ004	us106/ds27
			pYZ005	us108ds21
			pYZ006	us108/ds25
			pYZ007	us108/ds27
			pYZ008	us115/ds21
			pYZ009	us115/ds25
H2	pYZ040	us13/ds43	pYZ014	us17/ds37
	pYZ041	us1/ds41	pYZ015	us17/ds40
	pYZ042	us4/ds42	pYZ016	us17/ds43
			pYZ017	us23/ds37
			pYZ018	us23/ds40
			pYZ019	us23/ds43
			pYZ020	us25/ds37

			pYZ021	us25/ds40
			pYZ022	us25/ds43
			pYZ023	us27/ds37
			pYZ024	us27/ds40
			pYZ025	us27/ds43
H3	pYZ043	us17/ds25	pYZ026	Us17/ds21
			pYZ027	us17/ds25
			pYZ028	us17/ds27
			pYZ029	us23/ds21
			pYZ030	us23/ds25
			pYZ031	us23/ds27
			pYZ032	us25/ds21
			pYZ033	us25/ds25
			pYZ034	us25/ds27
			pYZ035	us27/ds21
			pYZ036	us27/ds25

Table S5. Amino acid sequences of MAT-swapped VEMS loading modules H1M0 and H2M0.

Construct	Amino acid sequence
LB062 (H1M0)	MGSSHHHHHHSSGSTNKRPLWFICSGMGTQWRGMGLSLMRLDSFRESILRSDEAVKPLGVKVSDLLLSTDERTFDDIVHAFVSLTAIQIALIDLLTSVGLKPDGIIGHSLGEVACGYADGCLSQREAVLAAYWRGQCIKDAHLPPGSMAAVGLSWEECKQRCPAGVVPACHNSEDTVTISGPQAAVNEFVEQLKQEGVFAKEVRTGGLAFHSYFMEGIAPTLLQALKKVIREPRPRSARWLSTSIPEAQWQSSLARTSSAEYNVNNLVSPVLFQEALWHIPEHAVVLEIAPHALLQAVLKRGVKSSCTIIPLMKRDHKDNLEFFLTNLGKVHLTGIGGGSGGGSAGGSGDGDTQRDLVKAVAHILGIRDLAGINLDSTLADLGLDSLMGVEVRQILEREHDLVLPMREVRQLTLRKLQEMSSGTEVAQREFAASPAVDIGDRLDELEKALEALSAEDGHDDVGQRLESLLRRWNSRRADAPSTSAISEDASDDELFSMLDQRFGGGEDL*
LB063 (H2M0)	MSAWSHPQFEKGGGSGGGSGGSAWSHPQFEKGAGSNKRPLWFICSGMGTQWRGMGLSLMRLDSFRESILRSDEAVKPLGVKVSDLLLSTDERTFDDIVHAFVSLTAIQIALIDLLTSVGLKPDGIIGHSLGEVACGYADGCLSQREAVLAAYWRGQCIKDAHLPPGSMAAVGLSWEECKQRCPAGVVPACHNSEDTVTISGPQAAVNEFVEQLKQEGVFAKEVRTGGLAFHSYFMEGIAPTLLQALKKVIREPRPRSARWLSTSIPEAQWQSSLARTSSAEYNVNNLVSPVLFQEALWHIPEHAVVLEIAPHALLQAVLKRGVKSSCTIIPLMKRDHKDNLEFFLTNLGKVHLTGIGGGSGGGSAGGSLRERLGVLPGRARQRALTALVREEAAGVLGLDAPRRIDAGLAFTRLGLTSLTAVALRDRLAARTGLRLPVTLAFDHPTPAAVAAVLDGELFAASPAVDIGDRLDELEKALEALSAEDGHDDVGQRLESLLRRWNSRRADAPSTSAISEDASDDELFSMLDQRFGGGEDLPNSSSVDKLAAALEHHHHHH*

Table S6. Amino acid sequences of MAT-swapped VEMS M2*-TE constructs.

Construct	Amino acid sequence
YZ002	MTGTEEKLVDYLKKVTAELQETRRQLRGALAASREPIAIVGMACRYPGGVRTPEALWRLVLDEQDAISGFPTNRGWDIDGIYHPDPDR<br>PGTCYAREGGFLHDAALFDAEFFGVSPREAQAMDPQQRLLLETAWEAFERAGIDPTSLRGSDTGVFAGVVHHDYATARVPETLEPYLV<br>TGLSGGVASGRIAYTFGFEGPAVTVDTACSSSLVALHQAAHALRSGECELALAGGVTIMATPRAFLSFSRQRGLSPDGRCRAFGAGADG<br>TGWAEGAGMLLVERLSDARRKGHPVLAVLRGSAVNQDGASNGLSAPNGPSQQRVIRKALAHAGLAARDVDVVEGHGTGTKLGDPIEA<br>QALLATYGQERDAGLPLHLGSMKSNVGHSQAAAGVGGVIKMIEAMRHGILPRTLHADEPTPHVDWSAGDIELLTRRRAWPETGRPRR<br>AAVSSFGISGTNAHVILEAPPEEPARDAAETRPEARSREAAEGRREAAGQGRTETGPGPSATPPEAPGRARPPVPWPLSGRDAGALRD<br>QIGRLRAHLDAAPADPEDVAHSLARRAVFRHRAVLLAAPQAPAGGSPRAVTGVARKRPLWFICSGMGTQWRGMGLSLMRLDSFRESI<br>LRSDEAVKPLGVKVSDLLLSTDERTFDDIVHAFVSLTAIQIALIDLLTSVGLKPDGIIGHSLGEVACGYADGCLSQREAVLAAYWRGQCIK<br>DAHLPPGSMAAVGLSWEECKQRCPAGVVPACHNSEDTVTISGPQAAVNEFVEQLKQEGVFAKEVRTGGLAFHSYFMEGIAPTLLQALK<br>KVIREPRPRSARWLSTSIPEAQWQSSLARTSSAEYNVNNLVSPVLFQEALWHIPEHAVVLEIAPHALLQAVLKRGVKSSCTIIPLMKRD<br>HKDNLEFFLTNLGKVHLTGGTVDWSGLCAGGRLVDLPTYPFQRQHYWIEDQPLPPTAPRPGTAPSGTGTAAEGAAAAEVPLSERLAR<br>LTGAERLAAVRELVLAEASETLGHTGTLITADRTRQELGFDSLTAIELRNRISRTLGVRLPPTLVFDHEDLGEIASFVDARLDDAATGRS<br>TGHGPLGEDGSGLLTELFREAAAAGRLDDAVTLTEAAARMRRTFTDAEDPAVRRTPVWFGRGPARPTVVCLPSFSAIAGVHVYARFA<br>DAFGDGWRVAALAHPGFVPGEPLPDSVDVLAELHARTVLDTVGADPFLLVGRSAGGWVAHEVAAVLERMGRAPDGVALLDTPARAD<br>DPRGHAVMVGGMLERDSRLVTIDDYRLTAMGGYSRLFREWKPEPIAAATLLVHAATPYGADEARIASWDLPHQAVKVTGDHFTMLE<br>RHSATTAEAVEQWSRSLLKLAAALEHHHHHHHH*
YZ003	MTGTEEKLVDYLKKVTAELQETRRQLRGALAASREPIAIVGMACRYPGGVRTPEALWRLVLDEQDAISGFPTNRGWDIDGIYHPDPDR<br>PGTCYAREGGFLHDAALFDAEFFGVSPREAQAMDPQQRLLLETAWEAFERAGIDPTSLRGSDTGVFAGVVHHDYATARVPETLEPYLV<br>TGLSGGVASGRIAYTFGFEGPAVTVDTACSSSLVALHQAAHALRSGECELALAGGVTIMATPRAFLSFSRQRGLSPDGRCRAFGAGADG<br>TGWAEGAGMLLVERLSDARRKGHPVLAVLRGSAVNQDGASNGLSAPNGPSQQRVIRKALAHAGLAARDVDVVEGHGTGTKLGDPIEA<br>QALLATYGQERDAGLPLHLGSMKSNVGHSQAAAGVGGVIKMIEAMRHGILPRTLHADEPTPHVDWSAGDIELLTRRRAWPETGRPRR<br>AAVSSFGISGTNAHVILEAPPEEPARDAAETRPEARSREAAEGRREAAGQGRTETGPGPSATPPEAPGRARPPVPWPLSGRDAGALRD<br>QIGRLRAHLDAAPADPEDVAHSLARRAVFRHRAVLLAAPQAPAGGSPRAVTGVARKRPLWFICSGMGTQWRGMGLSLMRLDSFRESI<br>LRSDEAVKPLGVKVSDLLLSTDERTFDDIVHAFVSLTAIQIALIDLLTSVGLKPDGIIGHSLGEVACGYADGCLSQREAVLAAYWRGQCIK<br>DAHLPPGSMAAVGLSWEECKQRCPAGVVPACHNSEDTVTISGPQAAVNEFVEQLKQEGVFAKEVRTGGLAFHSYFMEGIAPTLLQALK<br>KVIREPRPRSARWLSTSIPEAQWQSSLARTSSAEYNVNNLVSPVLFQEALWHIPEHAVVLEIAPHALLQAVLKRGVKSSCTIIPLMKRD<br>HKDNLEFFLTNLGKVHLDGGTVDWSGLCAGGRLVDLPTYPFQRQHYWIEDQPLPPTAPRPGTAPSGTGTAAEGAAAAEVPLSERLAR<br>LTGAERLAAVRELVLAEASETLGHTGTLITADRTRQELGFDSLTAIELRNRISRTLGVRLPPTLVFDHEDLGEIASFVDARLDDAATGRS<br>TGHGPLGEDGSGLLTELFREAAAAGRLDDAVTLTEAAARMRRTFTDAEDPAVRRTPVWFGRGPARPTVVCLPSFSAIAGVHVYARFA<br>DAFGDGWRVAALAHPGFVPGEPLPDSVDVLAELHARTVLDTVGADPFLLVGRSAGGWVAHEVAAVLERMGRAPDGVALLDTPARAD<br>DPRGHAVMVGGMLERDSRLVTIDDYRLTAMGGYSRLFREWKPEPIAAATLLVHAATPYGADEARIASWDLPHQAVKVTGDHFTMLE<br>RHSATTAEAVEQWSRSLLKLAAALEHHHHHHHH*
YZ004	MTGTEEKLVDYLKKVTAELQETRRQLRGALAASREPIAIVGMACRYPGGVRTPEALWRLVLDEQDAISGFPTNRGWDIDGIYHPDPDR<br>PGTCYAREGGFLHDAALFDAEFFGVSPREAQAMDPQQRLLLETAWEAFERAGIDPTSLRGSDTGVFAGVVHHDYATARVPETLEPYLV<br>TGLSGGVASGRIAYTFGFEGPAVTVDTACSSSLVALHQAAHALRSGECELALAGGVTIMATPRAFLSFSRQRGLSPDGRCRAFGAGADG<br>TGWAEGAGMLLVERLSDARRKGHPVLAVLRGSAVNQDGASNGLSAPNGPSQQRVIRKALAHAGLAARDVDVVEGHGTGTKLGDPIEA<br>QALLATYGQERDAGLPLHLGSMKSNVGHSQAAAGVGGVIKMIEAMRHGILPRTLHADEPTPHVDWSAGDIELLTRRRAWPETGRPRR<br>AAVSSFGISGTNAHVILEAPPEEPARDAAETRPEARSREAAEGRREAAGQGRTETGPGPSATPPEAPGRARPPVPWPLSGRDAGALRD<br>QIGRLRAHLDAAPADPEDVAHSLARRAVFRHRAVLLAAPQAPAGGSPRAVTGVARKRPLWFICSGMGTQWRGMGLSLMRLDSFRESI<br>LRSDEAVKPLGVKVSDLLLSTDERTFDDIVHAFVSLTAIQIALIDLLTSVGLKPDGIIGHSLGEVACGYADGCLSQREAVLAAYWRGQCIK<br>DAHLPPGSMAAVGLSWEECKQRCPAGVVPACHNSEDTVTISGPQAAVNEFVEQLKQEGVFAKEVRTGGLAFHSYFMEGIAPTLLQALK<br>KVIREPRPRSARWLSTSIPEAQWQSSLARTSSAEYNVNNLVSPVLFQEALWHIPEHAVVLEIAPHALLQAVLKRGVKSSCTIIPLMKRD<br>HKDNLEFFLTNLGQVHVDGGTVDWSGLCAGGRLVDLPTYPFQRQHYWIEDQPLPPTAPRPGTAPSGTGTAAEGAAAAEVPLSERLAR<br>LTGAERLAAVRELVLAEASETLGHTGTLITADRTRQELGFDSLTAIELRNRISRTLGVRLPPTLVFDHEDLGEIASFVDARLDDAATGRS<br>TGHGPLGEDGSGLLTELFREAAAAGRLDDAVTLTEAAARMRRTFTDAEDPAVRRTPVWFGRGPARPTVVCLPSFSAIAGVHVYARFA<br>DAFGDGWRVAALAHPGFVPGEPLPDSVDVLAELHARTVLDTVGADPFLLVGRSAGGWVAHEVAAVLERMGRAPDGVALLDTPARAD<br>DPRGHAVMVGGMLERDSRLVTIDDYRLTAMGGYSRLFREWKPEPIAAATLLVHAATPYGADEARIASWDLPHQAVKVTGDHFTMLE<br>RHSATTAEAVEQWSRSLLKLAAALEHHHHHHHH*

Construct	Amino acid sequence
YZ005	MTGTEEKLVDYLKKVTAELQETRRQLRGALAASREPIAIVGMACRYPGGVRTPEALWRLVLDEQDAISGFPTNRGWDIDGIYHPDPDR<br>PGTCYAREGGFLHDAALFDAEFFGVSPREAQAMDPQQRLLLETAWEAFERAGIDPTSLRGSDTGVFAGVVHHDYATARVPETLEPYLV<br>TGLSGGVASGRIAYTFGFEGPAVTVDTACSSSLVALHQAAHALRSGECELALAGGVTIMATPRAFLSFSRQRGLSPDGRCRAFGAGADG<br>TGWAEGAGMLLVERLSDARRKGHPVLAVLRGSAVNQDGASNGLSAPNGPSQQRVIRKALAHAGLAARDVDVVEGHGTGTKLGDPIEA<br>QALLATYGQERDAGLPLHLGSMKSNVGHSQAAAGVGGVIKMIEAMRHGILPRTLHADEPTPHVDWSAGDIELLTRRRAWPETGRPRR<br>AAVSSFGISGTNAHVILEAPPEEPARDAAETRPEARSREAAEGRREAAGQGRTETGPGPSATPPEAPGRARPPVPWPLSGRDAGALRD<br>QIGRLRAHLDAAPADPEDVAHSLARRAVFRHRAVLLAAPQAPAGGSPRAVTGVARPGPLWFICSGMGTQWRGMGLSLMRLDSFRESI<br>LRSDEAVKPLGVKVSDLLLSTDERTFDDIVHAFVSLTAIQIALIDLLTSVGLKPDGIIGHSLGEVACGYADGCLSQREAVLAAYWRGQCIK<br>DAHLPPGSMAAVGLSWEECKQRCPAGVVPACHNSEDTVTISGPQAAVNEFVEQLKQEGVFAKEVRTGGLAFHSYFMEGIAPTLLQALK<br>KVIREPRPRSARWLSTSIPEAQWQSSLARTSSAEYNVNNLVSPVLFQEALWHIPEHAVVLEIAPHALLQAVLKRGVKSSCTIIPLMKRD<br>HKDNLEFFLTNLGKVHLTGGTVDWSGLCAGGRLVDLPTYPFQRQHYWIEDQPLPPTAPRPGTAPSGTGTAAEGAAAAEVPLSERLAR<br>LTGAERLAAVRELVLAEASETLGHTGTLITADRTRQELGFDSLTAIELRNRISRTLGVRLPPTLVFDHEDLGEIASFVDARLDDAATGRS<br>TGHGPLGEDGSGLLTELFREAAAAGRLDDAVTLTEAAARMRRTFTDAEDPAVRRTPVWFGRGPARPTVVCLPSFSAIAGVHVYARFA<br>DAFGDGWRVAALAHPGFVPGEPLPDSVDVLAELHARTVLDTVGADPFLLVGRSAGGWVAHEVAAVLERMGRAPDGVALLDTPARAD<br>DPRGHAVMVGGMLERDSRLVTIDDYRLTAMGGYSRLFREWKPEPIAAATLLVHAATPYGADEARIASWDLPHQAVKVTGDHFTMLE<br>RHSATTAEAVEQWSRSLLKLAAALEHHHHHHHH*
YZ006	MTGTEEKLVDYLKKVTAELQETRRQLRGALAASREPIAIVGMACRYPGGVRTPEALWRLVLDEQDAISGFPTNRGWDIDGIYHPDPDR<br>PGTCYAREGGFLHDAALFDAEFFGVSPREAQAMDPQQRLLLETAWEAFERAGIDPTSLRGSDTGVFAGVVHHDYATARVPETLEPYLV<br>TGLSGGVASGRIAYTFGFEGPAVTVDTACSSSLVALHQAAHALRSGECELALAGGVTIMATPRAFLSFSRQRGLSPDGRCRAFGAGADG<br>TGWAEGAGMLLVERLSDARRKGHPVLAVLRGSAVNQDGASNGLSAPNGPSQQRVIRKALAHAGLAARDVDVVEGHGTGTKLGDPIEA<br>QALLATYGQERDAGLPLHLGSMKSNVGHSQAAAGVGGVIKMIEAMRHGILPRTLHADEPTPHVDWSAGDIELLTRRRAWPETGRPRR<br>AAVSSFGISGTNAHVILEAPPEEPARDAAETRPEARSREAAEGRREAAGQGRTETGPGPSATPPEAPGRARPPVPWPLSGRDAGALRD<br>QIGRLRAHLDAAPADPEDVAHSLARRAVFRHRAVLLAAPQAPAGGSPRAVTGVARPGPLWFICSGMGTQWRGMGLSLMRLDSFRESI<br>LRSDEAVKPLGVKVSDLLLSTDERTFDDIVHAFVSLTAIQIALIDLLTSVGLKPDGIIGHSLGEVACGYADGCLSQREAVLAAYWRGQCIK<br>DAHLPPGSMAAVGLSWEECKQRCPAGVVPACHNSEDTVTISGPQAAVNEFVEQLKQEGVFAKEVRTGGLAFHSYFMEGIAPTLLQALK<br>KVIREPRPRSARWLSTSIPEAQWQSSLARTSSAEYNVNNLVSPVLFQEALWHIPEHAVVLEIAPHALLQAVLKRGVKSSCTIIPLMKRD<br>HKDNLEFFLTNLGKVHLDGGTVDWSGLCAGGRLVDLPTYPFQRQHYWIEDQPLPPTAPRPGTAPSGTGTAAEGAAAAEVPLSERLAR<br>LTGAERLAAVRELVLAEASETLGHTGTLITADRTRQELGFDSLTAIELRNRISRTLGVRLPPTLVFDHEDLGEIASFVDARLDDAATGRS<br>TGHGPLGEDGSGLLTELFREAAAAGRLDDAVTLTEAAARMRRTFTDAEDPAVRRTPVWFGRGPARPTVVCLPSFSAIAGVHVYARFA<br>DAFGDGWRVAALAHPGFVPGEPLPDSVDVLAELHARTVLDTVGADPFLLVGRSAGGWVAHEVAAVLERMGRAPDGVALLDTPARAD<br>DPRGHAVMVGGMLERDSRLVTIDDYRLTAMGGYSRLFREWKPEPIAAATLLVHAATPYGADEARIASWDLPHQAVKVTGDHFTMLE<br>RHSATTAEAVEQWSRSLLKLAAALEHHHHHHHH*
YZ007	MTGTEEKLVDYLKKVTAELQETRRQLRGALAASREPIAIVGMACRYPGGVRTPEALWRLVLDEQDAISGFPTNRGWDIDGIYHPDPDR<br>PGTCYAREGGFLHDAALFDAEFFGVSPREAQAMDPQQRLLLETAWEAFERAGIDPTSLRGSDTGVFAGVVHHDYATARVPETLEPYLV<br>TGLSGGVASGRIAYTFGFEGPAVTVDTACSSSLVALHQAAHALRSGECELALAGGVTIMATPRAFLSFSRQRGLSPDGRCRAFGAGADG<br>TGWAEGAGMLLVERLSDARRKGHPVLAVLRGSAVNQDGASNGLSAPNGPSQQRVIRKALAHAGLAARDVDVVEGHGTGTKLGDPIEA<br>QALLATYGQERDAGLPLHLGSMKSNVGHSQAAAGVGGVIKMIEAMRHGILPRTLHADEPTPHVDWSAGDIELLTRRRAWPETGRPRR<br>AAVSSFGISGTNAHVILEAPPEEPARDAAETRPEARSREAAEGRREAAGQGRTETGPGPSATPPEAPGRARPPVPWPLSGRDAGALRD<br>QIGRLRAHLDAAPADPEDVAHSLARRAVFRHRAVLLAAPQAPAGGSPRAVTGVARPGPLWFICSGMGTQWRGMGLSLMRLDSFRESI<br>LRSDEAVKPLGVKVSDLLLSTDERTFDDIVHAFVSLTAIQIALIDLLTSVGLKPDGIIGHSLGEVACGYADGCLSQREAVLAAYWRGQCIK<br>DAHLPPGSMAAVGLSWEECKQRCPAGVVPACHNSEDTVTISGPQAAVNEFVEQLKQEGVFAKEVRTGGLAFHSYFMEGIAPTLLQALK<br>KVIREPRPRSARWLSTSIPEAQWQSSLARTSSAEYNVNNLVSPVLFQEALWHIPEHAVVLEIAPHALLQAVLKRGVKSSCTIIPLMKRD<br>HKDNLEFFLTNLGQVHVDGGTVDWSGLCAGGRLVDLPTYPFQRQHYWIEDQPLPPTAPRPGTAPSGTGTAAEGAAAAEVPLSERLAR<br>LTGAERLAAVRELVLAEASETLGHTGTLITADRTRQELGFDSLTAIELRNRISRTLGVRLPPTLVFDHEDLGEIASFVDARLDDAATGRS<br>TGHGPLGEDGSGLLTELFREAAAAGRLDDAVTLTEAAARMRRTFTDAEDPAVRRTPVWFGRGPARPTVVCLPSFSAIAGVHVYARFA<br>DAFGDGWRVAALAHPGFVPGEPLPDSVDVLAELHARTVLDTVGADPFLLVGRSAGGWVAHEVAAVLERMGRAPDGVALLDTPARAD<br>DPRGHAVMVGGMLERDSRLVTIDDYRLTAMGGYSRLFREWKPEPIAAATLLVHAATPYGADEARIASWDLPHQAVKVTGDHFTMLE<br>RHSATTAEAVEQWSRSLLKLAAALEHHHHHHHH*

Construct	Amino acid sequence
YZ008	MTGTEEKLVDYLKKVTAELQETRRQLRGALAASREPIAIVGMACRYPGGVRTPEALWRLVLDEQDAISGFPTNRGWDIDGIYHPDPDR<br>PGTCYAREGGFLHDAALFDAEFFGVSPREAQAMDPQQRLLLETAWEAFERAGIDPTSLRGSDTGVFAGVVHHDYATARVPETLEPYLV<br>TGLSGGVASGRIAYTFGFEGPAVTVDTACSSSLVALHQAAHALRSGECELALAGGVTIMATPRAFLSFSRQRGLSPDGRCRAFGAGADG<br>TGWAEGAGMLLVERLSDARRKGHPVLAVLRGSAVNQDGASNGLSAPNGPSQQRVIRKALAHAGLAARDVDVVEGHGTGTKLGDPIEA<br>QALLATYGQERDAGLPLHLGSMKSNVGHSQAAAGVGGVIKMIEAMRHGILPRTLHADEPTPHVDWSAGDIELLTRRRAWPETGRPRR<br>AAVSSFGISGTNAHVILEAPPEEPARDAAETRPEARSREAAEGRREAAGQGRTETGPGPSATPPEAPGRARPPVPWPLSGRDAGALRD<br>QIGRLRAHLDAAPADPEDVAHSLARRAVFRHRAVLLAAPQAPAGGSPRAVTGVARPGGTALLFSGMGTQWRGMGLSLMRLDSFRESI<br>LRSDEAVKPLGVKVSDLLLSTDERTFDDIVHAFVSLTAIQIALIDLLTSVGLKPDGIIGHSLGEVACGYADGCLSQREAVLAAYWRGQCIK<br>DAHLPPGSMAAVGLSWEECKQRCPAGVVPACHNSEDTVTISGPQAAVNEFVEQLKQEGVFAKEVRTGGLAFHSYFMEGIAPTLLQALK<br>KVIREPRPRSARWLSTSIPEAQWQSSLARTSSAEYNVNNLVSPVLFQEALWHIPEHAVVLEIAPHALLQAVLKRGVKSSCTIIPLMKRD<br>HKDNLEFFLTNLGKVHLTGGTVDWSGLCAGGRLVDLPTYPFQRQHYWIEDQPLPPTAPRPGTAPSGTGTAAEGAAAAEVPLSERLAR<br>LTGAERLAAVRELVLAEASETLGHTGTLITADRTRQELGFDSLTAIELRNRISRTLGVRLPPTLVFDHEDLGEIASFVDARLDDAATGRS<br>TGHGPLGEDGSGLLTELFREAAAAGRLDDAVTLTEAAARMRRTFTDAEDPAVRRTPVWFGRGPARPTVVCLPSFSAIAGVHVYARFA<br>DAFGDGWRVAALAHPGFVPGEPLPDSVDVLAELHARTVLDTVGADPFLLVGRSAGGWVAHEVAAVLERMGRAPDGVALLDTPARAD<br>DPRGHAVMVGGMLERDSRLVTIDDYRLTAMGGYSRLFREWKPEPIAAATLLVHAATPYGADEARIASWDLPHQAVKVTGDHFTMLE<br>RHSATTAEAVEQWSRSLLKLAAALEHHHHHHHH*
YZ009	MTGTEEKLVDYLKKVTAELQETRRQLRGALAASREPIAIVGMACRYPGGVRTPEALWRLVLDEQDAISGFPTNRGWDIDGIYHPDPDR<br>PGTCYAREGGFLHDAALFDAEFFGVSPREAQAMDPQQRLLLETAWEAFERAGIDPTSLRGSDTGVFAGVVHHDYATARVPETLEPYLV<br>TGLSGGVASGRIAYTFGFEGPAVTVDTACSSSLVALHQAAHALRSGECELALAGGVTIMATPRAFLSFSRQRGLSPDGRCRAFGAGADG<br>TGWAEGAGMLLVERLSDARRKGHPVLAVLRGSAVNQDGASNGLSAPNGPSQQRVIRKALAHAGLAARDVDVVEGHGTGTKLGDPIEA<br>QALLATYGQERDAGLPLHLGSMKSNVGHSQAAAGVGGVIKMIEAMRHGILPRTLHADEPTPHVDWSAGDIELLTRRRAWPETGRPRR<br>AAVSSFGISGTNAHVILEAPPEEPARDAAETRPEARSREAAEGRREAAGQGRTETGPGPSATPPEAPGRARPPVPWPLSGRDAGALRD<br>QIGRLRAHLDAAPADPEDVAHSLARRAVFRHRAVLLAAPQAPAGGSPRAVTGVARPGGTALLFSGMGTQWRGMGLSLMRLDSFRESI<br>LRSDEAVKPLGVKVSDLLLSTDERTFDDIVHAFVSLTAIQIALIDLLTSVGLKPDGIIGHSLGEVACGYADGCLSQREAVLAAYWRGQCIK<br>DAHLPPGSMAAVGLSWEECKQRCPAGVVPACHNSEDTVTISGPQAAVNEFVEQLKQEGVFAKEVRTGGLAFHSYFMEGIAPTLLQALK<br>KVIREPRPRSARWLSTSIPEAQWQSSLARTSSAEYNVNNLVSPVLFQEALWHIPEHAVVLEIAPHALLQAVLKRGVKSSCTIIPLMKRD<br>HKDNLEFFLTNLGKVHLDGGTVDWSGLCAGGRLVDLPTYPFQRQHYWIEDQPLPPTAPRPGTAPSGTGTAAEGAAAAEVPLSERLAR<br>LTGAERLAAVRELVLAEASETLGHTGTLITADRTRQELGFDSLTAIELRNRISRTLGVRLPPTLVFDHEDLGEIASFVDARLDDAATGRS<br>TGHGPLGEDGSGLLTELFREAAAAGRLDDAVTLTEAAARMRRTFTDAEDPAVRRTPVWFGRGPARPTVVCLPSFSAIAGVHVYARFA<br>DAFGDGWRVAALAHPGFVPGEPLPDSVDVLAELHARTVLDTVGADPFLLVGRSAGGWVAHEVAAVLERMGRAPDGVALLDTPARAD<br>DPRGHAVMVGGMLERDSRLVTIDDYRLTAMGGYSRLFREWKPEPIAAATLLVHAATPYGADEARIASWDLPHQAVKVTGDHFTMLE<br>RHSATTAEAVEQWSRSLLKLAAALEHHHHHHHH*
YZ014	MTGTEEKLVDYLKKVTAELQETRRQLRGALAASREPIAIVGMACRYPGGVRTPEALWRLVLDEQDAISGFPTNRGWDIDGIYHPDPDR<br>PGTCYAREGGFLHDAALFDAEFFGVSPREAQAMDPQQRLLLETAWEAFERAGIDPTSLRGSDTGVFAGVVHHDYATARVPETLEPYLV<br>TGLSGGVASGRIAYTFGFEGPAVTVDTACSSSLVALHQAAHALRSGECELALAGGVTIMATPRAFLSFSRQRGLSPDGRCRAFGAGADG<br>TGWAEGAGMLLVERLSDARRKGHPVLAVLRGSAVNQDGASNGLSAPNGPSQQRVIRKALAHAGLAARDVDVVEGHGTGTKLGDPIEA<br>QALLATYGQERDAGLPLHLGSMKSNVGHSQAAAGVGGVIKMIEAMRHGILPRTLHADEPTPHVDWSAGDIELLTRRRAWPETGRPRR<br>AAVSSFGISGTNAHVILEAPPEEPARDAAETRPEARSREAAEGRREAAGQGRTETGPGPSATPPTAHAALPHLLHASGRTLEAVQDLLE<br>QGRQHSQDLAFVSMLNDIAATPTAAMPFRGYTVLGVEGRVQEVQQVSTNKRPLWFICSGMGTQWRGMGLSLMRLDSFRESILRSDEA<br>VKPLGVKVSDLLLSTDERTFDDIVHAFVSLTAIQIALIDLLTSVGLKPDGIIGHSLGEVACGYADGCLSQREAVLAAYWRGQCIKDAHLPP<br>GSMAAVGLSWEECKQRCPAGVVPACHNSEDTVTISGPQAAVNEFVEQLKQEGVFAKEVRTGGLAFHSYFMEGIAPTLLQALKKVIREP<br>RPRSARWLSTSIPEAQWQSSLARTSSAEYNVNNLVSPVLFQEALWHIPEHAVVLEIAPHALLQAVLKRGVKSSCTIIPLMKRDHKDNLE<br>FFLTNLGKVHLTGINVNPNALFPPVEFPAPGGRLVDLPTYPFQRQHYWIEDQPLPPTAPRPGTAPSGTGTAAEGAAAAEVPLSERLARL<br>TGAERLAAVRELVLAEASETLGHTGTLITADRTRQELGFDSLTAIELRNRISRTLGVRLPPTLVFDHEDLGEIASFVDARLDDAATGRST<br>GHGPLGEDGSGLLTELFREAAAAGRLDDAVTLTEAAARMRRTFTDAEDPAVRRTPVWFGRGPARPTVVCLPSFSAIAGVHVYARFAD<br>AFGDGWRVAALAHPGFVPGEPLPDSVDVLAELHARTVLDTVGADPFLLVGRSAGGWVAHEVAAVLERMGRAPDGVALLDTPARADD<br>PRGHAVMVGGMLERDSRLVTIDDYRLTAMGGYSRLFREWKPEPIAAATLLVHAATPYGADEARIASWDLPHQAVKVTGDHFTMLER<br>HSATTAEAVEQWSRSLLKLAAALEHHHHHHHH*

Construct	Amino acid sequence
YZ015	MTGTEEKLVDYLKKVTAELQETRRQLRGALAASREPIAIVGMACRYPGGVRTPEALWRLVLDEQDAISGFPTNRGWDIDGIYHPDPDR<br>PGTCYAREGGFLHDAALFDAEFFGVSPREAQAMDPQQRLLLETAWEAFERAGIDPTSLRGSDTGVFAGVVHHDYATARVPETLEPYLV<br>TGLSGGVASGRIAYTFGFEGPAVTVDTACSSSLVALHQAAHALRSGECELALAGGVTIMATPRAFLSFSRQRGLSPDGRCRAFGAGADG<br>TGWAEGAGMLLVERLSDARRKGHPVLAVLRGSAVNQDGASNGLSAPNGPSQQRVIRKALAHAGLAARDVDVVEGHGTGTKLGDPIEA<br>QALLATYGQERDAGLPLHLGSMKSNVGHSQAAAGVGGVIKMIEAMRHGILPRTLHADEPTPHVDWSAGDIELLTRRRAWPETGRPRR<br>AAVSSFGISGTNAHVILEAPPEEPARDAAETRPEARSREAAEGRREAAGQGRTETGPGPSATPPTAHAALPHLLHASGRTLEAVQDLLE<br>QGRQHSQDLAFVSMLNDIAATPTAAMPFRGYTVLGVEGRVQEVQQVSTNKRPLWFICSGMGTQWRGMGLSLMRLDSFRESILRSDEA<br>VKPLGVKVSDLLLSTDERTFDDIVHAFVSLTAIQIALIDLLTSVGLKPDGIIGHSLGEVACGYADGCLSQREAVLAAYWRGQCIKDAHLPP<br>GSMAAVGLSWEECKQRCPAGVVPACHNSEDTVTISGPQAAVNEFVEQLKQEGVFAKEVRTGGLAFHSYFMEGIAPTLLQALKKVIREP<br>RPRSARWLSTSIPEAQWQSSLARTSSAEYNVNNLVSPVLFQEALWHIPEHAVVLEIAPHALLQAVLKRGVKSSCTIIPLMKRDHKDNLE<br>FFLTNLGKVHLTGINVNPNALFPPVEFPAPRGTLVDLPTYPFQRQHYWIEDQPLPPTAPRPGTAPSGTGTAAEGAAAAEVPLSERLARL<br>TGAERLAAVRELVLAEASETLGHTGTLITADRTRQELGFDSLTAIELRNRISRTLGVRLPPTLVFDHEDLGEIASFVDARLDDAATGRST<br>GHGPLGEDGSGLLTELFREAAAAGRLDDAVTLTEAAARMRRTFTDAEDPAVRRTPVWFGRGPARPTVVCLPSFSAIAGVHVYARFAD<br>AFGDGWRVAALAHPGFVPGEPLPDSVDVLAELHARTVLDTVGADPFLLVGRSAGGWVAHEVAAVLERMGRAPDGVALLDTPARADD<br>PRGHAVMVGGMLERDSRLVTIDDYRLTAMGGYSRLFREWKPEPIAAATLLVHAATPYGADEARIASWDLPHQAVKVTGDHFTMLER<br>HSATTAEAVEQWSRSLLKLAAALEHHHHHHHH*
YZ016	MTGTEEKLVDYLKKVTAELQETRRQLRGALAASREPIAIVGMACRYPGGVRTPEALWRLVLDEQDAISGFPTNRGWDIDGIYHPDPDR<br>PGTCYAREGGFLHDAALFDAEFFGVSPREAQAMDPQQRLLLETAWEAFERAGIDPTSLRGSDTGVFAGVVHHDYATARVPETLEPYLV<br>TGLSGGVASGRIAYTFGFEGPAVTVDTACSSSLVALHQAAHALRSGECELALAGGVTIMATPRAFLSFSRQRGLSPDGRCRAFGAGADG<br>TGWAEGAGMLLVERLSDARRKGHPVLAVLRGSAVNQDGASNGLSAPNGPSQQRVIRKALAHAGLAARDVDVVEGHGTGTKLGDPIEA<br>QALLATYGQERDAGLPLHLGSMKSNVGHSQAAAGVGGVIKMIEAMRHGILPRTLHADEPTPHVDWSAGDIELLTRRRAWPETGRPRR<br>AAVSSFGISGTNAHVILEAPPEEPARDAAETRPEARSREAAEGRREAAGQGRTETGPGPSATPPTAHAALPHLLHASGRTLEAVQDLLE<br>QGRQHSQDLAFVSMLNDIAATPTAAMPFRGYTVLGVEGRVQEVQQVSTNKRPLWFICSGMGTQWRGMGLSLMRLDSFRESILRSDEA<br>VKPLGVKVSDLLLSTDERTFDDIVHAFVSLTAIQIALIDLLTSVGLKPDGIIGHSLGEVACGYADGCLSQREAVLAAYWRGQCIKDAHLPP<br>GSMAAVGLSWEECKQRCPAGVVPACHNSEDTVTISGPQAAVNEFVEQLKQEGVFAKEVRTGGLAFHSYFMEGIAPTLLQALKKVIREP<br>RPRSARWLSTSIPEAQWQSSLARTSSAEYNVNNLVSPVLFQEALWHIPEHAVVLEIAPHALLQAVLKRGVKSSCTIIPLMKRDHKDNLE<br>FFLTNLGKVHLTGINVNPNALFPPVEFPAPRGTPLILPTYPFQRQHYWIEDQPLPPTAPRPGTAPSGTGTAAEGAAAAEVPLSERLARL<br>TGAERLAAVRELVLAEASETLGHTGTLITADRTRQELGFDSLTAIELRNRISRTLGVRLPPTLVFDHEDLGEIASFVDARLDDAATGRST<br>GHGPLGEDGSGLLTELFREAAAAGRLDDAVTLTEAAARMRRTFTDAEDPAVRRTPVWFGRGPARPTVVCLPSFSAIAGVHVYARFAD<br>AFGDGWRVAALAHPGFVPGEPLPDSVDVLAELHARTVLDTVGADPFLLVGRSAGGWVAHEVAAVLERMGRAPDGVALLDTPARADD<br>PRGHAVMVGGMLERDSRLVTIDDYRLTAMGGYSRLFREWKPEPIAAATLLVHAATPYGADEARIASWDLPHQAVKVTGDHFTMLER<br>HSATTAEAVEQWSRSLLKLAAALEHHHHHHHH*
YZ017	MTGTEEKLVDYLKKVTAELQETRRQLRGALAASREPIAIVGMACRYPGGVRTPEALWRLVLDEQDAISGFPTNRGWDIDGIYHPDPDR<br>PGTCYAREGGFLHDAALFDAEFFGVSPREAQAMDPQQRLLLETAWEAFERAGIDPTSLRGSDTGVFAGVVHHDYATARVPETLEPYLV<br>TGLSGGVASGRIAYTFGFEGPAVTVDTACSSSLVALHQAAHALRSGECELALAGGVTIMATPRAFLSFSRQRGLSPDGRCRAFGAGADG<br>TGWAEGAGMLLVERLSDARRKGHPVLAVLRGSAVNQDGASNGLSAPNGPSQQRVIRKALAHAGLAARDVDVVEGHGTGTKLGDPIEA<br>QALLATYGQERDAGLPLHLGSMKSNVGHSQAAAGVGGVIKMIEAMRHGILPRTLHADEPTPHVDWSAGDIELLTRRRAWPETGRPRR<br>AAVSSFGISGTNAHVILEAPPEEPARDAAETRPEARSREAAEGRREAAGQGRTETGPGPSATPPEAPGRAALPHLLHASGRTLEAVQDL<br>LEQGRQHSQDLAFVSMLNDIAATPTAAMPFRGYTVLGVEGRVQEVQQVSTNKRPLWFICSGMGTQWRGMGLSLMRLDSFRESILRSD<br>EAVKPLGVKVSDLLLSTDERTFDDIVHAFVSLTAIQIALIDLLTSVGLKPDGIIGHSLGEVACGYADGCLSQREAVLAAYWRGQCIKDAHL<br>PPGSMAAVGLSWEECKQRCPAGVVPACHNSEDTVTISGPQAAVNEFVEQLKQEGVFAKEVRTGGLAFHSYFMEGIAPTLLQALKKVIR<br>EPRPRSARWLSTSIPEAQWQSSLARTSSAEYNVNNLVSPVLFQEALWHIPEHAVVLEIAPHALLQAVLKRGVKSSCTIIPLMKRDHKDN<br>LEFFLTNLGKVHLTGINVNPNALFPPVEFPAPGGRLVDLPTYPFQRQHYWIEDQPLPPTAPRPGTAPSGTGTAAEGAAAAEVPLSERLA<br>RLTGAERLAAVRELVLAEASETLGHTGTLITADRTRQELGFDSLTAIELRNRISRTLGVRLPPTLVFDHEDLGEIASFVDARLDDAATGR<br>STGHGPLGEDGSGLLTELFREAAAAGRLDDAVTLTEAAARMRRTFTDAEDPAVRRTPVWFGRGPARPTVVCLPSFSAIAGVHVYARFA<br>DAFGDGWRVAALAHPGFVPGEPLPDSVDVLAELHARTVLDTVGADPFLLVGRSAGGWVAHEVAAVLERMGRAPDGVALLDTPARAD<br>DPRGHAVMVGGMLERDSRLVTIDDYRLTAMGGYSRLFREWKPEPIAAATLLVHAATPYGADEARIASWDLPHQAVKVTGDHFTMLE<br>RHSATTAEAVEQWSRSLLKLAAALEHHHHHHHH*

Construct	Amino acid sequence
YZ018	MTGTEEKLVDYLKKVTAELQETRRQLRGALAASREPIAIVGMACRYPGGVRTPEALWRLVLDEQDAISGFPTNRGWDIDGIYHPDPDR<br>PGTCYAREGGFLHDAALFDAEFFGVSPREAQAMDPQQRLLLETAWEAFERAGIDPTSLRGSDTGVFAGVVHHDYATARVPETLEPYLV<br>TGLSGGVASGRIAYTFGFEGPAVTVDTACSSSLVALHQAAHALRSGECELALAGGVTIMATPRAFLSFSRQRGLSPDGRCRAFGAGADG<br>TGWAEGAGMLLVERLSDARRKGHPVLAVLRGSAVNQDGASNGLSAPNGPSQQRVIRKALAHAGLAARDVDVVEGHGTGTKLGDPIEA<br>QALLATYGQERDAGLPLHLGSMKSNVGHSQAAAGVGGVIKMIEAMRHGILPRTLHADEPTPHVDWSAGDIELLTRRRAWPETGRPRR<br>AAVSSFGISGTNAHVILEAPPEEPARDAAETRPEARSREAAEGRREAAGQGRTETGPGPSATPPEAPGRAALPHLLHASGRTLEAVQDL<br>LEQGRQHSQDLAFVSMLNDIAATPTAAMPFRGYTVLGVEGRVQEVQQVSTNKRPLWFICSGMGTQWRGMGLSLMRLDSFRESILRSD<br>EAVKPLGVKVSDLLLSTDERTFDDIVHAFVSLTAIQIALIDLLTSVGLKPDGIIGHSLGEVACGYADGCLSQREAVLAAYWRGQCIKDAHL<br>PPGSMAAVGLSWEECKQRCPAGVVPACHNSEDTVTISGPQAAVNEFVEQLKQEGVFAKEVRTGGLAFHSYFMEGIAPTLLQALKKVIR<br>EPRPRSARWLSTSIPEAQWQSSLARTSSAEYNVNNLVSPVLFQEALWHIPEHAVVLEIAPHALLQAVLKRGVKSSCTIIPLMKRDHKDN<br>LEFFLTNLGKVHLTGINVNPNALFPPVEFPAPRGTLVDLPTYPFQRQHYWIEDQPLPPTAPRPGTAPSGTGTAAEGAAAAEVPLSERLA<br>RLTGAERLAAVRELVLAEASETLGHTGTLITADRTRQELGFDSLTAIELRNRISRTLGVRLPPTLVFDHEDLGEIASFVDARLDDAATGR<br>STGHGPLGEDGSGLLTELFREAAAAGRLDDAVTLTEAAARMRRTFTDAEDPAVRRTPVWFGRGPARPTVVCLPSFSAIAGVHVYARFA<br>DAFGDGWRVAALAHPGFVPGEPLPDSVDVLAELHARTVLDTVGADPFLLVGRSAGGWVAHEVAAVLERMGRAPDGVALLDTPARAD<br>DPRGHAVMVGGMLERDSRLVTIDDYRLTAMGGYSRLFREWKPEPIAAATLLVHAATPYGADEARIASWDLPHQAVKVTGDHFTMLE<br>RHSATTAEAVEQWSRSLLKLAAALEHHHHHHHH*
YZ019	MTGTEEKLVDYLKKVTAELQETRRQLRGALAASREPIAIVGMACRYPGGVRTPEALWRLVLDEQDAISGFPTNRGWDIDGIYHPDPDR<br>PGTCYAREGGFLHDAALFDAEFFGVSPREAQAMDPQQRLLLETAWEAFERAGIDPTSLRGSDTGVFAGVVHHDYATARVPETLEPYLV<br>TGLSGGVASGRIAYTFGFEGPAVTVDTACSSSLVALHQAAHALRSGECELALAGGVTIMATPRAFLSFSRQRGLSPDGRCRAFGAGADG<br>TGWAEGAGMLLVERLSDARRKGHPVLAVLRGSAVNQDGASNGLSAPNGPSQQRVIRKALAHAGLAARDVDVVEGHGTGTKLGDPIEA<br>QALLATYGQERDAGLPLHLGSMKSNVGHSQAAAGVGGVIKMIEAMRHGILPRTLHADEPTPHVDWSAGDIELLTRRRAWPETGRPRR<br>AAVSSFGISGTNAHVILEAPPEEPARDAAETRPEARSREAAEGRREAAGQGRTETGPGPSATPPEAPGRAALPHLLHASGRTLEAVQDL<br>LEQGRQHSQDLAFVSMLNDIAATPTAAMPFRGYTVLGVEGRVQEVQQVSTNKRPLWFICSGMGTQWRGMGLSLMRLDSFRESILRSD<br>EAVKPLGVKVSDLLLSTDERTFDDIVHAFVSLTAIQIALIDLLTSVGLKPDGIIGHSLGEVACGYADGCLSQREAVLAAYWRGQCIKDAHL<br>PPGSMAAVGLSWEECKQRCPAGVVPACHNSEDTVTISGPQAAVNEFVEQLKQEGVFAKEVRTGGLAFHSYFMEGIAPTLLQALKKVIR<br>EPRPRSARWLSTSIPEAQWQSSLARTSSAEYNVNNLVSPVLFQEALWHIPEHAVVLEIAPHALLQAVLKRGVKSSCTIIPLMKRDHKDN<br>LEFFLTNLGKVHLTGINVNPNALFPPVEFPAPRGTPLILPTYPFQRQHYWIEDQPLPPTAPRPGTAPSGTGTAAEGAAAAEVPLSERLA<br>RLTGAERLAAVRELVLAEASETLGHTGTLITADRTRQELGFDSLTAIELRNRISRTLGVRLPPTLVFDHEDLGEIASFVDARLDDAATGR<br>STGHGPLGEDGSGLLTELFREAAAAGRLDDAVTLTEAAARMRRTFTDAEDPAVRRTPVWFGRGPARPTVVCLPSFSAIAGVHVYARFA<br>DAFGDGWRVAALAHPGFVPGEPLPDSVDVLAELHARTVLDTVGADPFLLVGRSAGGWVAHEVAAVLERMGRAPDGVALLDTPARAD<br>DPRGHAVMVGGMLERDSRLVTIDDYRLTAMGGYSRLFREWKPEPIAAATLLVHAATPYGADEARIASWDLPHQAVKVTGDHFTMLE<br>RHSATTAEAVEQWSRSLLKLAAALEHHHHHHHH*
YZ020	MTGTEEKLVDYLKKVTAELQETRRQLRGALAASREPIAIVGMACRYPGGVRTPEALWRLVLDEQDAISGFPTNRGWDIDGIYHPDPDR<br>PGTCYAREGGFLHDAALFDAEFFGVSPREAQAMDPQQRLLLETAWEAFERAGIDPTSLRGSDTGVFAGVVHHDYATARVPETLEPYLV<br>TGLSGGVASGRIAYTFGFEGPAVTVDTACSSSLVALHQAAHALRSGECELALAGGVTIMATPRAFLSFSRQRGLSPDGRCRAFGAGADG<br>TGWAEGAGMLLVERLSDARRKGHPVLAVLRGSAVNQDGASNGLSAPNGPSQQRVIRKALAHAGLAARDVDVVEGHGTGTKLGDPIEA<br>QALLATYGQERDAGLPLHLGSMKSNVGHSQAAAGVGGVIKMIEAMRHGILPRTLHADEPTPHVDWSAGDIELLTRRRAWPETGRPRR<br>AAVSSFGISGTNAHVILEAPPEEPARDAAETRPEARSREAAEGRREAAGQGRTETGPGPSATPPEAPGRARPPHLLHASGRTLEAVQDL<br>LEQGRQHSQDLAFVSMLNDIAATPTAAMPFRGYTVLGVEGRVQEVQQVSTNKRPLWFICSGMGTQWRGMGLSLMRLDSFRESILRSD<br>EAVKPLGVKVSDLLLSTDERTFDDIVHAFVSLTAIQIALIDLLTSVGLKPDGIIGHSLGEVACGYADGCLSQREAVLAAYWRGQCIKDAHL<br>PPGSMAAVGLSWEECKQRCPAGVVPACHNSEDTVTISGPQAAVNEFVEQLKQEGVFAKEVRTGGLAFHSYFMEGIAPTLLQALKKVIR<br>EPRPRSARWLSTSIPEAQWQSSLARTSSAEYNVNNLVSPVLFQEALWHIPEHAVVLEIAPHALLQAVLKRGVKSSCTIIPLMKRDHKDN<br>LEFFLTNLGKVHLTGINVNPNALFPPVEFPAPGGRLVDLPTYPFQRQHYWIEDQPLPPTAPRPGTAPSGTGTAAEGAAAAEVPLSERLA<br>RLTGAERLAAVRELVLAEASETLGHTGTLITADRTRQELGFDSLTAIELRNRISRTLGVRLPPTLVFDHEDLGEIASFVDARLDDAATGR<br>STGHGPLGEDGSGLLTELFREAAAAGRLDDAVTLTEAAARMRRTFTDAEDPAVRRTPVWFGRGPARPTVVCLPSFSAIAGVHVYARFA<br>DAFGDGWRVAALAHPGFVPGEPLPDSVDVLAELHARTVLDTVGADPFLLVGRSAGGWVAHEVAAVLERMGRAPDGVALLDTPARAD<br>DPRGHAVMVGGMLERDSRLVTIDDYRLTAMGGYSRLFREWKPEPIAAATLLVHAATPYGADEARIASWDLPHQAVKVTGDHFTMLE<br>RHSATTAEAVEQWSRSLLKLAAALEHHHHHHHH*

Construct	Amino acid sequence
YZ021	MTGTEEKLVDYLKKVTAELQETRRQLRGALAASREPIAIVGMACRYPGGVRTPEALWRLVLDEQDAISGFPTNRGWDIDGIYHPDPDR<br>PGTCYAREGGFLHDAALFDAEFFGVSPREAQAMDPQQRLLLETAWEAFERAGIDPTSLRGSDTGVFAGVVHHDYATARVPETLEPYLV<br>TGLSGGVASGRIAYTFGFEGPAVTVDTACSSSLVALHQAAHALRSGECELALAGGVTIMATPRAFLSFSRQRGLSPDGRCRAFGAGADG<br>TGWAEGAGMLLVERLSDARRKGHPVLAVLRGSAVNQDGASNGLSAPNGPSQQRVIRKALAHAGLAARDVDVVEGHGTGTKLGDPIEA<br>QALLATYGQERDAGLPLHLGSMKSNVGHSQAAAGVGGVIKMIEAMRHGILPRTLHADEPTPHVDWSAGDIELLTRRRAWPETGRPRR<br>AAVSSFGISGTNAHVILEAPPEEPARDAAETRPEARSREAAEGRREAAGQGRTETGPGPSATPPEAPGRARPPHLLHASGRTLEAVQDL<br>LEQGRQHSQDLAFVSMLNDIAATPTAAMPFRGYTVLGVEGRVQEVQQVSTNKRPLWFICSGMGTQWRGMGLSLMRLDSFRESILRSD<br>EAVKPLGVKVSDLLLSTDERTFDDIVHAFVSLTAIQIALIDLLTSVGLKPDGIIGHSLGEVACGYADGCLSQREAVLAAYWRGQCIKDAHL<br>PPGSMAAVGLSWEECKQRCPAGVVPACHNSEDTVTISGPQAAVNEFVEQLKQEGVFAKEVRTGGLAFHSYFMEGIAPTLLQALKKVIR<br>EPRPRSARWLSTSIPEAQWQSSLARTSSAEYNVNNLVSPVLFQEALWHIPEHAVVLEIAPHALLQAVLKRGVKSSCTIIPLMKRDHKDN<br>LEFFLTNLGKVHLTGINVNPNALFPPVEFPAPRGTLVDLPTYPFQRQHYWIEDQPLPPTAPRPGTAPSGTGTAAEGAAAAEVPLSERLA<br>RLTGAERLAAVRELVLAEASETLGHTGTLITADRTRQELGFDSLTAIELRNRISRTLGVRLPPTLVFDHEDLGEIASFVDARLDDAATGR<br>STGHGPLGEDGSGLLTELFREAAAAGRLDDAVTLTEAAARMRRTFTDAEDPAVRRTPVWFGRGPARPTVVCLPSFSAIAGVHVYARFA<br>DAFGDGWRVAALAHPGFVPGEPLPDSVDVLAELHARTVLDTVGADPFLLVGRSAGGWVAHEVAAVLERMGRAPDGVALLDTPARAD<br>DPRGHAVMVGGMLERDSRLVTIDDYRLTAMGGYSRLFREWKPEPIAAATLLVHAATPYGADEARIASWDLPHQAVKVTGDHFTMLE<br>RHSATTAEAVEQWSRSLLKLAAALEHHHHHHHH*
YZ022	MTGTEEKLVDYLKKVTAELQETRRQLRGALAASREPIAIVGMACRYPGGVRTPEALWRLVLDEQDAISGFPTNRGWDIDGIYHPDPDR<br>PGTCYAREGGFLHDAALFDAEFFGVSPREAQAMDPQQRLLLETAWEAFERAGIDPTSLRGSDTGVFAGVVHHDYATARVPETLEPYLV<br>TGLSGGVASGRIAYTFGFEGPAVTVDTACSSSLVALHQAAHALRSGECELALAGGVTIMATPRAFLSFSRQRGLSPDGRCRAFGAGADG<br>TGWAEGAGMLLVERLSDARRKGHPVLAVLRGSAVNQDGASNGLSAPNGPSQQRVIRKALAHAGLAARDVDVVEGHGTGTKLGDPIEA<br>QALLATYGQERDAGLPLHLGSMKSNVGHSQAAAGVGGVIKMIEAMRHGILPRTLHADEPTPHVDWSAGDIELLTRRRAWPETGRPRR<br>AAVSSFGISGTNAHVILEAPPEEPARDAAETRPEARSREAAEGRREAAGQGRTETGPGPSATPPEAPGRARPPHLLHASGRTLEAVQDL<br>LEQGRQHSQDLAFVSMLNDIAATPTAAMPFRGYTVLGVEGRVQEVQQVSTNKRPLWFICSGMGTQWRGMGLSLMRLDSFRESILRSD<br>EAVKPLGVKVSDLLLSTDERTFDDIVHAFVSLTAIQIALIDLLTSVGLKPDGIIGHSLGEVACGYADGCLSQREAVLAAYWRGQCIKDAHL<br>PPGSMAAVGLSWEECKQRCPAGVVPACHNSEDTVTISGPQAAVNEFVEQLKQEGVFAKEVRTGGLAFHSYFMEGIAPTLLQALKKVIR<br>EPRPRSARWLSTSIPEAQWQSSLARTSSAEYNVNNLVSPVLFQEALWHIPEHAVVLEIAPHALLQAVLKRGVKSSCTIIPLMKRDHKDN<br>LEFFLTNLGKVHLTGINVNPNALFPPVEFPAPRGTPLILPTYPFQRQHYWIEDQPLPPTAPRPGTAPSGTGTAAEGAAAAEVPLSERLA<br>RLTGAERLAAVRELVLAEASETLGHTGTLITADRTRQELGFDSLTAIELRNRISRTLGVRLPPTLVFDHEDLGEIASFVDARLDDAATGR<br>STGHGPLGEDGSGLLTELFREAAAAGRLDDAVTLTEAAARMRRTFTDAEDPAVRRTPVWFGRGPARPTVVCLPSFSAIAGVHVYARFA<br>DAFGDGWRVAALAHPGFVPGEPLPDSVDVLAELHARTVLDTVGADPFLLVGRSAGGWVAHEVAAVLERMGRAPDGVALLDTPARAD<br>DPRGHAVMVGGMLERDSRLVTIDDYRLTAMGGYSRLFREWKPEPIAAATLLVHAATPYGADEARIASWDLPHQAVKVTGDHFTMLE<br>RHSATTAEAVEQWSRSLLKLAAALEHHHHHHHH*
YZ023	MTGTEEKLVDYLKKVTAELQETRRQLRGALAASREPIAIVGMACRYPGGVRTPEALWRLVLDEQDAISGFPTNRGWDIDGIYHPDPDR<br>PGTCYAREGGFLHDAALFDAEFFGVSPREAQAMDPQQRLLLETAWEAFERAGIDPTSLRGSDTGVFAGVVHHDYATARVPETLEPYLV<br>TGLSGGVASGRIAYTFGFEGPAVTVDTACSSSLVALHQAAHALRSGECELALAGGVTIMATPRAFLSFSRQRGLSPDGRCRAFGAGADG<br>TGWAEGAGMLLVERLSDARRKGHPVLAVLRGSAVNQDGASNGLSAPNGPSQQRVIRKALAHAGLAARDVDVVEGHGTGTKLGDPIEA<br>QALLATYGQERDAGLPLHLGSMKSNVGHSQAAAGVGGVIKMIEAMRHGILPRTLHADEPTPHVDWSAGDIELLTRRRAWPETGRPRR<br>AAVSSFGISGTNAHVILEAPPEEPARDAAETRPEARSREAAEGRREAAGQGRTETGPGPSATPPEAPGRARPPVLLHASGRTLEAVQDL<br>LEQGRQHSQDLAFVSMLNDIAATPTAAMPFRGYTVLGVEGRVQEVQQVSTNKRPLWFICSGMGTQWRGMGLSLMRLDSFRESILRSD<br>EAVKPLGVKVSDLLLSTDERTFDDIVHAFVSLTAIQIALIDLLTSVGLKPDGIIGHSLGEVACGYADGCLSQREAVLAAYWRGQCIKDAHL<br>PPGSMAAVGLSWEECKQRCPAGVVPACHNSEDTVTISGPQAAVNEFVEQLKQEGVFAKEVRTGGLAFHSYFMEGIAPTLLQALKKVIR<br>EPRPRSARWLSTSIPEAQWQSSLARTSSAEYNVNNLVSPVLFQEALWHIPEHAVVLEIAPHALLQAVLKRGVKSSCTIIPLMKRDHKDN<br>LEFFLTNLGKVHLTGINVNPNALFPPVEFPAPGGRLVDLPTYPFQRQHYWIEDQPLPPTAPRPGTAPSGTGTAAEGAAAAEVPLSERLA<br>RLTGAERLAAVRELVLAEASETLGHTGTLITADRTRQELGFDSLTAIELRNRISRTLGVRLPPTLVFDHEDLGEIASFVDARLDDAATGR<br>STGHGPLGEDGSGLLTELFREAAAAGRLDDAVTLTEAAARMRRTFTDAEDPAVRRTPVWFGRGPARPTVVCLPSFSAIAGVHVYARFA<br>DAFGDGWRVAALAHPGFVPGEPLPDSVDVLAELHARTVLDTVGADPFLLVGRSAGGWVAHEVAAVLERMGRAPDGVALLDTPARAD<br>DPRGHAVMVGGMLERDSRLVTIDDYRLTAMGGYSRLFREWKPEPIAAATLLVHAATPYGADEARIASWDLPHQAVKVTGDHFTMLE<br>RHSATTAEAVEQWSRSLLKLAAALEHHHHHHHH*

Construct	Amino acid sequence
YZ024	MTGTEEKLVDYLKKVTAELQETRRQLRGALAASREPIAIVGMACRYPGGVRTPEALWRLVLDEQDAISGFPTNRGWDIDGIYHPDPDR<br>PGTCYAREGGFLHDAALFDAEFFGVSPREAQAMDPQQRLLLETAWEAFERAGIDPTSLRGSDTGVFAGVVHHDYATARVPETLEPYLV<br>TGLSGGVASGRIAYTFGFEGPAVTVDTACSSSLVALHQAAHALRSGECELALAGGVTIMATPRAFLSFSRQRGLSPDGRCRAFGAGADG<br>TGWAEGAGMLLVERLSDARRKGHPVLAVLRGSAVNQDGASNGLSAPNGPSQQRVIRKALAHAGLAARDVDVVEGHGTGTKLGDPIEA<br>QALLATYGQERDAGLPLHLGSMKSNVGHSQAAAGVGGVIKMIEAMRHGILPRTLHADEPTPHVDWSAGDIELLTRRRAWPETGRPRR<br>AAVSSFGISGTNAHVILEAPPEEPARDAAETRPEARSREAAEGRREAAGQGRTETGPGPSATPPEAPGRARPPVLLHASGRTLEAVQDL<br>LEQGRQHSQDLAFVSMLNDIAATPTAAMPFRGYTVLGVEGRVQEVQQVSTNKRPLWFICSGMGTQWRGMGLSLMRLDSFRESILRSD<br>EAVKPLGVKVSDLLLSTDERTFDDIVHAFVSLTAIQIALIDLLTSVGLKPDGIIGHSLGEVACGYADGCLSQREAVLAAYWRGQCIKDAHL<br>PPGSMAAVGLSWEECKQRCPAGVVPACHNSEDTVTISGPQAAVNEFVEQLKQEGVFAKEVRTGGLAFHSYFMEGIAPTLLQALKKVIR<br>EPRPRSARWLSTSIPEAQWQSSLARTSSAEYNVNNLVSPVLFQEALWHIPEHAVVLEIAPHALLQAVLKRGVKSSCTIIPLMKRDHKDN<br>LEFFLTNLGKVHLTGINVNPNALFPPVEFPAPRGTLVDLPTYPFQRQHYWIEDQPLPPTAPRPGTAPSGTGTAAEGAAAAEVPLSERLA<br>RLTGAERLAAVRELVLAEASETLGHTGTLITADRTRQELGFDSLTAIELRNRISRTLGVRLPPTLVFDHEDLGEIASFVDARLDDAATGR<br>STGHGPLGEDGSGLLTELFREAAAAGRLDDAVTLTEAAARMRRTFTDAEDPAVRRTPVWFGRGPARPTVVCLPSFSAIAGVHVYARFA<br>DAFGDGWRVAALAHPGFVPGEPLPDSVDVLAELHARTVLDTVGADPFLLVGRSAGGWVAHEVAAVLERMGRAPDGVALLDTPARAD<br>DPRGHAVMVGGMLERDSRLVTIDDYRLTAMGGYSRLFREWKPEPIAAATLLVHAATPYGADEARIASWDLPHQAVKVTGDHFTMLE<br>RHSATTAEAVEQWSRSLLKLAAALEHHHHHHHH*
YZ025	MTGTEEKLVDYLKKVTAELQETRRQLRGALAASREPIAIVGMACRYPGGVRTPEALWRLVLDEQDAISGFPTNRGWDIDGIYHPDPDR<br>PGTCYAREGGFLHDAALFDAEFFGVSPREAQAMDPQQRLLLETAWEAFERAGIDPTSLRGSDTGVFAGVVHHDYATARVPETLEPYLV<br>TGLSGGVASGRIAYTFGFEGPAVTVDTACSSSLVALHQAAHALRSGECELALAGGVTIMATPRAFLSFSRQRGLSPDGRCRAFGAGADG<br>TGWAEGAGMLLVERLSDARRKGHPVLAVLRGSAVNQDGASNGLSAPNGPSQQRVIRKALAHAGLAARDVDVVEGHGTGTKLGDPIEA<br>QALLATYGQERDAGLPLHLGSMKSNVGHSQAAAGVGGVIKMIEAMRHGILPRTLHADEPTPHVDWSAGDIELLTRRRAWPETGRPRR<br>AAVSSFGISGTNAHVILEAPPEEPARDAAETRPEARSREAAEGRREAAGQGRTETGPGPSATPPEAPGRARPPVLLHASGRTLEAVQDL<br>LEQGRQHSQDLAFVSMLNDIAATPTAAMPFRGYTVLGVEGRVQEVQQVSTNKRPLWFICSGMGTQWRGMGLSLMRLDSFRESILRSD<br>EAVKPLGVKVSDLLLSTDERTFDDIVHAFVSLTAIQIALIDLLTSVGLKPDGIIGHSLGEVACGYADGCLSQREAVLAAYWRGQCIKDAHL<br>PPGSMAAVGLSWEECKQRCPAGVVPACHNSEDTVTISGPQAAVNEFVEQLKQEGVFAKEVRTGGLAFHSYFMEGIAPTLLQALKKVIR<br>EPRPRSARWLSTSIPEAQWQSSLARTSSAEYNVNNLVSPVLFQEALWHIPEHAVVLEIAPHALLQAVLKRGVKSSCTIIPLMKRDHKDN<br>LEFFLTNLGKVHLTGINVNPNALFPPVEFPAPRGTPLILPTYPFQRQHYWIEDQPLPPTAPRPGTAPSGTGTAAEGAAAAEVPLSERLA<br>RLTGAERLAAVRELVLAEASETLGHTGTLITADRTRQELGFDSLTAIELRNRISRTLGVRLPPTLVFDHEDLGEIASFVDARLDDAATGR<br>STGHGPLGEDGSGLLTELFREAAAAGRLDDAVTLTEAAARMRRTFTDAEDPAVRRTPVWFGRGPARPTVVCLPSFSAIAGVHVYARFA<br>DAFGDGWRVAALAHPGFVPGEPLPDSVDVLAELHARTVLDTVGADPFLLVGRSAGGWVAHEVAAVLERMGRAPDGVALLDTPARAD<br>DPRGHAVMVGGMLERDSRLVTIDDYRLTAMGGYSRLFREWKPEPIAAATLLVHAATPYGADEARIASWDLPHQAVKVTGDHFTMLE<br>RHSATTAEAVEQWSRSLLKLAAALEHHHHHHHH*
YZ026	MTGTEEKLVDYLKKVTAELQETRRQLRGALAASREPIAIVGMACRYPGGVRTPEALWRLVLDEQDAISGFPTNRGWDIDGIYHPDPDR<br>PGTCYAREGGFLHDAALFDAEFFGVSPREAQAMDPQQRLLLETAWEAFERAGIDPTSLRGSDTGVFAGVVHHDYATARVPETLEPYLV<br>TGLSGGVASGRIAYTFGFEGPAVTVDTACSSSLVALHQAAHALRSGECELALAGGVTIMATPRAFLSFSRQRGLSPDGRCRAFGAGADG<br>TGWAEGAGMLLVERLSDARRKGHPVLAVLRGSAVNQDGASNGLSAPNGPSQQRVIRKALAHAGLAARDVDVVEGHGTGTKLGDPIEA<br>QALLATYGQERDAGLPLHLGSMKSNVGHSQAAAGVGGVIKMIEAMRHGILPRTLHADEPTPHVDWSAGDIELLTRRRAWPETGRPRR<br>AAVSSFGISGTNAHVILEAPPEEPARDAAETRPEARSREAAEGRREAAGQGRTETGPGPSATPPTAHAALPHLLHASGRTLEAVQDLLE<br>QGRQHSQDLAFVSMLNDIAATPTAAMPFRGYTVLGVEGRVQEVQQVSTNKRPLWFICSGMGTQWRGMGLSLMRLDSFRESILRSDEA<br>VKPLGVKVSDLLLSTDERTFDDIVHAFVSLTAIQIALIDLLTSVGLKPDGIIGHSLGEVACGYADGCLSQREAVLAAYWRGQCIKDAHLPP<br>GSMAAVGLSWEECKQRCPAGVVPACHNSEDTVTISGPQAAVNEFVEQLKQEGVFAKEVRTGGLAFHSYFMEGIAPTLLQALKKVIREP<br>RPRSARWLSTSIPEAQWQSSLARTSSAEYNVNNLVSPVLFQEALWHIPEHAVVLEIAPHALLQAVLKRGVKSSCTIIPLMKRDHKDNLE<br>FFLTNLGQVHVDGGTVDWSGLCAGGRLVDLPTYPFQRQHYWIEDQPLPPTAPRPGTAPSGTGTAAEGAAAAEVPLSERLARLTGAER<br>LAAVRELVLAEASETLGHTGTLITADRTRQELGFDSLTAIELRNRISRTLGVRLPPTLVFDHEDLGEIASFVDARLDDAATGRSTGHGPL<br>GEDGSGLLTELFREAAAAGRLDDAVTLTEAAARMRRTFTDAEDPAVRRTPVWFGRGPARPTVVCLPSFSAIAGVHVYARFADAFGDG<br>WRVAALAHPGFVPGEPLPDSVDVLAELHARTVLDTVGADPFLLVGRSAGGWVAHEVAAVLERMGRAPDGVALLDTPARADDPRGHA<br>VMVGGMLERDSRLVTIDDYRLTAMGGYSRLFREWKPEPIAAATLLVHAATPYGADEARIASWDLPHQAVKVTGDHFTMLERHSATT<br>AEAVEQWSRSLLKLAAALEHHHHHHHH*

Construct	Amino acid sequence
YZ027	MTGTEEKLVDYLKKVTAELQETRRQLRGALAASREPIAIVGMACRYPGGVRTPEALWRLVLDEQDAISGFPTNRGWDIDGIYHPDPDR<br>PGTCYAREGGFLHDAALFDAEFFGVSPREAQAMDPQQRLLLETAWEAFERAGIDPTSLRGSDTGVFAGVVHHDYATARVPETLEPYLV<br>TGLSGGVASGRIAYTFGFEGPAVTVDTACSSSLVALHQAAHALRSGECELALAGGVTIMATPRAFLSFSRQRGLSPDGRCRAFGAGADG<br>TGWAEGAGMLLVERLSDARRKGHPVLAVLRGSAVNQDGASNGLSAPNGPSQQRVIRKALAHAGLAARDVDVVEGHGTGTKLGDPIEA<br>QALLATYGQERDAGLPLHLGSMKSNVGHSQAAAGVGGVIKMIEAMRHGILPRTLHADEPTPHVDWSAGDIELLTRRRAWPETGRPRR<br>AAVSSFGISGTNAHVILEAPPEEPARDAAETRPEARSREAAEGRREAAGQGRTETGPGPSATPPTAHAALPHLLHASGRTLEAVQDLLE<br>QGRQHSQDLAFVSMLNDIAATPTAAMPFRGYTVLGVEGRVQEVQQVSTNKRPLWFICSGMGTQWRGMGLSLMRLDSFRESILRSDEA<br>VKPLGVKVSDLLLSTDERTFDDIVHAFVSLTAIQIALIDLLTSVGLKPDGIIGHSLGEVACGYADGCLSQREAVLAAYWRGQCIKDAHLPP<br>GSMAAVGLSWEECKQRCPAGVVPACHNSEDTVTISGPQAAVNEFVEQLKQEGVFAKEVRTGGLAFHSYFMEGIAPTLLQALKKVIREP<br>RPRSARWLSTSIPEAQWQSSLARTSSAEYNVNNLVSPVLFQEALWHIPEHAVVLEIAPHALLQAVLKRGVKSSCTIIPLMKRDHKDNLE<br>FFLTNLGKVHLDGGTVDWSGLCAGGRLVDLPTYPFQRQHYWIEDQPLPPTAPRPGTAPSGTGTAAEGAAAAEVPLSERLARLTGAERL<br>AAVRELVLAEASETLGHTGTLITADRTRQELGFDSLTAIELRNRISRTLGVRLPPTLVFDHEDLGEIASFVDARLDDAATGRSTGHGPLG<br>EDGSGLLTELFREAAAAGRLDDAVTLTEAAARMRRTFTDAEDPAVRRTPVWFGRGPARPTVVCLPSFSAIAGVHVYARFADAFGDGW<br>RVAALAHPGFVPGEPLPDSVDVLAELHARTVLDTVGADPFLLVGRSAGGWVAHEVAAVLERMGRAPDGVALLDTPARADDPRGHAV<br>MVGGMLERDSRLVTIDDYRLTAMGGYSRLFREWKPEPIAAATLLVHAATPYGADEARIASWDLPHQAVKVTGDHFTMLERHSATTA<br>EAVEQWSRSLLKLAAALEHHHHHHHH*
YZ028	MTGTEEKLVDYLKKVTAELQETRRQLRGALAASREPIAIVGMACRYPGGVRTPEALWRLVLDEQDAISGFPTNRGWDIDGIYHPDPDR<br>PGTCYAREGGFLHDAALFDAEFFGVSPREAQAMDPQQRLLLETAWEAFERAGIDPTSLRGSDTGVFAGVVHHDYATARVPETLEPYLV<br>TGLSGGVASGRIAYTFGFEGPAVTVDTACSSSLVALHQAAHALRSGECELALAGGVTIMATPRAFLSFSRQRGLSPDGRCRAFGAGADG<br>TGWAEGAGMLLVERLSDARRKGHPVLAVLRGSAVNQDGASNGLSAPNGPSQQRVIRKALAHAGLAARDVDVVEGHGTGTKLGDPIEA<br>QALLATYGQERDAGLPLHLGSMKSNVGHSQAAAGVGGVIKMIEAMRHGILPRTLHADEPTPHVDWSAGDIELLTRRRAWPETGRPRR<br>AAVSSFGISGTNAHVILEAPPEEPARDAAETRPEARSREAAEGRREAAGQGRTETGPGPSATPPTAHAALPHLLHASGRTLEAVQDLLE<br>QGRQHSQDLAFVSMLNDIAATPTAAMPFRGYTVLGVEGRVQEVQQVSTNKRPLWFICSGMGTQWRGMGLSLMRLDSFRESILRSDEA<br>VKPLGVKVSDLLLSTDERTFDDIVHAFVSLTAIQIALIDLLTSVGLKPDGIIGHSLGEVACGYADGCLSQREAVLAAYWRGQCIKDAHLPP<br>GSMAAVGLSWEECKQRCPAGVVPACHNSEDTVTISGPQAAVNEFVEQLKQEGVFAKEVRTGGLAFHSYFMEGIAPTLLQALKKVIREP<br>RPRSARWLSTSIPEAQWQSSLARTSSAEYNVNNLVSPVLFQEALWHIPEHAVVLEIAPHALLQAVLKRGVKSSCTIIPLMKRDHKDNLE<br>FFLTNLGKVHLTGGTVDWSGLCAGGRLVDLPTYPFQRQHYWIEDQPLPPTAPRPGTAPSGTGTAAEGAAAAEVPLSERLARLTGAERL<br>AAVRELVLAEASETLGHTGTLITADRTRQELGFDSLTAIELRNRISRTLGVRLPPTLVFDHEDLGEIASFVDARLDDAATGRSTGHGPLG<br>EDGSGLLTELFREAAAAGRLDDAVTLTEAAARMRRTFTDAEDPAVRRTPVWFGRGPARPTVVCLPSFSAIAGVHVYARFADAFGDGW<br>RVAALAHPGFVPGEPLPDSVDVLAELHARTVLDTVGADPFLLVGRSAGGWVAHEVAAVLERMGRAPDGVALLDTPARADDPRGHAV<br>MVGGMLERDSRLVTIDDYRLTAMGGYSRLFREWKPEPIAAATLLVHAATPYGADEARIASWDLPHQAVKVTGDHFTMLERHSATTA<br>EAVEQWSRSLLKLAAALEHHHHHHHH*
YZ029	MTGTEEKLVDYLKKVTAELQETRRQLRGALAASREPIAIVGMACRYPGGVRTPEALWRLVLDEQDAISGFPTNRGWDIDGIYHPDPDR<br>PGTCYAREGGFLHDAALFDAEFFGVSPREAQAMDPQQRLLLETAWEAFERAGIDPTSLRGSDTGVFAGVVHHDYATARVPETLEPYLV<br>TGLSGGVASGRIAYTFGFEGPAVTVDTACSSSLVALHQAAHALRSGECELALAGGVTIMATPRAFLSFSRQRGLSPDGRCRAFGAGADG<br>TGWAEGAGMLLVERLSDARRKGHPVLAVLRGSAVNQDGASNGLSAPNGPSQQRVIRKALAHAGLAARDVDVVEGHGTGTKLGDPIEA<br>QALLATYGQERDAGLPLHLGSMKSNVGHSQAAAGVGGVIKMIEAMRHGILPRTLHADEPTPHVDWSAGDIELLTRRRAWPETGRPRR<br>AAVSSFGISGTNAHVILEAPPEEPARDAAETRPEARSREAAEGRREAAGQGRTETGPGPSATPPEAPGRAALPHLLHASGRTLEAVQDL<br>LEQGRQHSQDLAFVSMLNDIAATPTAAMPFRGYTVLGVEGRVQEVQQVSTNKRPLWFICSGMGTQWRGMGLSLMRLDSFRESILRSD<br>EAVKPLGVKVSDLLLSTDERTFDDIVHAFVSLTAIQIALIDLLTSVGLKPDGIIGHSLGEVACGYADGCLSQREAVLAAYWRGQCIKDAHL<br>PPGSMAAVGLSWEECKQRCPAGVVPACHNSEDTVTISGPQAAVNEFVEQLKQEGVFAKEVRTGGLAFHSYFMEGIAPTLLQALKKVIR<br>EPRPRSARWLSTSIPEAQWQSSLARTSSAEYNVNNLVSPVLFQEALWHIPEHAVVLEIAPHALLQAVLKRGVKSSCTIIPLMKRDHKDN<br>LEFFLTNLGQVHVDGGTVDWSGLCAGGRLVDLPTYPFQRQHYWIEDQPLPPTAPRPGTAPSGTGTAAEGAAAAEVPLSERLARLTGAE<br>RLAAVRELVLAEASETLGHTGTLITADRTRQELGFDSLTAIELRNRISRTLGVRLPPTLVFDHEDLGEIASFVDARLDDAATGRSTGHGP<br>LGEDGSGLLTELFREAAAAGRLDDAVTLTEAAARMRRTFTDAEDPAVRRTPVWFGRGPARPTVVCLPSFSAIAGVHVYARFADAFGD<br>GWRVAALAHPGFVPGEPLPDSVDVLAELHARTVLDTVGADPFLLVGRSAGGWVAHEVAAVLERMGRAPDGVALLDTPARADDPRGH<br>AVMVGGMLERDSRLVTIDDYRLTAMGGYSRLFREWKPEPIAAATLLVHAATPYGADEARIASWDLPHQAVKVTGDHFTMLERHSAT<br>TAEAVEQWSRSLLKLAAALEHHHHHHHH*

Construct	Amino acid sequence
YZ030	MTGTEEKLVDYLKKVTAELQETRRQLRGALAASREPIAIVGMACRYPGGVRTPEALWRLVLDEQDAISGFPTNRGWDIDGIYHPDPDR<br>PGTCYAREGGFLHDAALFDAEFFGVSPREAQAMDPQQRLLLETAWEAFERAGIDPTSLRGSDTGVFAGVVHHDYATARVPETLEPYLV<br>TGLSGGVASGRIAYTFGFEGPAVTVDTACSSSLVALHQAAHALRSGECELALAGGVTIMATPRAFLSFSRQRGLSPDGRCRAFGAGADG<br>TGWAEGAGMLLVERLSDARRKGHPVLAVLRGSAVNQDGASNGLSAPNGPSQQRVIRKALAHAGLAARDVDVVEGHGTGTKLGDPIEA<br>QALLATYGQERDAGLPLHLGSMKSNVGHSQAAAGVGGVIKMIEAMRHGILPRTLHADEPTPHVDWSAGDIELLTRRRAWPETGRPRR<br>AAVSSFGISGTNAHVILEAPPEEPARDAAETRPEARSREAAEGRREAAGQGRTETGPGPSATPPEAPGRAALPHLLHASGRTLEAVQDL<br>LEQGRQHSQDLAFVSMLNDIAATPTAAMPFRGYTVLGVEGRVQEVQQVSTNKRPLWFICSGMGTQWRGMGLSLMRLDSFRESILRSD<br>EAVKPLGVKVSDLLLSTDERTFDDIVHAFVSLTAIQIALIDLLTSVGLKPDGIIGHSLGEVACGYADGCLSQREAVLAAYWRGQCIKDAHL<br>PPGSMAAVGLSWEECKQRCPAGVVPACHNSEDTVTISGPQAAVNEFVEQLKQEGVFAKEVRTGGLAFHSYFMEGIAPTLLQALKKVIR<br>EPRPRSARWLSTSIPEAQWQSSLARTSSAEYNVNNLVSPVLFQEALWHIPEHAVVLEIAPHALLQAVLKRGVKSSCTIIPLMKRDHKDN<br>LEFFLTNLGKVHLDGGTVDWSGLCAGGRLVDLPTYPFQRQHYWIEDQPLPPTAPRPGTAPSGTGTAAEGAAAAEVPLSERLARLTGAE<br>RLAAVRELVLAEASETLGHTGTLITADRTRQELGFDSLTAIELRNRISRTLGVRLPPTLVFDHEDLGEIASFVDARLDDAATGRSTGHGP<br>LGEDGSGLLTELFREAAAAGRLDDAVTLTEAAARMRRTFTDAEDPAVRRTPVWFGRGPARPTVVCLPSFSAIAGVHVYARFADAFGD<br>GWRVAALAHPGFVPGEPLPDSVDVLAELHARTVLDTVGADPFLLVGRSAGGWVAHEVAAVLERMGRAPDGVALLDTPARADDPRGH<br>AVMVGGMLERDSRLVTIDDYRLTAMGGYSRLFREWKPEPIAAATLLVHAATPYGADEARIASWDLPHQAVKVTGDHFTMLERHSAT<br>TAEAVEQWSRSLLKLAAALEHHHHHHHH*
YZ031	MTGTEEKLVDYLKKVTAELQETRRQLRGALAASREPIAIVGMACRYPGGVRTPEALWRLVLDEQDAISGFPTNRGWDIDGIYHPDPDR<br>PGTCYAREGGFLHDAALFDAEFFGVSPREAQAMDPQQRLLLETAWEAFERAGIDPTSLRGSDTGVFAGVVHHDYATARVPETLEPYLV<br>TGLSGGVASGRIAYTFGFEGPAVTVDTACSSSLVALHQAAHALRSGECELALAGGVTIMATPRAFLSFSRQRGLSPDGRCRAFGAGADG<br>TGWAEGAGMLLVERLSDARRKGHPVLAVLRGSAVNQDGASNGLSAPNGPSQQRVIRKALAHAGLAARDVDVVEGHGTGTKLGDPIEA<br>QALLATYGQERDAGLPLHLGSMKSNVGHSQAAAGVGGVIKMIEAMRHGILPRTLHADEPTPHVDWSAGDIELLTRRRAWPETGRPRR<br>AAVSSFGISGTNAHVILEAPPEEPARDAAETRPEARSREAAEGRREAAGQGRTETGPGPSATPPEAPGRAALPHLLHASGRTLEAVQDL<br>LEQGRQHSQDLAFVSMLNDIAATPTAAMPFRGYTVLGVEGRVQEVQQVSTNKRPLWFICSGMGTQWRGMGLSLMRLDSFRESILRSD<br>EAVKPLGVKVSDLLLSTDERTFDDIVHAFVSLTAIQIALIDLLTSVGLKPDGIIGHSLGEVACGYADGCLSQREAVLAAYWRGQCIKDAHL<br>PPGSMAAVGLSWEECKQRCPAGVVPACHNSEDTVTISGPQAAVNEFVEQLKQEGVFAKEVRTGGLAFHSYFMEGIAPTLLQALKKVIR<br>EPRPRSARWLSTSIPEAQWQSSLARTSSAEYNVNNLVSPVLFQEALWHIPEHAVVLEIAPHALLQAVLKRGVKSSCTIIPLMKRDHKDN<br>LEFFLTNLGKVHLTGGTVDWSGLCAGGRLVDLPTYPFQRQHYWIEDQPLPPTAPRPGTAPSGTGTAAEGAAAAEVPLSERLARLTGAE<br>RLAAVRELVLAEASETLGHTGTLITADRTRQELGFDSLTAIELRNRISRTLGVRLPPTLVFDHEDLGEIASFVDARLDDAATGRSTGHGP<br>LGEDGSGLLTELFREAAAAGRLDDAVTLTEAAARMRRTFTDAEDPAVRRTPVWFGRGPARPTVVCLPSFSAIAGVHVYARFADAFGD<br>GWRVAALAHPGFVPGEPLPDSVDVLAELHARTVLDTVGADPFLLVGRSAGGWVAHEVAAVLERMGRAPDGVALLDTPARADDPRGH<br>AVMVGGMLERDSRLVTIDDYRLTAMGGYSRLFREWKPEPIAAATLLVHAATPYGADEARIASWDLPHQAVKVTGDHFTMLERHSAT<br>TAEAVEQWSRSLLKLAAALEHHHHHHHH*
YZ032	MTGTEEKLVDYLKKVTAELQETRRQLRGALAASREPIAIVGMACRYPGGVRTPEALWRLVLDEQDAISGFPTNRGWDIDGIYHPDPDR<br>PGTCYAREGGFLHDAALFDAEFFGVSPREAQAMDPQQRLLLETAWEAFERAGIDPTSLRGSDTGVFAGVVHHDYATARVPETLEPYLV<br>TGLSGGVASGRIAYTFGFEGPAVTVDTACSSSLVALHQAAHALRSGECELALAGGVTIMATPRAFLSFSRQRGLSPDGRCRAFGAGADG<br>TGWAEGAGMLLVERLSDARRKGHPVLAVLRGSAVNQDGASNGLSAPNGPSQQRVIRKALAHAGLAARDVDVVEGHGTGTKLGDPIEA<br>QALLATYGQERDAGLPLHLGSMKSNVGHSQAAAGVGGVIKMIEAMRHGILPRTLHADEPTPHVDWSAGDIELLTRRRAWPETGRPRR<br>AAVSSFGISGTNAHVILEAPPEEPARDAAETRPEARSREAAEGRREAAGQGRTETGPGPSATPPEAPGRARPPHLLHASGRTLEAVQDL<br>LEQGRQHSQDLAFVSMLNDIAATPTAAMPFRGYTVLGVEGRVQEVQQVSTNKRPLWFICSGMGTQWRGMGLSLMRLDSFRESILRSD<br>EAVKPLGVKVSDLLLSTDERTFDDIVHAFVSLTAIQIALIDLLTSVGLKPDGIIGHSLGEVACGYADGCLSQREAVLAAYWRGQCIKDAHL<br>PPGSMAAVGLSWEECKQRCPAGVVPACHNSEDTVTISGPQAAVNEFVEQLKQEGVFAKEVRTGGLAFHSYFMEGIAPTLLQALKKVIR<br>EPRPRSARWLSTSIPEAQWQSSLARTSSAEYNVNNLVSPVLFQEALWHIPEHAVVLEIAPHALLQAVLKRGVKSSCTIIPLMKRDHKDN<br>LEFFLTNLGQVHVDGGTVDWSGLCAGGRLVDLPTYPFQRQHYWIEDQPLPPTAPRPGTAPSGTGTAAEGAAAAEVPLSERLARLTGAE<br>RLAAVRELVLAEASETLGHTGTLITADRTRQELGFDSLTAIELRNRISRTLGVRLPPTLVFDHEDLGEIASFVDARLDDAATGRSTGHGP<br>LGEDGSGLLTELFREAAAAGRLDDAVTLTEAAARMRRTFTDAEDPAVRRTPVWFGRGPARPTVVCLPSFSAIAGVHVYARFADAFGD<br>GWRVAALAHPGFVPGEPLPDSVDVLAELHARTVLDTVGADPFLLVGRSAGGWVAHEVAAVLERMGRAPDGVALLDTPARADDPRGH<br>AVMVGGMLERDSRLVTIDDYRLTAMGGYSRLFREWKPEPIAAATLLVHAATPYGADEARIASWDLPHQAVKVTGDHFTMLERHSAT<br>TAEAVEQWSRSLLKLAAALEHHHHHHHH*

Construct	Amino acid sequence
YZ033	MTGTEEKLVDYLKKVTAELQETRRQLRGALAASREPIAIVGMACRYPGGVRTPEALWRLVLDEQDAISGFPTNRGWDIDGIYHPDPDR<br>PGTCYAREGGFLHDAALFDAEFFGVSPREAQAMDPQQRLLLETAWEAFERAGIDPTSLRGSDTGVFAGVVHHDYATARVPETLEPYLV<br>TGLSGGVASGRIAYTFGFEGPAVTVDTACSSSLVALHQAAHALRSGECELALAGGVTIMATPRAFLSFSRQRGLSPDGRCRAFGAGADG<br>TGWAEGAGMLLVERLSDARRKGHPVLAVLRGSAVNQDGASNGLSAPNGPSQQRVIRKALAHAGLAARDVDVVEGHGTGTKLGDPIEA<br>QALLATYGQERDAGLPLHLGSMKSNVGHSQAAAGVGGVIKMIEAMRHGILPRTLHADEPTPHVDWSAGDIELLTRRRAWPETGRPRR<br>AAVSSFGISGTNAHVILEAPPEEPARDAAETRPEARSREAAEGRREAAGQGRTETGPGPSATPPEAPGRARPPHLLHASGRTLEAVQDL<br>LEQGRQHSQDLAFVSMLNDIAATPTAAMPFRGYTVLGVEGRVQEVQQVSTNKRPLWFICSGMGTQWRGMGLSLMRLDSFRESILRSD<br>EAVKPLGVKVSDLLLSTDERTFDDIVHAFVSLTAIQIALIDLLTSVGLKPDGIIGHSLGEVACGYADGCLSQREAVLAAYWRGQCIKDAHL<br>PPGSMAAVGLSWEECKQRCPAGVVPACHNSEDTVTISGPQAAVNEFVEQLKQEGVFAKEVRTGGLAFHSYFMEGIAPTLLQALKKVIR<br>EPRPRSARWLSTSIPEAQWQSSLARTSSAEYNVNNLVSPVLFQEALWHIPEHAVVLEIAPHALLQAVLKRGVKSSCTIIPLMKRDHKDN<br>LEFFLTNLGKVHLDGGTVDWSGLCAGGRLVDLPTYPFQRQHYWIEDQPLPPTAPRPGTAPSGTGTAAEGAAAAEVPLSERLARLTGAE<br>RLAAVRELVLAEASETLGHTGTLITADRTRQELGFDSLTAIELRNRISRTLGVRLPPTLVFDHEDLGEIASFVDARLDDAATGRSTGHGP<br>LGEDGSGLLTELFREAAAAGRLDDAVTLTEAAARMRRTFTDAEDPAVRRTPVWFGRGPARPTVVCLPSFSAIAGVHVYARFADAFGD<br>GWRVAALAHPGFVPGEPLPDSVDVLAELHARTVLDTVGADPFLLVGRSAGGWVAHEVAAVLERMGRAPDGVALLDTPARADDPRGH<br>AVMVGGMLERDSRLVTIDDYRLTAMGGYSRLFREWKPEPIAAATLLVHAATPYGADEARIASWDLPHQAVKVTGDHFTMLERHSAT<br>TAEAVEQWSRSLLKLAAALEHHHHHHHH*
YZ034	MTGTEEKLVDYLKKVTAELQETRRQLRGALAASREPIAIVGMACRYPGGVRTPEALWRLVLDEQDAISGFPTNRGWDIDGIYHPDPDR<br>PGTCYAREGGFLHDAALFDAEFFGVSPREAQAMDPQQRLLLETAWEAFERAGIDPTSLRGSDTGVFAGVVHHDYATARVPETLEPYLV<br>TGLSGGVASGRIAYTFGFEGPAVTVDTACSSSLVALHQAAHALRSGECELALAGGVTIMATPRAFLSFSRQRGLSPDGRCRAFGAGADG<br>TGWAEGAGMLLVERLSDARRKGHPVLAVLRGSAVNQDGASNGLSAPNGPSQQRVIRKALAHAGLAARDVDVVEGHGTGTKLGDPIEA<br>QALLATYGQERDAGLPLHLGSMKSNVGHSQAAAGVGGVIKMIEAMRHGILPRTLHADEPTPHVDWSAGDIELLTRRRAWPETGRPRR<br>AAVSSFGISGTNAHVILEAPPEEPARDAAETRPEARSREAAEGRREAAGQGRTETGPGPSATPPEAPGRARPPHLLHASGRTLEAVQDL<br>LEQGRQHSQDLAFVSMLNDIAATPTAAMPFRGYTVLGVEGRVQEVQQVSTNKRPLWFICSGMGTQWRGMGLSLMRLDSFRESILRSD<br>EAVKPLGVKVSDLLLSTDERTFDDIVHAFVSLTAIQIALIDLLTSVGLKPDGIIGHSLGEVACGYADGCLSQREAVLAAYWRGQCIKDAHL<br>PPGSMAAVGLSWEECKQRCPAGVVPACHNSEDTVTISGPQAAVNEFVEQLKQEGVFAKEVRTGGLAFHSYFMEGIAPTLLQALKKVIR<br>EPRPRSARWLSTSIPEAQWQSSLARTSSAEYNVNNLVSPVLFQEALWHIPEHAVVLEIAPHALLQAVLKRGVKSSCTIIPLMKRDHKDN<br>LEFFLTNLGKVHLTGGTVDWSGLCAGGRLVDLPTYPFQRQHYWIEDQPLPPTAPRPGTAPSGTGTAAEGAAAAEVPLSERLARLTGAE<br>RLAAVRELVLAEASETLGHTGTLITADRTRQELGFDSLTAIELRNRISRTLGVRLPPTLVFDHEDLGEIASFVDARLDDAATGRSTGHGP<br>LGEDGSGLLTELFREAAAAGRLDDAVTLTEAAARMRRTFTDAEDPAVRRTPVWFGRGPARPTVVCLPSFSAIAGVHVYARFADAFGD<br>GWRVAALAHPGFVPGEPLPDSVDVLAELHARTVLDTVGADPFLLVGRSAGGWVAHEVAAVLERMGRAPDGVALLDTPARADDPRGH<br>AVMVGGMLERDSRLVTIDDYRLTAMGGYSRLFREWKPEPIAAATLLVHAATPYGADEARIASWDLPHQAVKVTGDHFTMLERHSAT<br>TAEAVEQWSRSLLKLAAALEHHHHHHHH*
YZ035	MTGTEEKLVDYLKKVTAELQETRRQLRGALAASREPIAIVGMACRYPGGVRTPEALWRLVLDEQDAISGFPTNRGWDIDGIYHPDPDR<br>PGTCYAREGGFLHDAALFDAEFFGVSPREAQAMDPQQRLLLETAWEAFERAGIDPTSLRGSDTGVFAGVVHHDYATARVPETLEPYLV<br>TGLSGGVASGRIAYTFGFEGPAVTVDTACSSSLVALHQAAHALRSGECELALAGGVTIMATPRAFLSFSRQRGLSPDGRCRAFGAGADG<br>TGWAEGAGMLLVERLSDARRKGHPVLAVLRGSAVNQDGASNGLSAPNGPSQQRVIRKALAHAGLAARDVDVVEGHGTGTKLGDPIEA<br>QALLATYGQERDAGLPLHLGSMKSNVGHSQAAAGVGGVIKMIEAMRHGILPRTLHADEPTPHVDWSAGDIELLTRRRAWPETGRPRR<br>AAVSSFGISGTNAHVILEAPPEEPARDAAETRPEARSREAAEGRREAAGQGRTETGPGPSATPPEAPGRARPPVLLHASGRTLEAVQDL<br>LEQGRQHSQDLAFVSMLNDIAATPTAAMPFRGYTVLGVEGRVQEVQQVSTNKRPLWFICSGMGTQWRGMGLSLMRLDSFRESILRSD<br>EAVKPLGVKVSDLLLSTDERTFDDIVHAFVSLTAIQIALIDLLTSVGLKPDGIIGHSLGEVACGYADGCLSQREAVLAAYWRGQCIKDAHL<br>PPGSMAAVGLSWEECKQRCPAGVVPACHNSEDTVTISGPQAAVNEFVEQLKQEGVFAKEVRTGGLAFHSYFMEGIAPTLLQALKKVIR<br>EPRPRSARWLSTSIPEAQWQSSLARTSSAEYNVNNLVSPVLFQEALWHIPEHAVVLEIAPHALLQAVLKRGVKSSCTIIPLMKRDHKDN<br>LEFFLTNLGQVHVDGGTVDWSGLCAGGRLVDLPTYPFQRQHYWIEDQPLPPTAPRPGTAPSGTGTAAEGAAAAEVPLSERLARLTGAE<br>RLAAVRELVLAEASETLGHTGTLITADRTRQELGFDSLTAIELRNRISRTLGVRLPPTLVFDHEDLGEIASFVDARLDDAATGRSTGHGP<br>LGEDGSGLLTELFREAAAAGRLDDAVTLTEAAARMRRTFTDAEDPAVRRTPVWFGRGPARPTVVCLPSFSAIAGVHVYARFADAFGD<br>GWRVAALAHPGFVPGEPLPDSVDVLAELHARTVLDTVGADPFLLVGRSAGGWVAHEVAAVLERMGRAPDGVALLDTPARADDPRGH<br>AVMVGGMLERDSRLVTIDDYRLTAMGGYSRLFREWKPEPIAAATLLVHAATPYGADEARIASWDLPHQAVKVTGDHFTMLERHSAT<br>TAEAVEQWSRSLLKLAAALEHHHHHHHH*

Construct	Amino acid sequence
YZ036	MTGTEEKLVDYLKKVTAELQETRRQLRGALAASREPIAIVGMACRYPGGVRTPEALWRLVLDEQDAISGFPTNRGWDIDGIYHPDPDR<br>PGTCYAREGGFLHDAALFDAEFFGVSPREAQAMDPQQRLLLETAWEAFERAGIDPTSLRGSDTGVFAGVVHHDYATARVPETLEPYLV<br>TGLSGGVASGRIAYTFGFEGPAVTVDTACSSSLVALHQAAHALRSGECELALAGGVTIMATPRAFLSFSRQRGLSPDGRCRAFGAGADG<br>TGWAEGAGMLLVERLSDARRKGHPVLAVLRGSAVNQDGASNGLSAPNGPSQQRVIRKALAHAGLAARDVDVVEGHGTGTKLGDPIEA<br>QALLATYGQERDAGLPLHLGSMKSNVGHSQAAAGVGGVIKMIEAMRHGILPRTLHADEPTPHVDWSAGDIELLTRRRAWPETGRPRR<br>AAVSSFGISGTNAHVILEAPPEEPARDAAETRPEARSREAAEGRREAAGQGRTETGPGPSATPPEAPGRARPPVLLHASGRTLEAVQDL<br>LEQGRQHSQDLAFVSMLNDIAATPTAAMPFRGYTVLGVEGRVQEVQQVSTNKRPLWFICSGMGTQWRGMGLSLMRLDSFRESILRSD<br>EAVKPLGVKVSDLLLSTDERTFDDIVHAFVSLTAIQIALIDLLTSVGLKPDGIIGHSLGEVACGYADGCLSQREAVLAAYWRGQCIKDAHL<br>PPGSMAAVGLSWEECKQRCPAGVVPACHNSEDTVTISGPQAAVNEFVEQLKQEGVFAKEVRTGGLAFHSYFMEGIAPTLLQALKKVIR<br>EPRPRSARWLSTSIPEAQWQSSLARTSSAEYNVNNLVSPVLFQEALWHIPEHAVVLEIAPHALLQAVLKRGVKSSCTIIPLMKRDHKDN<br>LEFFLTNLGKVHLDGGTVDWSGLCAGGRLVDLPTYPFQRQHYWIEDQPLPPTAPRPGTAPSGTGTAAEGAAAAEVPLSERLARLTGAE<br>RLAAVRELVLAEASETLGHTGTLITADRTRQELGFDSLTAIELRNRISRTLGVRLPPTLVFDHEDLGEIASFVDARLDDAATGRSTGHGP<br>LGEDGSGLLTELFREAAAAGRLDDAVTLTEAAARMRRTFTDAEDPAVRRTPVWFGRGPARPTVVCLPSFSAIAGVHVYARFADAFGD<br>GWRVAALAHPGFVPGEPLPDSVDVLAELHARTVLDTVGADPFLLVGRSAGGWVAHEVAAVLERMGRAPDGVALLDTPARADDPRGH<br>AVMVGGMLERDSRLVTIDDYRLTAMGGYSRLFREWKPEPIAAATLLVHAATPYGADEARIASWDLPHQAVKVTGDHFTMLERHSAT<br>TAEAVEQWSRSLLKLAAALEHHHHHHHH*

Table S7. Amino acid sequences of MAT-swapped VEMS M0-M1* and split VEMS M1* constructs.

Construct	Amino acid sequence
YZ038	MSAWSHPQFEKGGGSGGGSGGSAWSHPQFEKGAGSVTQVDDIRPLSAVLRAHALDRPDKVAFADEERHVTYARLAERTGRLAGHLAG<br>HGLRRGDRVAILLGNSVTTVESYLAVTRAAAVGVPVNPQSSDAELAHQLDDSGARFVITDGPHLDQVERLRATREGIRVVLARGYGPGG<br>GPHGPGPEAPASEGAPALDGTSAPDGAPGPDGPLSFEELAGSEPAQAPRDDLGLDEPAWILYTSGTTGAAKGVVSHQRACLWSVRSSY<br>QGVLGLGPDDRLLWPLPLFHSLAHILCVLGVTVSGATARILPGFGARDVLDALRAEPCTMLLGVPSMYLRLVAAVEAVEEAGGSVPKPR<br>LCVVTGAATGPELASAVERVLGAPLVNSYGATETCGAITLSRPDSERPPGTVGTAVPGSELRLVDPRTGRDARTGDEGEVLVRSPGLLL<br>RYHDRPEETAAALRDGWYRTGDLARRDAAGRLTITGRVKELVIRAGENIQPGEIEDVLRTVPGIADAAVTGRPDEALGEVPVAYVVPA<br>AGGWSPAEALAACRERLSYFKVPAELYEIAALPVTGSGKVTRRALPGLPARLRALGTGHHERLWRTTWVQCPAPDAAAEPAGAARW<br>AVIGAEVPAFAEAVRRRGGVAETFPDPAAARAAGPFDGHLLTAPAPDPDAPAEAAAESPLRTAEGPPAARTLLLTRSGVSTGPDDTPD<br>PTASAVRARLLARGGDGTVLVDLGPEDPRPTGGLAEAEAEAEAEAEADGAFAGAAARDADTGDAPGSDVPSPDVEAWLAVLSAPAGE<br>TEFALRSGAALVPRLARVRAAGEVALPSGGPGAALVTGAGTGISRVLATHLVVNHGVRDLVLADAGEEGDPGAVATLAADLTGLGATV<br>TVRTGRPDTAERASALLGALGPDRPFALVVHPVTAPADAEAAGRLLDLTRRSEGTTLVLCGRAAPGDPSEAAAAAHAAALTHDVAHA<br>TFLAFGPVAPDAPQPGLAALTAREAGDALDAVRATAGGPALVLRTDPWTLRAPGAERLRRMLPRYAEDTAAPAALDEGLRERLGVLP<br>GRARQRALTALVREEAAGVLGLDAPRRIDAGLAFTRLGLTSLTAVALRDRLAARTGLRLPVTLAFDHPTPAAVAAVLDGELFGARPAT<br>DAARRTSEDAGTSHRTGPRHDAHDPVVVIGMSCRYPGGIASPDDLWRFVAEGRDAIGPFPTDRGWDLERLLSGDGTAPGTSATGSGG<br>FLDAPGDFDAAFFGVSPREALAMDPQQRLLLEVGWEAVERAGIDPTSLRHTDTGVYVGLMFHDYAQHTGDLPEDLERLLGTGTAGSV<br>ASGRLAYTLGLNGPALTVDTACSSSLVAVHLAAQALRRGDCSLALAGGVAVMATPSTFVEFSRQHGLAADGRCKAFGEGADGTGWSEG<br>VGVVALERLSDARRHGHPVLAVIRGSAVNQDGASNGLTAPSGPAQQQVIRRALAAAGLTPGDVDAVEAHGTGTVLGDPIEANALLATY<br>GRGRDPERPLLLGSFKSNIGHAQSAAGVGAVIKMVQAIRYGELPRTLHAERPTPAVDWSGGAVRLLREHTPWPVTGDRRRAAVSSFGV<br>SGTNVHLVLEQAPEPDAPPALPPAVHPDTMPVLLSARTPSALRRQAERVLDAVRADPGLPVPDLAAALATTRTAFPRRAAFVVEGRA<br>ELERRLRSFADGEDGGAEPDEVPANKRPLWFICSGMGTQWRGMGLSLMRLDSFRESILRSDEAVKPLGVKVSDLLLSTDERTFDDIVH<br>AFVSLTAIQIALIDLLTSVGLKPDGIIGHSLGEVACGYADGCLSQREAVLAAYWRGQCIKDAHLPPGSMAAVGLSWEECKQRCPAGVVPA<br>CHNSEDTVTISGPQAAVNEFVEQLKQEGVFAKEVRTGGLAFHSYFMEGIAPTLLQALKKVIREPRPRSARWLSTSIPEAQWQSSLARTS<br>SAEYNVNNLVSPVLFQEALWHIPEHAVVLEIAPHALLQAVLKRGVKSSCTIIPLMKRDHKDNLEFFLTNLGKVHLTGVPVDWREFFAG<br>GPTRPVDLPVYPFEHRRYWLASAPTRTGGATSVPEAPAPPTGDTPGPDRLRADLAPLTDDERERRLTDLVRGEIAAVAGFDGPHEIEA<br>HRSMTDLGLDSVSAVDLSTRLGARTGLDLPASLAFDHPTSTAIARHLLAALRTQGLTPESGQDAGTDGGTEGGKHAGIRAAEGTVFGA<br>LARLEASVDRDRPDAAARDRLVERLRALTARLTEGPAGGPSADDTSIGDRIGEATTVDQLFDLIDNELKGPLEHHHHHHHH*
YZ040	MSAWSHPQFEKGGGSGGGSGGSAWSHPQFEKGAGSVTQVDDIRPLSAVLRAHALDRPDKVAFADEERHVTYARLAERTGRLAGHLAG<br>HGLRRGDRVAILLGNSVTTVESYLAVTRAAAVGVPVNPQSSDAELAHQLDDSGARFVITDGPHLDQVERLRATREGIRVVLARGYGPGG<br>GPHGPGPEAPASEGAPALDGTSAPDGAPGPDGPLSFEELAGSEPAQAPRDDLGLDEPAWILYTSGTTGAAKGVVSHQRACLWSVRSSY<br>QGVLGLGPDDRLLWPLPLFHSLAHILCVLGVTVSGATARILPGFGARDVLDALRAEPCTMLLGVPSMYLRLVAAVEAVEEAGGSVPKPR<br>LCVVTGAATGPELASAVERVLGAPLVNSYGATETCGAITLSRPDSERPPGTVGTAVPGSELRLVDPRTGRDARTGDEGEVLVRSPGLLL<br>RYHDRPEETAAALRDGWYRTGDLARRDAAGRLTITGRVKELVIRAGENIQPGEIEDVLRTVPGIADAAVTGRPDEALGEVPVAYVVPA<br>AGGWSPAEALAACRERLSYFKVPAELYEIAALPVTGSGKVTRRALPGLPARLRALGTGHHERLWRTTWVQCPAPDAAAEPAGAARW<br>AVIGAEVPAFAEAVRRRGGVAETFPDPAAARAAGPFDGHLLTAPAPDPDAPAEAAAESPLRTAEGPPAARTLLLTRSGVSTGPDDTPD<br>PTASAVRARLLARGGDGTVLVDLGPEDPRPTGGLAEAEAEAEAEAEADGAFAGAAARDADTGDAPGSDVPSPDVEAWLAVLSAPAGE<br>TEFALRSGAALVPRLARVRAAGEVALPSGGPGAALVTGAGTGISRVLATHLVVNHGVRDLVLADAGEEGDPGAVATLAADLTGLGATV<br>TVRTGRPDTAERASALLGALGPDRPFALVVHPVTAPADAEAAGRLLDLTRRSEGTTLVLCGRAAPGDPSEAAAAAHAAALTHDVAHA<br>TFLAFGPVAPDAPQPGLAALTAREAGDALDAVRATAGGPALVLRTDPWTLRAPGAERLRRMLPRYAEDTAAPAALDEGLRERLGVLP<br>GRARQRALTALVREEAAGVLGLDAPRRIDAGLAFTRLGLTSLTAVALRDRLAARTGLRLPVTLAFDHPTPAAVAAVLDGELFGARPAT<br>DAARRTSEDAGTSHRTGPRHDAHDPVVVIGMSCRYPGGIASPDDLWRFVAEGRDAIGPFPTDRGWDLERLLSGDGTAPGTSATGSGG<br>FLDAPGDFDAAFFGVSPREALAMDPQQRLLLEVGWEAVERAGIDPTSLRHTDTGVYVGLMFHDYAQHTGDLPEDLERLLGTGTAGSV<br>ASGRLAYTLGLNGPALTVDTACSSSLVAVHLAAQALRRGDCSLALAGGVAVMATPSTFVEFSRQHGLAADGRCKAFGEGADGTGWSEG<br>VGVVALERLSDARRHGHPVLAVIRGSAVNQDGASNGLTAPSGPAQQQVIRRALAAAGLTPGDVDAVEAHGTGTVLGDPIEANALLATY<br>GRGRDPERPLLLGSFKSNIGHAQSAAGVGAVIKMVQAIRYGELPRTLHAERPTPAVDWSGGAVRLLREHTPWPVTGDRRRAAVSSFGV

Construct	Amino acid sequence
	SGTNVHLVLEQAPEPDAPPTAHAALPHLLHASGRTLEAVQDLLEQGRQHSQDLAFVSMLNDIAATPTAAMPFRGYTVLGVEGRVQEV<br>QQVSTNKRPLWFICSGMGTQWRGMGLSLMRLDSFRESILRSDEAVKPLGVKVSDLLLSTDERTFDDIVHAFVSLTAIQIALIDLLTSVGL<br>KPDGIIGHSLGEVACGYADGCLSQREAVLAAYWRGQCIKDAHLPPGSMAAVGLSWEECKQRCPAGVVPACHNSEDTVTISGPQAAVNE<br>FVEQLKQEGVFAKEVRTGGLAFHSYFMEGIAPTLLQALKKVIREPRPRSARWLSTSIPEAQWQSSLARTSSAEYNVNNLVSPVLFQEAL<br>WHIPEHAVVLEIAPHALLQAVLKRGVKSSCTIIPLMKRDHKDNLEFFLTNLGKVHLTGINVNPNALFPPVEFPAPRGTPLILPVYPFEHR<br>RYWLASAPTRTGGATSVPEAPAPPTGDTPGPDRLRADLAPLTDDERERRLTDLVRGEIAAVAGFDGPHEIEAHRSMTDLGLDSVSAVD<br>LSTRLGARTGLDLPASLAFDHPTSTAIARHLLAALRTQGLTPESGQDAGTDGGTEGGKHAGIRAAEGTVFGALARLEASVDRDRPDAA<br>ARDRLVERLRALTARLTEGPAGGPSADDTSIGDRIGEATTVDQLFDLIDNELKGPLEHHHHHHHH*
YZ041	MSAWSHPQFEKGGGSGGGSGGSAWSHPQFEKGAGSVTQVDDIRPLSAVLRAHALDRPDKVAFADEERHVTYARLAERTGRLAGHLAG<br>HGLRRGDRVAILLGNSVTTVESYLAVTRAAAVGVPVNPQSSDAELAHQLDDSGARFVITDGPHLDQVERLRATREGIRVVLARGYGPGG<br>GPHGPGPEAPASEGAPALDGTSAPDGAPGPDGPLSFEELAGSEPAQAPRDDLGLDEPAWILYTSGTTGAAKGVVSHQRACLWSVRSSY<br>QGVLGLGPDDRLLWPLPLFHSLAHILCVLGVTVSGATARILPGFGARDVLDALRAEPCTMLLGVPSMYLRLVAAVEAVEEAGGSVPKPR<br>LCVVTGAATGPELASAVERVLGAPLVNSYGATETCGAITLSRPDSERPPGTVGTAVPGSELRLVDPRTGRDARTGDEGEVLVRSPGLLL<br>RYHDRPEETAAALRDGWYRTGDLARRDAAGRLTITGRVKELVIRAGENIQPGEIEDVLRTVPGIADAAVTGRPDEALGEVPVAYVVPA<br>AGGWSPAEALAACRERLSYFKVPAELYEIAALPVTGSGKVTRRALPGLPARLRALGTGHHERLWRTTWVQCPAPDAAAEPAGAARW<br>AVIGAEVPAFAEAVRRRGGVAETFPDPAAARAAGPFDGHLLTAPAPDPDAPAEAAAESPLRTAEGPPAARTLLLTRSGVSTGPDDTPD<br>PTASAVRARLLARGGDGTVLVDLGPEDPRPTGGLAEAEAEAEAEAEADGAFAGAAARDADTGDAPGSDVPSPDVEAWLAVLSAPAGE<br>TEFALRSGAALVPRLARVRAAGEVALPSGGPGAALVTGAGTGISRVLATHLVVNHGVRDLVLADAGEEGDPGAVATLAADLTGLGATV<br>TVRTGRPDTAERASALLGALGPDRPFALVVHPVTAPADAEAAGRLLDLTRRSEGTTLVLCGRAAPGDPSEAAAAAHAAALTHDVAHA<br>TFLAFGPVAPDAPQPGLAALTAREAGDALDAVRATAGGPALVLRTDPWTLRAPGAERLRRMLPRYAEDTAAPAALDEGLRERLGVLP<br>GRARQRALTALVREEAAGVLGLDAPRRIDAGLAFTRLGLTSLTAVALRDRLAARTGLRLPVTLAFDHPTPAAVAAVLDGELFGARPAT<br>DAARRTSEDAGTSHRTGPRHDAHDPVVVIGMSCRYPGGIASPDDLWRFVAEGRDAIGPFPTDRGWDLERLLSGDGTAPGTSATGSGG<br>FLDAPGDFDAAFFGVSPREALAMDPQQRLLLEVGWEAVERAGIDPTSLRHTDTGVYVGLMFHDYAQHTGDLPEDLERLLGTGTAGSV<br>ASGRLAYTLGLNGPALTVDTACSSSLVAVHLILQPNTRQAPAPTAHAALPHLLHASGRTLEAVQDLLEQGRQHSQDLAFVSMLNDIAA<br>TPTAAMPFRGYTVLGVEGRVQEVQQVSTNKRPLWFICSGMGTQWRGMGLSLMRLDSFRESILRSDEAVKPLGVKVSDLLLSTDERTF<br>DDIVHAFVSLTAIQIALIDLLTSVGLKPDGIIGHSLGEVACGYADGCLSQREAVLAAYWRGQCIKDAHLPPGSMAAVGLSWEECKQRCPA<br>GVVPACHNSEDTVTISGPQAAVNEFVEQLKQEGVFAKEVRTGGLAFHSYFMEGIAPTLLQALKKVIREPRPRSARWLSTSIPEAQWQSS<br>LARTSSAEYNVNNLVSPVLFQEALWHIPEHAVVLEIAPHALLQAVLKRGVKSSCTIIPLMKRDHKDNLEFFLTNLGKVHLTGINVNPNA<br>LFPPVEFPAPRGTPVDLPVYPFEHRRYWLASAPTRTGGATSVPEAPAPPTGDTPGPDRLRADLAPLTDDERERRLTDLVRGEIAAVAG<br>FDGPHEIEAHRSMTDLGLDSVSAVDLSTRLGARTGLDLPASLAFDHPTSTAIARHLLAALRTQGLTPESGQDAGTDGGTEGGKHAGIRA<br>AEGTVFGALARLEASVDRDRPDAAARDRLVERLRALTARLTEGPAGGPSADDTSIGDRIGEATTVDQLFDLIDNELKGPLEHHHHHHH<br>H*
YZ042	MSAWSHPQFEKGGGSGGGSGGSAWSHPQFEKGAGSVTQVDDIRPLSAVLRAHALDRPDKVAFADEERHVTYARLAERTGRLAGHLAG<br>HGLRRGDRVAILLGNSVTTVESYLAVTRAAAVGVPVNPQSSDAELAHQLDDSGARFVITDGPHLDQVERLRATREGIRVVLARGYGPGG<br>GPHGPGPEAPASEGAPALDGTSAPDGAPGPDGPLSFEELAGSEPAQAPRDDLGLDEPAWILYTSGTTGAAKGVVSHQRACLWSVRSSY<br>QGVLGLGPDDRLLWPLPLFHSLAHILCVLGVTVSGATARILPGFGARDVLDALRAEPCTMLLGVPSMYLRLVAAVEAVEEAGGSVPKPR<br>LCVVTGAATGPELASAVERVLGAPLVNSYGATETCGAITLSRPDSERPPGTVGTAVPGSELRLVDPRTGRDARTGDEGEVLVRSPGLLL<br>RYHDRPEETAAALRDGWYRTGDLARRDAAGRLTITGRVKELVIRAGENIQPGEIEDVLRTVPGIADAAVTGRPDEALGEVPVAYVVPA<br>AGGWSPAEALAACRERLSYFKVPAELYEIAALPVTGSGKVTRRALPGLPARLRALGTGHHERLWRTTWVQCPAPDAAAEPAGAARW<br>AVIGAEVPAFAEAVRRRGGVAETFPDPAAARAAGPFDGHLLTAPAPDPDAPAEAAAESPLRTAEGPPAARTLLLTRSGVSTGPDDTPD<br>PTASAVRARLLARGGDGTVLVDLGPEDPRPTGGLAEAEAEAEAEAEADGAFAGAAARDADTGDAPGSDVPSPDVEAWLAVLSAPAGE<br>TEFALRSGAALVPRLARVRAAGEVALPSGGPGAALVTGAGTGISRVLATHLVVNHGVRDLVLADAGEEGDPGAVATLAADLTGLGATV<br>TVRTGRPDTAERASALLGALGPDRPFALVVHPVTAPADAEAAGRLLDLTRRSEGTTLVLCGRAAPGDPSEAAAAAHAAALTHDVAHA<br>TFLAFGPVAPDAPQPGLAALTAREAGDALDAVRATAGGPALVLRTDPWTLRAPGAERLRRMLPRYAEDTAAPAALDEGLRERLGVLP<br>GRARQRALTALVREEAAGVLGLDAPRRIDAGLAFTRLGLTSLTAVALRDRLAARTGLRLPVTLAFDHPTPAAVAAVLDGELFGARPAT<br>DAARRTSEDAGTSHRTGPRHDAHDPVVVIGMSCRYPGGIASPDDLWRFVAEGRDAIGPFPTDRGWDLERLLSGDGTAPGTSATGSGG<br>FLDAPGDFDAAFFGVSPREALAMDPQQRLLLEVGWEAVERAGIDPTSLRHTDTGVYVGLMFHDYAQHTGDLPEDLERLLGTGTAGSV<br>ASGRLAYTLGLNGPALTVDTACSSSLVAVHLAAQALRRGDCSLALAGGVAVMATPSTFVEFSRQHGLAADGRCKAFGEGADGTGWSEG<br>VGVVALERLSDARRHGHPVLAVIRGSAVNQDGASNGLTAPSGPAQQQVIRRALAAAGLTPGDVDAVEAHGTGTVLGDPIEANALLATY<br>GRGRDPERPLLLGSFKSNIGHAQSAAGVGAVIKMVQAIRYGELPRTLHAERPTPAVDWSGGAVRLLREHTPWPVTGDRRRAAVSSFGV<br>SGTNVHLVLEPNTRQAPAPTAHAALPHLLHASGRTLEAVQDLLEQGRQHSQDLAFVSMLNDIAATPTAAMPFRGYTVLGVEGRVQEV<br>QQVSTNKRPLWFICSGMGTQWRGMGLSLMRLDSFRESILRSDEAVKPLGVKVSDLLLSTDERTFDDIVHAFVSLTAIQIALIDLLTSVGL<br>KPDGIIGHSLGEVACGYADGCLSQREAVLAAYWRGQCIKDAHLPPGSMAAVGLSWEECKQRCPAGVVPACHNSEDTVTISGPQAAVNE<br>FVEQLKQEGVFAKEVRTGGLAFHSYFMEGIAPTLLQALKKVIREPRPRSARWLSTSIPEAQWQSSLARTSSAEYNVNNLVSPVLFQEAL<br>WHIPEHAVVLEIAPHALLQAVLKRGVKSSCTIIPLMKRDHKDNLEFFLTNLGKVHLTGINVNPNALFPPVEFPAPRGTPLDLPVYPFEH<br>RRYWLASAPTRTGGATSVPEAPAPPTGDTPGPDRLRADLAPLTDDERERRLTDLVRGEIAAVAGFDGPHEIEAHRSMTDLGLDSVSAV<br>DLSTRLGARTGLDLPASLAFDHPTSTAIARHLLAALRTQGLTPESGQDAGTDGGTEGGKHAGIRAAEGTVFGALARLEASVDRDRPDA

Construct	Amino acid sequence
	AARDRLVERLRALTARLTEGPAGGPSADDTSIGDRIGEATTVDQLFDLIDNELKGPLEHHHHHHHH*
YZ043	MSAWSHPQFEKGGGSGGGSGGSAWSHPQFEKGAGSVTQVDDIRPLSAVLRAHALDRPDKVAFADEERHVTYARLAERTGRLAGHLAG<br>HGLRRGDRVAILLGNSVTTVESYLAVTRAAAVGVPVNPQSSDAELAHQLDDSGARFVITDGPHLDQVERLRATREGIRVVLARGYGPGG<br>GPHGPGPEAPASEGAPALDGTSAPDGAPGPDGPLSFEELAGSEPAQAPRDDLGLDEPAWILYTSGTTGAAKGVVSHQRACLWSVRSSY<br>QGVLGLGPDDRLLWPLPLFHSLAHILCVLGVTVSGATARILPGFGARDVLDALRAEPCTMLLGVPSMYLRLVAAVEAVEEAGGSVPKPR<br>LCVVTGAATGPELASAVERVLGAPLVNSYGATETCGAITLSRPDSERPPGTVGTAVPGSELRLVDPRTGRDARTGDEGEVLVRSPGLLL<br>RYHDRPEETAAALRDGWYRTGDLARRDAAGRLTITGRVKELVIRAGENIQPGEIEDVLRTVPGIADAAVTGRPDEALGEVPVAYVVPA<br>AGGWSPAEALAACRERLSYFKVPAELYEIAALPVTGSGKVTRRALPGLPARLRALGTGHHERLWRTTWVQCPAPDAAAEPAGAARW<br>AVIGAEVPAFAEAVRRRGGVAETFPDPAAARAAGPFDGHLLTAPAPDPDAPAEAAAESPLRTAEGPPAARTLLLTRSGVSTGPDDTPD<br>PTASAVRARLLARGGDGTVLVDLGPEDPRPTGGLAEAEAEAEAEAEADGAFAGAAARDADTGDAPGSDVPSPDVEAWLAVLSAPAGE<br>TEFALRSGAALVPRLARVRAAGEVALPSGGPGAALVTGAGTGISRVLATHLVVNHGVRDLVLADAGEEGDPGAVATLAADLTGLGATV<br>TVRTGRPDTAERASALLGALGPDRPFALVVHPVTAPADAEAAGRLLDLTRRSEGTTLVLCGRAAPGDPSEAAAAAHAAALTHDVAHA<br>TFLAFGPVAPDAPQPGLAALTAREAGDALDAVRATAGGPALVLRTDPWTLRAPGAERLRRMLPRYAEDTAAPAALDEGLRERLGVLP<br>GRARQRALTALVREEAAGVLGLDAPRRIDAGLAFTRLGLTSLTAVALRDRLAARTGLRLPVTLAFDHPTPAAVAAVLDGELFGARPAT<br>DAARRTSEDAGTSHRTGPRHDAHDPVVVIGMSCRYPGGIASPDDLWRFVAEGRDAIGPFPTDRGWDLERLLSGDGTAPGTSATGSGG<br>FLDAPGDFDAAFFGVSPREALAMDPQQRLLLEVGWEAVERAGIDPTSLRHTDTGVYVGLMFHDYAQHTGDLPEDLERLLGTGTAGSV<br>ASGRLAYTLGLNGPALTVDTACSSSLVAVHLAAQALRRGDCSLALAGGVAVMATPSTFVEFSRQHGLAADGRCKAFGEGADGTGWSEG<br>VGVVALERLSDARRHGHPVLAVIRGSAVNQDGASNGLTAPSGPAQQQVIRRALAAAGLTPGDVDAVEAHGTGTVLGDPIEANALLATY<br>GRGRDPERPLLLGSFKSNIGHAQSAAGVGAVIKMVQAIRYGELPRTLHAERPTPAVDWSGGAVRLLREHTPWPVTGDRRRAAVSSFGV<br>SGTNVHLVLEQAPEPDAPPALPTAHAALPHLLHASGRTLEAVQDLLEQGRQHSQDLAFVSMLNDIAATPTAAMPFRGYTVLGVEGRV<br>QEVQQVSTNKRPLWFICSGMGTQWRGMGLSLMRLDSFRESILRSDEAVKPLGVKVSDLLLSTDERTFDDIVHAFVSLTAIQIALIDLLTS<br>VGLKPDGIIGHSLGEVACGYADGCLSQREAVLAAYWRGQCIKDAHLPPGSMAAVGLSWEECKQRCPAGVVPACHNSEDTVTISGPQAA<br>VNEFVEQLKQEGVFAKEVRTGGLAFHSYFMEGIAPTLLQALKKVIREPRPRSARWLSTSIPEAQWQSSLARTSSAEYNVNNLVSPVLFQ<br>EALWHIPEHAVVLEIAPHALLQAVLKRGVKSSCTIIPLMKRDHKDNLEFFLTNLGKVHLRGVPVDWREFFAGGPTRPVDLPVYPFEHR<br>RYWLASAPTRTGGATSVPEAPAPPTGDTPGPDRLRADLAPLTDDERERRLTDLVRGEIAAVAGFDGPHEIEAHRSMTDLGLDSVSAVD<br>LSTRLGARTGLDLPASLAFDHPTSTAIARHLLAALRTQGLTPESGQDAGTDGGTEGGKHAGIRAAEGTVFGALARLEASVDRDRPDAA<br>ARDRLVERLRALTARLTEGPAGGPSADDTSIGDRIGEATTVDQLFDLIDNELKGPLEHHHHHHHH*
YZ044	MSAWSHPQFEKGGGSGGGSGGSAWSHPQFEKGAGSVTQVDDIRPLSAVLRAHALDRPDKVAFADEERHVTYARLAERTGRLAGHLAG<br>HGLRRGDRVAILLGNSVTTVESYLAVTRAAAVGVPVNPQSSDAELAHQLDDSGARFVITDGPHLDQVERLRATREGIRVVLARGYGPGG<br>GPHGPGPEAPASEGAPALDGTSAPDGAPGPDGPLSFEELAGSEPAQAPRDDLGLDEPAWILYTSGTTGAAKGVVSHQRACLWSVRSSY<br>QGVLGLGPDDRLLWPLPLFHSLAHILCVLGVTVSGATARILPGFGARDVLDALRAEPCTMLLGVPSMYLRLVAAVEAVEEAGGSVPKPR<br>LCVVTGAATGPELASAVERVLGAPLVNSYGATETCGAITLSRPDSERPPGTVGTAVPGSELRLVDPRTGRDARTGDEGEVLVRSPGLLL<br>RYHDRPEETAAALRDGWYRTGDLARRDAAGRLTITGRVKELVIRAGENIQPGEIEDVLRTVPGIADAAVTGRPDEALGEVPVAYVVPA<br>AGGWSPAEALAACRERLSYFKVPAELYEIAALPVTGSGKVTRRALPGLPARLRALGTGHHERLWRTTWVQCPAPDAAAEPAGAARW<br>AVIGAEVPAFAEAVRRRGGVAETFPDPAAARAAGPFDGHLLTAPAPDPDAPAEAAAESPLRTAEGPPAARTLLLTRSGVSTGPDDTPD<br>PTASAVRARLLARGGDGTVLVDLGPEDPRPTGGLAEAEAEAEAEAEADGAFAGAAARDADTGDAPGSDVPSPDVEAWLAVLSAPAGE<br>TEFALRSGAALVPRLARVRAAGEVALPSGGPGAALVTGAGTGISRVLATHLVVNHGVRDLVLADAGEEGDPGAVATLAADLTGLGATV<br>TVRTGRPDTAERASALLGALGPDRPFALVVHPVTAPADAEAAGRLLDLTRRSEGTTLVLCGRAAPGDPSEAAAAAHAAALTHDVAHA<br>TFLAFGPVAPDAPQPGLAALTAREAGDALDAVRATAGGPALVLRTDPWTLRAPGAERLRRMLPRYAEDTAAPAALDEGLRERLGVLP<br>GRARQRALTALVREEAAGVLGLDAPRRIDAGLAFTRLGLTSLTAVALRDRLAARTGLRLPVTLAFDHPTPAAVAAVLDGELFGARPAT<br>DAARRTSEDAGTSHRTGPRHDAHDPVVVIGMSCRYPGGIASPDDLWRFVAEGRDAIGPFPTDRGWDLERLLSGDGTAPGTSATGSGG<br>FLDAPGDFDAAFFGVSPREALAMDPQQRLLLEVGWEAVERAGIDPTSLRHTDTGVYVGLMFHDYAQHTGDLPEDLERLLGTGTAGSV<br>ASGRLAYTLGLNGPALTVDTACSSSLVAVHLAAQALRRGDCSLALAGGVAVMATPSTFVEFSRQHGLAADGRCKAFGEGADGTGWSEG<br>VGVVALERLSDARRHGHPVLAVIRGSAVNQDGASNGLTAPSGPAQQQVIRRALAAAGLTPGDVDAVEAHGTGTVLGDPIEANALLATY<br>GRGRDPERPLLLGSFKSNIGHAQSAAGVGAVIKMVQAIRYGELPRTLHAERPTPAVDWSGGAVRLLREHTPWPVTGDRRRAAVSSFGV<br>SGTNVHLVLEQAPEPDAPPALPPAVHPDTMPVLLSARTPSALRRQAERVLDAVRADPGLPVPDLAAALATTRTAFPRRAAFVVEGRA<br>ELERRLRSFADGEDGGAEPDEVPAKRPLWFICSGMGTQWRGMGLSLMRLDSFRESILRSDEAVKPLGVKVSDLLLSTDERTFDDIVHA<br>FVSLTAIQIALIDLLTSVGLKPDGIIGHSLGEVACGYADGCLSQREAVLAAYWRGQCIKDAHLPPGSMAAVGLSWEECKQRCPAGVVPAC<br>HNSEDTVTISGPQAAVNEFVEQLKQEGVFAKEVRTGGLAFHSYFMEGIAPTLLQALKKVIREPRPRSARWLSTSIPEAQWQSSLARTSS<br>AEYNVNNLVSPVLFQEALWHIPEHAVVLEIAPHALLQAVLKRGVKSSCTIIPLMKRDHKDNLEFFLTNLGKVHLTGVPVDWREFFAGG<br>PTRPVDLPVYPFEHRRYWLASAPTRTGGATSVPEAPAPPTGDTPGPDRLRADLAPLTDDERERRLTDLVRGEIAAVAGFDGPHEIEAH<br>RSMTDLGLDSVSAVDLSTRLGARTGLDLPASLAFDHPTSTAIARHLLAALRTQGLTPESGQDAGTDGGTEGGKHAGIRAAEGTVFGALA<br>RLEASVDRDRPDAAARDRLVERLRALTARLTEGPAGGPSADDTSIGDRIGEATTVDQLFDLIDNELKGPLEHHHHHHHH*

Construct	Amino acid sequence
YZ045	MSAWSHPQFEKGGGSGGGSGGSAWSHPQFEKGAGSVTQVDDIRPLSAVLRAHALDRPDKVAFADEERHVTYARLAERTGRLAGHLAG<br>HGLRRGDRVAILLGNSVTTVESYLAVTRAAAVGVPVNPQSSDAELAHQLDDSGARFVITDGPHLDQVERLRATREGIRVVLARGYGPGG<br>GPHGPGPEAPASEGAPALDGTSAPDGAPGPDGPLSFEELAGSEPAQAPRDDLGLDEPAWILYTSGTTGAAKGVVSHQRACLWSVRSSY<br>QGVLGLGPDDRLLWPLPLFHSLAHILCVLGVTVSGATARILPGFGARDVLDALRAEPCTMLLGVPSMYLRLVAAVEAVEEAGGSVPKPR<br>LCVVTGAATGPELASAVERVLGAPLVNSYGATETCGAITLSRPDSERPPGTVGTAVPGSELRLVDPRTGRDARTGDEGEVLVRSPGLLL<br>RYHDRPEETAAALRDGWYRTGDLARRDAAGRLTITGRVKELVIRAGENIQPGEIEDVLRTVPGIADAAVTGRPDEALGEVPVAYVVPA<br>AGGWSPAEALAACRERLSYFKVPAELYEIAALPVTGSGKVTRRALPGLPARLRALGTGHHERLWRTTWVQCPAPDAAAEPAGAARW<br>AVIGAEVPAFAEAVRRRGGVAETFPDPAAARAAGPFDGHLLTAPAPDPDAPAEAAAESPLRTAEGPPAARTLLLTRSGVSTGPDDTPD<br>PTASAVRARLLARGGDGTVLVDLGPEDPRPTGGLAEAEAEAEAEADGAFAGAAARDADTGDAPGSDVPSPDVEAWLAVLSAPAGE<br>TEFALRSGAALVPRLARVRAAGEVALPSGGPGAALVTGAGTGISRVLATHLVVNHGVRDLVLADAGEEGDPGAVATLAADLTGLGATV<br>TVRTGRPDTAERASALLGALGPDRPFALVVHPVTAPADAEAAGRLLDLTRRSEGTTLVLCGRAAPGDPSEAAAAAHAAALTHDVAHA<br>TFLAFGPVAPDAPQPGLAALTAREAGDALDAVRATAGGPALVLRTDPWTLRAPGAERLRRMLPRYAEDTAAPAALDEGLRERLGVLP<br>GRARQRALTALVREEAAGVLGLDAPRRIDAGLAFTRLGLTSLTAVALRDRLAARTGLRLPVTLAFDHPTPAAVAAVLDGELFGARPAT<br>DAARRTSEDAGTSHRTGPRHDAHDPVVVIGMSCRYPGGIASPDDLWRFVAEGRDAIGPFPTDRGWDLERLLSGDGTAPGTSATGSGG<br>FLDAPGDFDAAFFGVSPREALAMDPQQRLLLEVGWEAVERAGIDPTSLRHTDTGVYVGLMFHDYAQHTGDLPEDLERLLGTGTAGSV<br>ASGRLAYTLGLNGPALTVDTACSSSLVAVHLAAQALRRGDCSLALAGGVAVMATPSTFVEFSRQHGLAADGRCKAFGEGADGTGWSEG<br>VGVVALERLSDARRHGHPVLAVIRGSAVNQDGASNGLTAPSGPAQQQVIRRALAAAGLTPGDVDAVEAHGTGTVLGDPIEANALLATY<br>GRGRDPERPLLLGSFKSNIGHAQSAAGVGAVIKMVQAIRYGELPRTLHAERPTPAVDWSGGAVRLLREHTPWPVTGDRRRAAVSSFGV<br>SGTNVHLVLEQAPEPDAPPALPPAVHPDTMPVLLSARTPSALRRQAERVLDAVRADPGLPVPDLAAALATTRTAFPRRAAFVVEGRA<br>ELERRLRSFADGEDGGAEPDEVPNKRPLWFICSGMGTQWRGMGLSLMRLDSFRESILRSDEAVKPLGVKVSDLLLSTDERTFDDIVHA<br>FVSLTAIQIALIDLLTSVGLKPDGIIGHSLGEVACGYADGCLSQREAVLAAYWRGQCIKDAHLPPGSMAAVGLSWEECKQRCPAGVVPAC<br>HNSEDTVTISGPQAAVNEFVEQLKQEGVFAKEVRTGGLAFHSYFMEGIAPTLLQALKKVIREPRPRSARWLSTSIPEAQWQSSLARTSS<br>AEYNVNNLVSPVLFQEALWHIPEHAVVLEIAPHALLQAVLKRGVKSSCTIIPLMKRDHKDNLEFFLTNLGKVHLTGVPVDWREFFAGG<br>PTRPVDLPVYPFEHRRYWLASAPTRTGGATSVPEAPAPPTGDTPGPDRLRADLAPLTDDERERRLTDLVRGEIAAVAGFDGPHEIEAH<br>RSMTDLGLDSVSAVDLSTRLGARTGLDLPASLAFDHPTSTAIARHLLAALRTQGLTPESGQDAGTDGGTEGGKHAGIRAAEGTVFGALA<br>RLEASVDRDRPDAAARDRLVERLRALTARLTEGPAGGPSADDTSIGDRIGEATTVDQLFDLIDNELKGPLEHHHHHHHH*
YZ046	MSGDNGMTEEKLRRYLKRTVTELDSVTARLREVEHRAGDPVVVIGMSCRYPGGIASPDDLWRFVAEGRDAIGPFPTDRGWDLERLLS<br>GDGTAPGTSATGSGGFLDAPGDFDAAFFGVSPREALAMDPQQRLLLEVGWEAVERAGIDPTSLRHTDTGVYVGLMFHDYAQHTGDLP<br>EDLERLLGTGTAGSVASGRLAYTLGLNGPALTVDTACSSSLVAVHLAAQALRRGDCSLALAGGVAVMATPSTFVEFSRQHGLAADGRC<br>KAFGEGADGTGWSEGVGVVALERLSDARRHGHPVLAVIRGSAVNQDGASNGLTAPSGPAQQQVIRRALAAAGLTPGDVDAVEAHGTG<br>TVLGDPIEANALLATYGRGRDPERPLLLGSFKSNIGHAQSAAGVGAVIKMVQAIRYGELPRTLHAERPTPAVDWSGGAVRLLREHTPW<br>PVTGDRRRAAVSSFGVSGTNVHLVLEQAPEPDAPPALPPAVHPDTMPVLLSARTPSALRRQAERVLDAVRADPGLPVPDLAAALATTR<br>TAFPRRAAFVVEGRAELERRLRSFADGEDGGAEPDEVPANKRPLWFICSGMGTQWRGMGLSLMRLDSFRESILRSDEAVKPLGVKVS<br>DLLLSTDERTFDDIVHAFVSLTAIQIALIDLLTSVGLKPDGIIGHSLGEVACGYADGCLSQREAVLAAYWRGQCIKDAHLPPGSMAAVGLS<br>WEECKQRCPAGVVPACHNSEDTVTISGPQAAVNEFVEQLKQEGVFAKEVRTGGLAFHSYFMEGIAPTLLQALKKVIREPRPRSARWLS<br>TSIPEAQWQSSLARTSSAEYNVNNLVSPVLFQEALWHIPEHAVVLEIAPHALLQAVLKRGVKSSCTIIPLMKRDHKDNLEFFLTNLGKV<br>HLTGVPVDWREFFAGGPTRPVDLPVYPFEHRRYWLASAPTRTGGATSVPEAPAPPTGDTPGPDRLRADLAPLTDDERERRLTDLVRG<br>EIAAVAGFDGPHEIEAHRSMTDLGLDSVSAVDLSTRLGARTGLDLPASLAFDHPTSTAIARHLLAALRTQGLTPESGQDAGTDGGTEGG<br>KHAGIRAAEGTVFGALARLEASVDRDRPDAAARDRLVERLRALTARLTEGPAGGPSADDTSIGDRIGEATTVDQLFDLIDNELKGPLEH<br>HHHHH*

Table S8. Amino acid sequences of PIKS-based constructs.

Construct	Amino acid sequence
SR008 (PIKS M5-TE)	MSAWSHPQFEKGGGSGGGSGGSAWSHPQFEKGAGSANNEDKLRDYLKRVTAELQQNTRRLREIEGRTHEPVAIVGMACRLPGGVASP<br>EDLWQLVAGDGDAISEFPQDRGWDVEGLYDPDPDASGRTYCRSGGFLHDAGEFDADFFGISPREALAMDPQQRLSLTTAWEAIESAGI<br>DPTALKGSGLGVFVGGWHTGYTSGQTTAVQSPELEGHLVSGAALGFLSGRIAYVLGTDGPALTVDTACSSSLVALHLAVQALRKGECD<br>MALAGGVTVMPNADLFVQFSRQRGLAADGRSKAFATSADGFGPAEGAGVLLVERLSDARRNGHRILAVVRGSAVNQDGASNGLTAPH<br>GPSQQRVIRRALADARLAPGDVDVVEAHGTGTRLGDPIEAQALIATYGQEKSSEQPLRLGALKSNIGHTQAAAGVAGVIKMVQAMRHG<br>LLPKTLHVDEPSDQIDWSAGTVELLTEAVDWPEKQDGGLRRAAVSSFGISGTNAHVVLEEAPAVEDSPAVEPPAGGGVVPWPVSAKTP<br>AALDAQIGQLAAYADGRTDVDPAVAARALVDSRTAMEHRAVAVGDSREALRDALRMPEGLVRGTSSDVGRVAFVFPGQGTQWAGM<br>GAELLDSSPEFAASMAECETALSRYVDWSLEAVVRQEPGAPTLDRVDVVQPVTFAVMVSLAKVWQHHGITPQAVVGHSQGEIAAAYV<br>AGALTLDDAARVVTLRSKSIAAHLAGKGGMISLALDEAAVLKRLSDFDGLSVAAVNGPTATVVSGDPTQIEELARTCEADGVRARIIPV<br>DYASHSRQVEIIEKELAEVLAGLAPQAPHVPFFSTLEGTWITEPVLDGTYWYRNLRHRVGFAPAVETLAVDGFTHFIEVSAHPVLTMTL<br>PETVTGLGTLRREQGGQERLVTSLAEAWANGLTIDWAPILPTATGHHPELPTYAFQTERFWLQSSAPTSAADDWRYRVEWKPLTASG<br>QADLSGRWIVAVGSEPEAELLGALKAAGAEVDVLEAGADDDREALAARLTALTTGDGFTGVVSLLDDLVPQVAWVQALGDAGIKAPL<br>WSVTQGAVSVGRLDTPADPDRAMLWGLGRVVALEHPERWAGLVDLPAQPDAAALAHLVTALSGATGEDQIAIRTTGLHARRLARAP<br>LHGRRPTRDWQPHGTVLITGGTGALGSHAARWMAHHGAEHLLLVSRSGEQAPGATQLTAELTASGARVTIAACDVADPHAMRTLLD<br>AIPAETPLTAVVHTAGAPGGDPLDVTGPEDIARILGAKTSGAEVLDDLLRGTPLDAFVLYSSNAGVWGSGSQGVYAAANAHLDALAAR<br>RRARGETATSVAWGLWAGDGMGRGADDAYWQRRGIRPMSPDRALDELAKALSHDETFVAVADVDWERFAPAFTVSRPSLLLDGVP<br>EARQALAAPVGAPAPGDAAVAPTGQSSALAAITALPEPERRPALLTLVRTHAAAVLGHSSPDRVAPGRAFTELGFDSLTAVQLRNQLST<br>VVGNRLPATTVFDHPTPAALAAHLHEAYSGADTGAGAGMFRALFRQAVEDDRYGEFLDVLAEASAFRPQFASPEACSERLDPVLLAGG<br>PTDRAEGRAVLVGCTGTAANGGPHEFLRLSTSFQEERDFLAVPLPGYGTGTGTGTALLPADLDTALDAQARAILRAAGDAPVVLLGHS<br>GGALLAHELAFRLERAHGAPPAGIVLVDPYPPGHQEPIEVWSRQLGEGLFAGELEPMSDARLLAMGRYARFLAGPRPGRSSAPVLLVR<br>ASEPLGDWQEERGDWRAHWDLPHTVADVPGDHFTMMRDHAPAVAEAVLSWLDAIEGIEGAGKLEHHHHHHHH
SR015 (PIKS M5*-TE)	MSAWSHPQFEKGGGSGGGSGGSAWSHPQFEKGAGSANNEDKLRDYLKRVTAELQQNTRRLREIEGRTHEPVAIVGMACRLPGGVASP<br>EDLWQLVAGDGDAISEFPQDRGWDVEGLYDPDPDASGRTYCRSGGFLHDAGEFDADFFGISPREALAMDPQQRLSLTTAWEAIESAGI<br>DPTALKGSGLGVFVGGWHTGYTSGQTTAVQSPELEGHLVSGAALGFLSGRIAYVLGTDGPALTVDTACSSSLVALHLAVQALRKGECD<br>MALAGGVTVMPNADLFVQFSRQRGLAADGRSKAFATSADGFGPAEGAGVLLVERLSDARRNGHRILAVVRGSAVNQDGASNGLTAPH<br>GPSQQRVIRRALADARLAPGDVDVVEAHGTGTRLGDPIEAQALIATYGQEKSSEQPLRLGALKSNIGHTQAAAGVAGVIKMVQAMRHG<br>LLPKTLHVDEPSDQIDWSAGTVELLTEAVDWPEKQDGGLRRAAVSSFGISGTNAHVVLEEAPAVEDSPAVEPPAGGGVVPWPVSAKTP<br>AALDAQIGQLAAYADGRTDVDPAVAARALVDSRTAMEHRAVAVGDSREALRDALRMPEGLVRGTSSNKRPLWFICSGMGTQWRGM<br>GLSLMRLDSFRESILRSDEAVKPLGVKVSDLLLSTDERTFDDIVHAFVSLTAIQIALIDLLTSVGLKPDGIIGHSLGEVACGYADGCLSQRE<br>AVLAAYWRGQCIKDAHLPPGSMAAVGLSWEECKQRCPAGVVPACHNSEDTVTISGPQAAVNEFVEQLKQEGVFAKEVRTGGLAFHSY<br>FMEGIAPTLLQALKKVIREPRPRSARWLSTSIPEAQWQSSLARTSSAEYNVNNLVSPVLFQEALWHIPEHAVVLEIAPHALLQAVLKRG<br>VKSSCTIIPLMKRDHKDNLEFFLTNLGKVHLTGLTIDWAPILPTATGHHPELPTYAFQTERFWLQSSAPTSAADDWRYRVEWKPLTAS<br>GQADLSGRWIVAVGSEPEAELLGALKAAGAEVDVLEAGADDDREALAARLTALTTGDGFTGVVSLLDDLVPQVAWVQALGDAGIKAP<br>LWSVTQGAVSVGRLDTPADPDRAMLWGLGRVVALEHPERWAGLVDLPAQPDAAALAHLVTALSGATGEDQIAIRTTGLHARRLARA<br>PLHGRRPTRDWQPHGTVLITGGTGALGSHAARWMAHHGAEHLLLVSRSGEQAPGATQLTAELTASGARVTIAACDVADPHAMRTLL<br>DAIPAETPLTAVVHTAGAPGGDPLDVTGPEDIARILGAKTSGAEVLDDLLRGTPLDAFVLYSSNAGVWGSGSQGVYAAANAHLDALAA<br>RRRARGETATSVAWGLWAGDGMGRGADDAYWQRRGIRPMSPDRALDELAKALSHDETFVAVADVDWERFAPAFTVSRPSLLLDGV<br>PEARQALAAPVGAPAPGDAAVAPTGQSSALAAITALPEPERRPALLTLVRTHAAAVLGHSSPDRVAPGRAFTELGFDSLTAVQLRNQLS<br>TVVGNRLPATTVFDHPTPAALAAHLHEAYSGADTGAGAGMFRALFRQAVEDDRYGEFLDVLAEASAFRPQFASPEACSERLDPVLLAG<br>GPTDRAEGRAVLVGCTGTAANGGPHEFLRLSTSFQEERDFLAVPLPGYGTGTGTGTALLPADLDTALDAQARAILRAAGDAPVVLLGH<br>SGGALLAHELAFRLERAHGAPPAGIVLVDPYPPGHQEPIEVWSRQLGEGLFAGELEPMSDARLLAMGRYARFLAGPRPGRSSAPVLLVR<br>ASEPLGDWQEERGDWRAHWDLPHTVADVPGDHFTMMRDHAPAVAEAVLSWLDAIEGIEGAGKLEHHHHHHHH

Table S9. Amino acid sequences of DEBS-based constructs.

**Construct**	**Amino acid sequence**
AR268 (DEBS M5-M6-TE)	MGSSGDNGMTEEKLRRYLKRTVTELDSVTARLREVEHRAGEPIAIVGMACRFPGDVDSPESFWEFVSGGGDAIAEAPADRGWEPDPD<br>ARLGGMLAAAGDFDAGFFGISPREALAMDPQQRIMLEISWEALERAGHDPVSLRGSATGVFTGVGTVDYGPRPDEAPDEVLGYVGTGT<br>ASSVASGRVAYCLGLEGPAMTVDTACSSGLTALHLAMESLRRDECGLALAGGVTVMSSPGAFTEFRSQGGLAADGRCKPFSKAADGFG<br>LAEGAGVLVLQRLSAARREGRPVLAVLRGSAVNQDGASNGLTAPSGPAQQRVIRRALENAGVRAGDVDYVEAHGTGTRLGDPIEVHAL<br>LSTYGAERDPDDPLWIGSVKSNIGHTQAAAGVAGVMKAVLALRHGEMPRTLHFDEPSPQIEWDLGAVSVVSQARSWPAGERPRRAGV<br>SSFGISGTNAHVIVEEAPEADEPEPAPDSGPVPLVLSGRDEQAMRAQAGRLADHLAREPRNSLRDTGFTLATRRSAWEHRAVVVGDRD<br>DALAGLRAVADGRIADRTATGQARTRRGVAMVFPGQGAQWQGMARDLLRESQVFADSIRDCERALAPHVDWSLTDLLSGARPLDRV<br>DVVQPALFAVMVSLAALWRSHGVEPAAVVGHSQGEIAAAHVAGALTLEDAAKLVAVRSRVLRRLGGQGGMASFGLGTEQAAERIGRF<br>AGALSIASVNGPRSVVVAGESGPLDELIAECEAEGITARRIPVDYASHSPQVESLREELLTELAGISPVSADVALYSTTTGQPIDTATMDT<br>AYWYANLREQVRFQDATRQLAEAGFDAFVEVSPHPVLTVGIEATLDSALPADAGACVVGTLRRDRGGLADFHTALGEAYAQGVEVD<br>WSPAFADARPVELPVYPFQRQRYWLPIPTGGRARDEDDDWRYQVVWREAEWESASLAGRVLLVTGPGVPSELSDAIRSGLEQSGATV<br>LTCDVESRSTIGTALEAADTDALSTVVSLLSRDGEAVDPSLDALALVQALGAAGVEAPLWVLTRNAVQVADGELVDPAQAMVGGLGRV<br>VGIEQPGRWGGLVDLVDADAASIRSLAAVLADPRGEEQVAIRADGIKVARLVPAPARAARTRWSPRGTVLVTGGTGGIGAHVARWLA<br>RSGAEHLVLLGRRGADAPGASELREELTALGTGVTIAACDVADRARLEAVLAAERAEGRTVSAVMHAAGVSTSTPLDDLTEAEFTEIA<br>DVKVRGTVNLDELCPDLDAFVLFSSNAGVWGSPGLASYAAANAFLDGFARRRRSEGAPVTSIAWGLWAGQNMAGDEGGEYLRSQGLR<br>AMDPDRAVEELHITLDHGQTSVSVVDMDRRRFVELFTAARHRPLFDEIAGARAEARQSEEGPALAQRLAALSTAERREHLAHLIRAEV<br>AAVLGHGDDAAIDRDRAFRDLGFDSMTAVDLRNRLAAVTGVREAATVVFDHPTITRLADHYLERLVGAAEAEQAPALVREVPKDADD<br>PIAIVGMACRFPGGVHNPGELWEFIVGGGDAVTEMPTDRGWDLDALFDPDPQRHGTSYSRHGAFLDGAADFDAAFFGISPREALAMD<br>PQQRQVLETTWELFENAGIDPHSLRGSDTGVFLGAAYQGYGQDAVVPEDSEGYLLTGNSSAVVSGRVAYVLGLEGPAVTVDTACSSSLV<br>ALHSACGSLRDGDCGLAVAGGVSVMAGPEVFTEFSRQGGLAVDGRCKAFSAEADGFGFAEGVAVVLLQRLSDARRAGRQVLGVVAGSA<br>INQDGASNGLAAPSGVAQQRVIRKAWARAGITGADVAVVEAHGTGTRLGDPVEASALLATYGKSRGSSGPVLLGSVKSNIGHAQAAAG<br>VAGVIKVVLGLNRGLVPPMLCRGERSPLIEWSSGGVELAEAVSPWPPAADGVRRAGVSAFGVSGTNAHVIIAEPPEPEPLPEPGPVGVL<br>AAANSVPVLLSARTETALAAQARLLESAVDDSVPLTALASALATGRAHLPRRAALLAGDHEQLRGQLRAVAEGVAAPGATTGTASAGG<br>VVFVFPGQGAQWEGMARGLLSVPVFAESIAECDAVLSEVAGFSASEVLEQRPDAPSLERVDVVQPVLFSVMVSLARLWGACGVSPSAVI<br>GHSQGEIAAAVVAGVLSLEDGVRVVALRAKALRALAGKGGMVSLAAPGERARALIAPWEDRISVAAVNSPSSVVVSGDPEALAELVARC<br>EDEGVRAKTLPVDYASHSRHVEEIRETILADLDGISARRAAIPLYSTLHGERRDGADMGPRYWYDNLRSQVRFDEAVSAAVADGHATF<br>VEMSPHPVLTAAVQEIAADAVAIGSLHRDTAEEHLIAELARAHVHGVAVDWRNVFPAAPPVALPNYPFEPQRYWLAPEVSDQLADSR<br>YRVDWRPLATTPVDLEGGFLVHGSAPESLTSAVEKAGGRVVPVASADREALAAALREVPGEVAGVLSVHTGAATHLALHQSLGEAGVR<br>APLWLVTSRAVALGESEPVDPEQAMVWGLGRVMGLETPERWGGLVDLPAEPAPGDGEAFVACLGADGHEDQVAIRDHARYGRRLVR<br>APLGTRESSWEPAGTALVTGGTGALGGHVARHLARCGVEDLVLVSRRGVDAPGAAELEAELVALGAKTTITACDVADREQLSKLLEEL<br>RGQGRPVRTVVHTAGVPESRPLHEIGELESVCAAKVTGARLLDELCPDAETFVLFSSGAGVWGSANLGAYSAANAYLDALAHRRRAEG<br>RAATSVAWGAWAGEGMATGDLEGLTRRGLRPMAPERAIRALHQALDNGDTCVSIADVDWERFAVGFTAARPRPLLDELVTPAVGAV<br>PAVQAAPAREMTSQELLEFTHSHVAAILGHSSPDAVGQDQPFTELGFDSLTAVGLRNQLQQATGLALPATLVFEHPTVRRLADHIGQQ<br>LDSGTPAREASSALRDGYRQAGVSGRVRSYLDLLAGLSDFREHFDGSDGFSLDLVDMADGPGEVTVICCAGTAAISGPHEFTRLAGALR<br>GIAPVRAVPQPGYEEGEPLPSSMAAVAAVQADAVIRTQGDKPFVVAGHSAGALMAYALATELLDRGHPPRGVVLIDVYPPGHQDAMN<br>AWLEELTATLFDRETVRMDDTRLTALGAYDRLTGQWRPRETGLPTLLVSAGEPMGPWPDDSWKPTWPFEHDTVAVPGDHFTMVQ<br>EHADAIARHIDAWLGGGNSLEHHHHHHHH
SR022 (DEBS M5*-M6-TE)	MSAWSHPQFEKGGGSGGGSGGSAWSHPQFEKGAGSSGDNGMTEEKLRRYLKRTVTELDSVTARLREVEHRAGEPIAIVGMACRFPGD<br>VDSPESFWEFVSGGGDAIAEAPADRGWEPDPDARLGGMLAAAGDFDAGFFGISPREALAMDPQQRIMLEISWEALERAGHDPVSLRG<br>SATGVFTGVGTVDYGPRPDEAPDEVLGYVGTGTASSVASGRVAYCLGLEGPAMTVDTACSSGLTALHLAMESLRRDECGLALAGGVTV<br>MSSPGAFTEFRSQGGLAADGRCKPFSKAADGFGLAEGAGVLVLQRLSAARREGRPVLAVLRGSAVNQDGASNGLTAPSGPAQQRVIRR<br>ALENAGVRAGDVDYVEAHGTGTRLGDPIEVHALLSTYGAERDPDDPLWIGSVKSNIGHTQAAAGVAGVMKAVLALRHGEMPRTLHFD<br>EPSPQIEWDLGAVSVVSQARSWPAGERPRRAGVSSFGISGTNAHVIVEEAPEADEPEPAPDSGPVPLVLSGRDEQAMRAQAGRLADHL<br>AREPRNSLRDTGFTLATRRSAWEHRAVVVGDRDDALAGLRAVADGRIADRTATGQARTNKRPLWFICSGMGTQWRGMGLSLMRLD<br>SFRESILRSDEAVKPLGVKVSDLLLSTDERTFDDIVHAFVSLTAIQIALIDLLTSVGLKPDGIIGHSLGEVACGYADGCLSQREAVLAAYWR<br>GQCIKDAHLPPGSMAAVGLSWEECKQRCPAGVVPACHNSEDTVTISGPQAAVNEFVEQLKQEGVFAKEVRTGGLAFHSYFMEGIAPTL<br>LQALKKVIREPRPRSARWLSTSIPEAQWQSSLARTSSAEYNVNNLVSPVLFQEALWHIPEHAVVLEIAPHALLQAVLKRGVKSSCTIIPL<br>MKRDHKDNLEFFLTNLGKVHLTGVEVDWSPAFADARPVELPVYPFQRQRYWLPIPTGGRARDEDDDWRYQVVWREAEWESASLAG<br>RVLLVTGPGVPSELSDAIRSGLEQSGATVLTCDVESRSTIGTALEAADTDALSTVVSLLSRDGEAVDPSLDALALVQALGAAGVEAPLWV<br>LTRNAVQVADGELVDPAQAMVGGLGRVVGIEQPGRWGGLVDLVDADAASIRSLAAVLADPRGEEQVAIRADGIKVARLVPAPARAAR<br>TRWSPRGTVLVTGGTGGIGAHVARWLARSGAEHLVLLGRRGADAPGASELREELTALGTGVTIAACDVADRARLEAVLAAERAEGRT<br>VSAVMHAAGVSTSTPLDDLTEAEFTEIADVKVRGTVNLDELCPDLDAFVLFSSNAGVWGSPGLASYAAANAFLDGFARRRRSEGAPVT<br>SIAWGLWAGQNMAGDEGGEYLRSQGLRAMDPDRAVEELHITLDHGQTSVSVVDMDRRRFVELFTAARHRPLFDEIAGARAEARQSE<br>EGPALAQRLAALSTAERREHLAHLIRAEVAAVLGHGDDAAIDRDRAFRDLGFDSMTAVDLRNRLAAVTGVREAATVVFDHPTITRLA<br>DHYLERLVGAAEAEQAPALVREVPKDADDPIAIVGMACRFPGGVHNPGELWEFIVGGGDAVTEMPTDRGWDLDALFDPDPQRHGTS<br>YSRHGAFLDGAADFDAAFFGISPREALAMDPQQRQVLETTWELFENAGIDPHSLRGSDTGVFLGAAYQGYGQDAVVPEDSEGYLLTGN<br>SSAVVSGRVAYVLGLEGPAVTVDTACSSSLVALHSACGSLRDGDCGLAVAGGVSVMAGPEVFTEFSRQGGLAVDGRCKAFSAEADGFGF<br>AEGVAVVLLQRLSDARRAGRQVLGVVAGSAINQDGASNGLAAPSGVAQQRVIRKAWARAGITGADVAVVEAHGTGTRLGDPVEASAL<br>LATYGKSRGSSGPVLLGSVKSNIGHAQAAAGVAGVIKVVLGLNRGLVPPMLCRGERSPLIEWSSGGVELAEAVSPWPPAADGVRRAGVS<br>AFGVSGTNAHVIIAEPPEPEPLPEPGPVGVLAAANSVPVLLSARTETALAAQARLLESAVDDSVPLTALASALATGRAHLPRRAALLAG<br>DHEQLRGQLRAVAEGVAAPGATTGTASAGGVVFVFPGQGAQWEGMARGLLSVPVFAESIAECDAVLSEVAGFSASEVLEQRPDAPSLE<br>RVDVVQPVLFSVMVSLARLWGACGVSPSAVIGHSQGEIAAAVVAGVLSLEDGVRVVALRAKALRALAGKGGMVSLAAPGERARALIAP<br>WEDRISVAAVNSPSSVVVSGDPEALAELVARCEDEGVRAKTLPVDYASHSRHVEEIRETILADLDGISARRAAIPLYSTLHGERRDGAD<br>MGPRYWYDNLRSQVRFDEAVSAAVADGHATFVEMSPHPVLTAAVQEIAADAVAIGSLHRDTAEEHLIAELARAHVHGVAVDWRNVF<br>PAAPPVALPNYPFEPQRYWLAPEVSDQLADSRYRVDWRPLATTPVDLEGGFLVHGSAPESLTSAVEKAGGRVVPVASADREALAAAL

Construct	Amino acid sequence
	REVPGEVAGVLSVHTGAATHLALHQSLGEAGVRAPLWLVTSRAVALGESEPVDPEQAMVWGLGRVMGLETPERWGGLVDLPAEPAP<br>GDGEAFVACLGADGHEDQVAIRDHARYGRRLVRAPLGTRESSWEPAGTALVTGGTGALGGHVARHLARCGVEDLVLVSRRGVDAPGA<br>AELEAELVALGAKTTITACDVADREQLSKLLEELRGQGRPVRTVVHTAGVPESRPLHEIGELESVCAAKVTGARLLDELCPDAETFVLFS<br>SGAGVWGSANLGAYSAANAYLDALAHRRRAEGRAATSVAWGAWAGEGMATGDLEGLTRRGLRPMAPERAIRALHQALDNGDTCVSI<br>ADVDWERFAVGFTAARPRPLLDELVTPAVGAVPAVQAAPAREMTSQELLEFTHSHVAAILGHSSPDAVGQDQPFTELGFDSLTAVGLR<br>NQLQQATGLALPATLVFEHPTVRRLADHIGQQLDSGTPAREASSALRDGYRQAGVSGRVRSYLDLLAGLSDFREHFDGSDGFSLDLVD<br>MADGPGEVTVICCAGTAAISGPHEFTRLAGALRGIAPVRAVPQPGYEEGEPLPSSMAAVAAVQADAVIRTQGDKPFVVAGHSAGALMA<br>YALATELLDRGHPPRGVVLIDVYPPGHQDAMNAWLEELTATLFDRETVRMDDTRLTALGAYDRLTGQWRPRETGLPTLLVSAGEPM<br>GPWPDDSWKPTWPFEHDTVAVPGDHFTMVQEHADAIARHIDAWLGGGNSLEHHHHHHHH
MJD105 (DEBS M5-M6*-TE)	MGSSGDNGMTEEKLRRYLKRTVTELDSVTARLREVEHRAGEPIAIVGMACRFPGDVDSPESFWEFVSGGGDAIAEAPADRGWEPDPD<br>ARLGGMLAAAGDFDAGFFGISPREALAMDPQQRIMLEISWEALERAGHDPVSLRGSATGVFTGVGTVDYGPRPDEAPDEVLGYVGTGT<br>ASSVASGRVAYCLGLEGPAMTVDTACSSGLTALHLAMESLRRDECGLALAGGVTVMSSPGAFTEFRSQGGLAADGRCKPFSKAADGFG<br>LAEGAGVLVLQRLSAARREGRPVLAVLRGSAVNQDGASNGLTAPSGPAQQRVIRRALENAGVRAGDVDYVEAHGTGTRLGDPIEVHAL<br>LSTYGAERDPDDPLWIGSVKSNIGHTQAAAGVAGVMKAVLALRHGEMPRTLHFDEPSPQIEWDLGAVSVVSQARSWPAGERPRRAGV<br>SSFGISGTNAHVIVEEAPEADEPEPAPDSGPVPLVLSGRDEQAMRAQAGRLADHLAREPRNSLRDTGFTLATRRSAWEHRAVVVGDRD<br>DALAGLRAVADGRIADRTATGQARTRRGVAMVFPGQGAQWQGMARDLLRESQVFADSIRDCERALAPHVDWSLTDLLSGARPLDRV<br>DVVQPALFAVMVSLAALWRSHGVEPAAVVGHSQGEIAAAHVAGALTLEDAAKLVAVRSRVLRRLGGQGGMASFGLGTEQAAERIGRF<br>AGALSIASVNGPRSVVVAGESGPLDELIAECEAEGITARRIPVDYASHSPQVESLREELLTELAGISPVSADVALYSTTTGQPIDTATMDT<br>AYWYANLREQVRFQDATRQLAEAGFDAFVEVSPHPVLTVGIEATLDSALPADAGACVVGTLRRDRGGLADFHTALGEAYAQGVEVD<br>WSPAFADARPVELPVYPFQRQRYWLPIPTGGRARDEDDDWRYQVVWREAEWESASLAGRVLLVTGPGVPSELSDAIRSGLEQSGATV<br>LTCDVESRSTIGTALEAADTDALSTVVSLLSRDGEAVDPSLDALALVQALGAAGVEAPLWVLTRNAVQVADGELVDPAQAMVGGLGRV<br>VGIEQPGRWGGLVDLVDADAASIRSLAAVLADPRGEEQVAIRADGIKVARLVPAPARAARTRWSPRGTVLVTGGTGGIGAHVARWLA<br>RSGAEHLVLLGRRGADAPGASELREELTALGTGVTIAACDVADRARLEAVLAAERAEGRTVSAVMHAAGVSTSTPLDDLTEAEFTEIA<br>DVKVRGTVNLDELCPDLDAFVLFSSNAGVWGSPGLASYAAANAFLDGFARRRRSEGAPVTSIAWGLWAGQNMAGDEGGEYLRSQGLR<br>AMDPDRAVEELHITLDHGQTSVSVVDMDRRRFVELFTAARHRPLFDEIAGARAEARQSEEGPALAQRLAALSTAERREHLAHLIRAEV<br>AAVLGHGDDAAIDRDRAFRDLGFDSMTAVDLRNRLAAVTGVREAATVVFDHPTITRLADHYLERLVGAAEAEQAPALVREVPKDADD<br>PIAIVGMACRFPGGVHNPGELWEFIVGGGDAVTEMPTDRGWDLDALFDPDPQRHGTSYSRHGAFLDGAADFDAAFFGISPREALAMD<br>PQQRQVLETTWELFENAGIDPHSLRGSDTGVFLGAAYQGYGQDAVVPEDSEGYLLTGNSSAVVSGRVAYVLGLEGPAVTVDTACSSSLV<br>ALHSACGSLRDGDCGLAVAGGVSVMAGPEVFTEFSRQGGLAVDGRCKAFSAEADGFGFAEGVAVVLLQRLSDARRAGRQVLGVVAGSA<br>INQDGASNGLAAPSGVAQQRVIRKAWARAGITGADVAVVEAHGTGTRLGDPVEASALLATYGKSRGSSGPVLLGSVKSNIGHAQAAAG<br>VAGVIKVVLGLNRGLVPPMLCRGERSPLIEWSSGGVELAEAVSPWPPAADGVRRAGVSAFGVSGTNAHVIIAEPPEPEPLPEPGPVGVL<br>AAANSVPVLLSARTETALAAQARLLESAVDDSVPLTALASALATGRAHLPRRAALLAGDHEQLRGQLRAVAEGVAAPGATTGTASNKR<br>PLWFICSGMGTQWRGMGLSLMRLDSFRESILRSDEAVKPLGVKVSDLLLSTDERTFDDIVHAFVSLTAIQIALIDLLTSVGLKPDGIIGHS<br>LGEVACGYADGCLSQREAVLAAYWRGQCIKDAHLPPGSMAAVGLSWEECKQRCPAGVVPACHNSEDTVTISGPQAAVNEFVEQLKQE<br>GVFAKEVRTGGLAFHSYFMEGIAPTLLQALKKVIREPRPRSARWLSTSIPEAQWQSSLARTSSAEYNVNNLVSPVLFQEALWHIPEHAV<br>VLEIAPHALLQAVLKRGVKSSCTIIPLMKRDHKDNLEFFLTNLGKVHLTGVAVDWRNVFPAAPPVALPNYPFEPQRYWLAPEVSDQLA<br>DSRYRVDWRPLATTPVDLEGGFLVHGSAPESLTSAVEKAGGRVVPVASADREALAAALREVPGEVAGVLSVHTGAATHLALHQSLGEA<br>GVRAPLWLVTSRAVALGESEPVDPEQAMVWGLGRVMGLETPERWGGLVDLPAEPAPGDGEAFVACLGADGHEDQVAIRDHARYGR<br>RLVRAPLGTRESSWEPAGTALVTGGTGALGGHVARHLARCGVEDLVLVSRRGVDAPGAAELEAELVALGAKTTITACDVADREQLSKL<br>LEELRGQGRPVRTVVHTAGVPESRPLHEIGELESVCAAKVTGARLLDELCPDAETFVLFSSGAGVWGSANLGAYSAANAYLDALAHRR<br>RAEGRAATSVAWGAWAGEGMATGDLEGLTRRGLRPMAPERAIRALHQALDNGDTCVSIADVDWERFAVGFTAARPRPLLDELVTPA<br>VGAVPAVQAAPAREMTSQELLEFTHSHVAAILGHSSPDAVGQDQPFTELGFDSLTAVGLRNQLQQATGLALPATLVFEHPTVRRLADH<br>IGQQLDSGTPAREASSALRDGYRQAGVSGRVRSYLDLLAGLSDFREHFDGSDGFSLDLVDMADGPGEVTVICCAGTAAISGPHEFTRLA<br>GALRGIAPVRAVPQPGYEEGEPLPSSMAAVAAVQADAVIRTQGDKPFVVAGHSAGALMAYALATELLDRGHPPRGVVLIDVYPPGHQD<br>AMNAWLEELTATLFDRETVRMDDTRLTALGAYDRLTGQWRPRETGLPTLLVSAGEPMGPWPDDSWKPTWPFEHDTVAVPGDHFT<br>MVQEHADAIARHIDAWLGGGNSLEHHHHHHHH

Table S10. Amino acid sequences used for the prediction of the KS-AT didomain structure of VemG KS1-AT1 and KS2-AT2 with ColabFold[2].

Prediction	Amino acid sequence
VEMS KS1-AT1	DPVVVIGMSCRYPGGIASPDDLWRFVAEGRDAIGPFPTDRGWDLERLLSGDGTAPGTSATGSGGFLDAPGDFDAAFFGVSPREALAMD<br>PQQRLLLEVGWEAVERAGIDPTSLRHTDTGVYVGLMFHDYAQHTGDLPEDLERLLGTGTAGSVASGRLAYTLGLNGPALTVDTACSSS<br>LVAVHLAAQALRRGDCSLALAGGVAVMATPSTFVEFSRQHGLAADGRCKAFGEGADGTGWSEGVGVVALERLSDARRHGHPVLAVIR<br>GSAVNQDGASNGLTAPSGPAQQQVIRRALAAAGLTPGDVDAVEAHGTGTVLGDPIEANALLATYGRGRDPERPLLLGSFKSNIGHAQS<br>AAGVGAVIKMVQAIRYGELPRTLHAERPTPAVDWSGGAVRLLREHTPWPVTGDRRRAAVSSFGVSGTNVHLVLEQAPEPDAPPALPP<br>AVHPDTMPVLLSARTPSALRRQAERVLDAVRADPGLPVPDLAAALATTRTAFPRRAAFVVEGRAELERRLRSFADGEDGGAEPDEVP<br>AEPSLAIGFTGQGSQHPGMGRELYAAFPVFAEALDAAWAALDPHLERPLRDVMWAPDGTARAALLDRTDFTQAALFALEGALYRLVE<br>SWGVVPDVVLGHSVGALTAAHAAGVLSLPDAASLVAARGRLMAALPPGGAMTSIEATEDELRTELRATDGVLAIAAVNGPRSVVVSGE<br>EKAVRTVGEAFRARGRRVTALRVSHAFHSPLMDPMVEEFRAAAARVTYRPPLLPMISDLTGRPADPAHLRSPDHWVRHVRETVRFA<br>DAVRALPGQGVTAFLELGPDRQLTTMAAAGAPGSGPALCGGLRRGRSEVRSLLDAVAQAHVRGVPVDWREFFAGGPTRPVDLPVYPF<br>EHRRYWLASAPTRTGGATSVPEAPAPPTGDTP
VEMS KS2-AT2	EPIAIVGMACRYPGGVRTPEALWRLVLDEQDAISGFPTNRGWDIDGIYHPDPDRPGTCYAREGGFLHDAALFDAEFFGVSPREAQAMD<br>PQQRLLLETAWEAFERAGIDPTSLRGSDTGVFAGVVHHDYATARVPETLEPYLVTGLSGGVASGRIAYTFGFEGPAVTVDTACSSSLVA<br>LHQAAHALRSGECELALAGGVTIMATPRAFLSFSRQRGLSPDGRCRAFGAGADGTGWAEGAGMLLVERLSDARRKGHPVLAVLRGSA<br>VNQDGASNGLSAPNGPSQQRVIRKALAHAGLAARDVDVVEGHGTGTKLGDPIEAQALLATYGQERDAGLPLHLGSMKSNVGHSQAAA<br>GVGGVIKMIEAMRHGILPRTLHADEPTPHVDWSAGDIELLTRRRAWPETGRPRRAAVSSFGISGTNAHVILEAPPEEPARDAAETRPE<br>ARSREAAEGRREAAGQGRTETGPGPSATPPEAPGRARPPVPWPLSGRDAGALRDQIGRLRAHLDAAPADPEDVAHSLARRAVFRHRA<br>VLLAAPQAPAGGSPRAVTGVARPGGTALLFSGQGSQRVGMGSELYETYPVFAESFDAVAEHTGLPLKDVVLGGTPDGLLDRTRYAQPA<br>LFAVEVSLFRLVRALGLDVRAVVGHSVGEIAAAHVAGVMSMADACRLVEARGRLMDALPPGGAMVAVEVTEAEASAALAGLEDRVAV<br>AAVNGPASTVLSGEEGAVLKLADAWRERGVRTHRLTVSHAFHSPLMEPMVDAFREVVAGLDLHRPTLAGLPAEVVDPEYWVRHVRR<br>PVRFADAVARAREAGAVRWLEVGPGGVLTALAQRIVPDTEEHVFAAALRTDRPEPEALLVALSQVHVDGGTVDWSGLCAGGRLVDLP<br>TYPFQRQHYWIEDQPLPPTAPRPGTAPSGTGTAAEGAAAAEVPLSER

References

1. Buyachuihan, L., Zhao, Y., Schelhas, C. & Grininger, M. Docking Domain Engineering in a Modular Polyketide Synthase and Its Impact on Structure and Function. *ACS Chem. Biol.* **18**, 1500–1509 (2023).
2. Mirdita, M. *et al.* ColabFold: making protein folding accessible to all. *Nat. Methods* **19**, 679–682 (2022).
3. Rittner, A. *et al.* Chemoenzymatic synthesis of fluorinated polyketides. *Nat. Chem.* **14**, 1000–1006 (2022).
4. Rittner, A., Paithankar, K. S., Huu, K. V. & Grininger, M. Characterization of the Polyspecific Transferase of Murine Type I Fatty Acid Synthase (FAS) and Implications for Polyketide Synthase (PKS) Engineering. *ACS Chem. Biol.* **13**, 723–732 (2018).
5. Lowry, B. *et al. In Vitro* Reconstitution and Analysis of the 6-Deoxyerythronolide B Synthase. *J. Am. Chem. Soc.* **135**, 16809–16812 (2013).
6. Rittner, A., Paithankar, K. S., Drexler, D. J., Himmler, A. & Grininger, M. Probing the modularity of megasynthases by rational engineering of a fatty acid synthase Type I. *Protein Sci.* **28**, 414–428 (2019).
7. Englund, E. *et al.* Biosensor Guided Polyketide Synthases Engineering for Optimization of Domain Exchange Boundaries. *Nat. Commun.* **14**, 4871 (2023).
